# 6’-Fluoro[4.3.0]bicyclo nucleic acid: synthesis, biophysical properties and molecular dynamics simulations

**DOI:** 10.3762/bjoc.14.288

**Published:** 2018-12-20

**Authors:** Sibylle Frei, Andrei Istrate, Christian J Leumann

**Affiliations:** 1 Department of Chemistry and Biochemistry, University of Bern, Freiestrasse 3, 3012 Bern, Switzerland

**Keywords:** DNA/RNA affinity, fluorinated cyclopropanes, fluorinated nucleic acids, molecular dynamics simulations, sugar modified nucleosides

## Abstract

Here we report on the synthesis, biophysical properties and molecular modeling of oligonucleotides containing unsaturated 6’-fluoro[4.3.0]bicyclo nucleotides (6’F-bc^4,3^-DNA). Two 6’F-bc^4,3^ phosphoramidite building blocks (T and C) were synthesized starting from a previously described [3.3.0]bicyclic sugar. The conversion of this sugar to a *gem*-difluorinated tricyclic intermediate via difluorocarbene addition followed either by a NIS-mediated or Vorbrüggen nucleosidation yielded in both cases the β-tricyclic nucleoside as major anomer. Subsequent desilylation and cyclopropane ring opening of these tricyclic intermediates afforded the unsaturated 6’F-bc^4,3^ nucleosides. The successful incorporation of the corresponding phosphoramidite building blocks into oligonucleotides was achieved with *tert*-butyl hydroperoxide as oxidation agent. Thermal melting experiments of the modified duplexes disclosed a destabilizing effect versus DNA and RNA complements, but with a lesser degree of destabilization versus complementary DNA (Δ*T*_m_/mod = −1.5 to −3.7 °C). Molecular dynamics simulation on the nucleoside and oligonucleotide level revealed the preference of the C1’-*exo*/C2’-*endo* alignment of the furanose ring. Moreover, the simulation of duplexes with complementary RNA disclosed a DNA/RNA-type duplex structure suggesting that this modification might be a substrate for RNase H.

## Introduction

**Figure 1:**
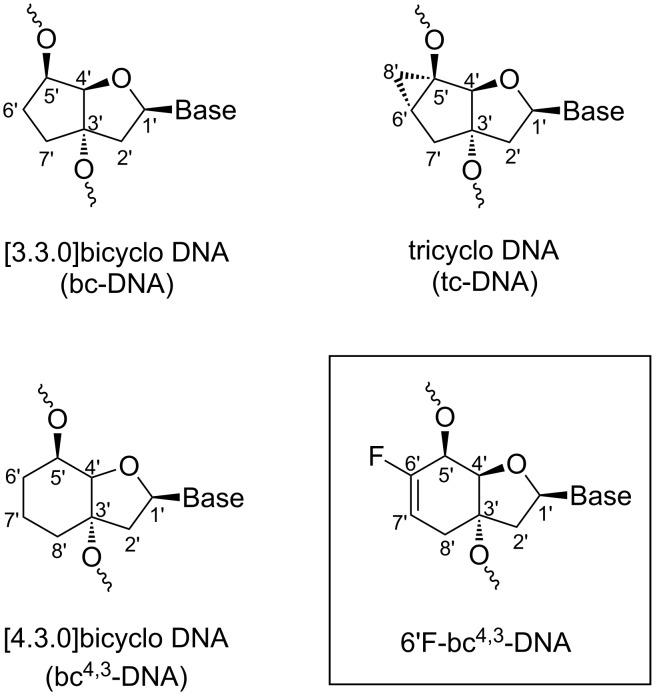
Chemical structure of selected nucleic acid analogs.

A powerful strategy for the treatment of various disorders like cancer, viral and inherited diseases is the use of therapeutic antisense oligonucleotides (AONs) [1–4]. These short, synthetic fragments bind through Watson–Crick base pairing to cellular RNA, thus modulating or silencing the gene expression through various mechanisms [5–7]. One mode of action is the recruitment of the endonuclease RNase H1 which selectively cleaves the RNA strand of a DNA/RNA hybrid duplex [8]. To activate this process, fully modified DNA-like oligonucleotides (ONs) or gapmer AONs are used [9–10]. However, the widespread use of oligonucleotide-based therapeutics is limited by several factors amongst which the cellular delivery, the biostability, the affinity and specificity for target RNA sequences are crucial [11]. A powerful strategy to increase the affinity for complementary RNA and to improve the ON biostability is the conformational restriction of the flexible backbone by additional ring systems and bridges [12]. Prominent members of this class of conformationally restricted nucleic acids are locked nucleic acids (LNAs) [13–15], hexitol nucleic acids (HNAs) [16–17], cyclohexenyl nucleic acids (CeNAs) [18–19], tricyclo-DNAs (tc-DNAs, Figure 1) [20–22], and [4.3.0]bicyclo-DNAs (bc^4,3^-DNAs, Figure 1) [23].

Another strategy to overcome some of the limitations deals with the insertion of one or several fluorine atom(s) in the sugar moiety of the nucleic acid analog. The polar hydrophobic nature [24] of the fluorine atom can positively alter the furanose conformation, basicity and/or polarizability of the modified nucleotide, and therefore influences the metabolic stability, the membrane permeability, the RNA- and protein-binding affinity of the AON [25–29]. Over the last almost two decades, fluorinated oligonucleotide analogs like 2’-deoxy-2’-fluoro-RNA (F-RNA) [26,30–31], 2’-deoxy-2’-fluoroarabino nucleic acid (F-ANA) [32–34], 3’-hexitol nucleic acids (FHNA and Ara-FHNA) [35], 2’-fluorocyclohexenyl nucleic acid (F-CeNA) [36], and other modifications [37–41] were evaluated for their antisense properties. In this context, our research group has systematically analyzed the effect of the fluorine atom on the bc-DNA and the tc-DNA scaffold. 6’-β-Fluorination of [3.3.0]bicyclo-DNA led to a pseudohydrogen bond interaction between the fluorine atom and the C(6) hydrogen atom of the thymine base, fixing the torsion angle χ. As a consequence, an increased affinity to complementary RNA targets was observed [42]. In the case of tc-DNA, C(6’) fluorination resulted in a similar affinity to RNA as standard tc-DNA suggesting no contribution of a fluorine pseudohydrogen bond as in bc-DNA [43]. Also a similar affinity to complementary DNA or RNA than the non-fluorinated tc-DNA was observed in fully modified 2’F-tc-ANA sequences [44]. In contrast, a significant stabilisation with RNA targets resulted in the case of the 2’F-tc-RNA modification due to conformational control of the furanose conformation [45]. To further widen the scope of fluorinated nucleic acid analogs as building blocks for therapeutic oligonucleotides, we investigated the bc^4,3^-DNA as scaffold for the modification. The idea was to place the fluorine atom next to the internucleosidic linkage. Furthermore, an additional double bond in the cyclohexane ring was expected to rigidify the carbocyclic unit and possibly positively impact the duplex stability. Here we report on the synthesis of the two 6’F-bc^4,3^ pyrimidine analogs with the base T and C, their incorporation into DNA, their biophysical properties, as well as a structural analysis by molecular dynamics simulations of hybrid DNA and RNA duplexes.

## Results and Discussion

### Synthesis of the phosphoramidite building blocks

**Scheme 1:**
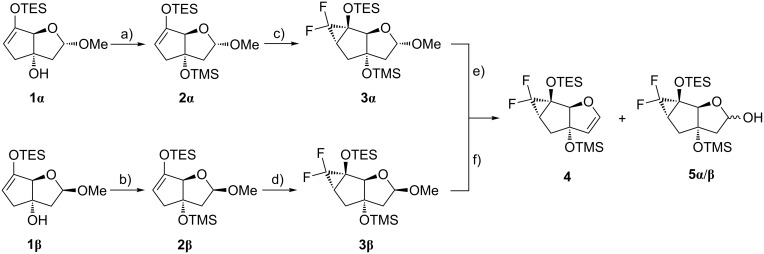
Synthesis of the *gem*-difluorinated glycal **4** from the silyl enol ethers **1α**/**β**. Reagents and conditions: a) BSA, DCM, rt, 17 h, 86%; b) BSA, DCM, rt, 18 h, 88%; c) TMSCF_3_, NaI, THF, 70 °C, 2 h, 71%; d) TMSCF_3_, NaI, THF, 70 °C, 4 h, 75%; e) TMSOTf, 2,6-lutidine, DCM, 0 °C to rt, 2 h, 41% (**4**), 39% (**5α**/**β**); f) TMSOTf, 2,6-lutidine, DCM, 0 °C to rt, 7 h, 58% (**4**), 29% (**5α**/**β**).

Our strategy for the construction of the two phosphoramidite building blocks **10** and **16** envisaged as a key step the formation of a [4.3.0]bicyclic fluoroenone from a tricyclic siloxydifluorocyclopropane through a ring enlargement via selective cyclopropane ring opening [46–49]. Consequently, the synthesis started from the previously described bicyclic silyl enol ethers **1α**/**β** (Scheme 1) [50–51]. The two anomers of **1** were individually transformed into the trimethylsilyl (TMS)-protected sugars **2α**/**β** by adapting and improving the already existing protocol [50]. The sugars **2α**/**β** were then individually treated with the Ruppert–Prakash reagent (TMSCF_3_) as difluorocarbene precursor and sodium iodide as initiator [52], furnishing the *exo*-tricyclic sugars **3α**/**β** as major isomers. The closer evaluation of this reaction revealed that the type of silyl enol ether drastically influenced the yield of the corresponding siloxydifluorocyclopropane. Whereas the TMS enol ethers were not suitable for the reaction due to instability of the silyl group, the *tert*-butyldimethylsilyl (TBDMS) enol ethers were poorly reactive most likely due to the hindrance of the difluorocarbene attack on the double bond. The stereochemistry around the cyclopropane ring (*endo* vs *exo*) could be assessed by the characteristic coupling pattern between the fluorine atom and the H-C(1) or C(7) in the *endo*-tricyclic sugars in the corresponding ^1^H and ^13^C NMR spectra (Supporting Information File 1).

**Scheme 2:**
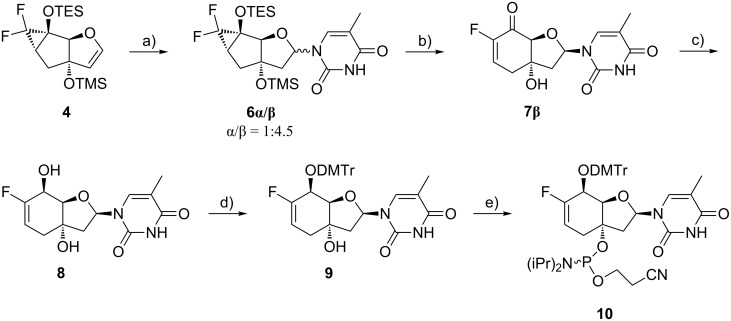
Synthesis of the thymidine phosphoramidite building block **10**. Reagents and conditions: a) i) thymine, BSA, NIS, DCM, 0 °C to rt, 4.5 h; ii) Bu_3_SnH, AIBN, toluene, 90 °C, 30 min, 70%; b) HF-pyridine, DCM/pyridine 5:1, 0 °C to rt, 1.5 h, 71%; c) CeCl_3_·7H_2_O, NaBH_4_, MeOH, 0 °C, 1 h, 92%; d) DMTr-Cl, pyridine, rt, 3 d, 76%; d) CEP-Cl, DIPEA, THF, rt, 4 h, 62%.

The plan for the pyrimidine nucleoside synthesis comprised the use of the meanwhile well-established β-selective *N*-iodosuccinimide (NIS)-mediated addition of a persilylated nucleobase to a tricyclic glycal [43,45,53–54]. Therefore, the *gem*-difluorinated tricyclic sugars **3α**/**β** were individually reacted with trimethylsilyl trifluoromethanesulfonate (TMSOTf) in order to produce the corresponding glycal **4**. Surprisingly, apart from the desired glycal **4** its hydrolysis products **5α**/**β** were produced as main side products. In the case of the α-tricyclic sugar **3α** the ratio of products **4** to **5α**/**β** could be influenced by the reaction time. A shorter reaction time furnished the tricyclic alcohols **5α**/**β** as major product, while prolongation of the reaction time produced the glycal **4** as main component (Table S1, Supporting Information File 1). Treatment of the glycal **4** with persilylated thymine in the presence of NIS (Scheme 2), followed by radical reduction of the iodide intermediate with tributyltin hydride (Bu_3_SnH) generated an anomeric mixture of nucleoside **6α**/**β** with the β-anomer as major component (α/β ratio = 1:4.5 according to ^1^H NMR). The inseparable anomers of nucleoside **6α**/**β** were subjected to the next reaction step, where the simultaneous desilylation and cyclopropane ring opening to the bicyclic fluoroenone **7** occurred. HF-pyridine smoothly facilitated this conversion. At this stage the two anomers of fluoroenone **7** were separable. The configurational assignment of the nucleobase was conducted by ^1^H,^1^H-ROESY experiments (Supporting Information File 1). The β-anomer **7β** then was subjected to Luche reduction [55–56] producing selectively the desired *S*-configuration at the C(5’) position due to hydride delivery from the less hindered *exo*-side of the carbonyl group. The relative configuration at C(5’) could be assigned by ^1^H,^1^H-ROESY experiments (Supporting Information File 1). Tritylation of allylic alcohol **8** with 4,4-dimethoxytrityl chloride (DMTr-Cl) afforded intermediate **9** which was subsequently phosphitylated with 2-cyanoethyl *N*,*N*-diisopropylchlorophosphoramidite (CEP-Cl) furnishing thymidine phosphoramidite **10**.

**Scheme 3:**
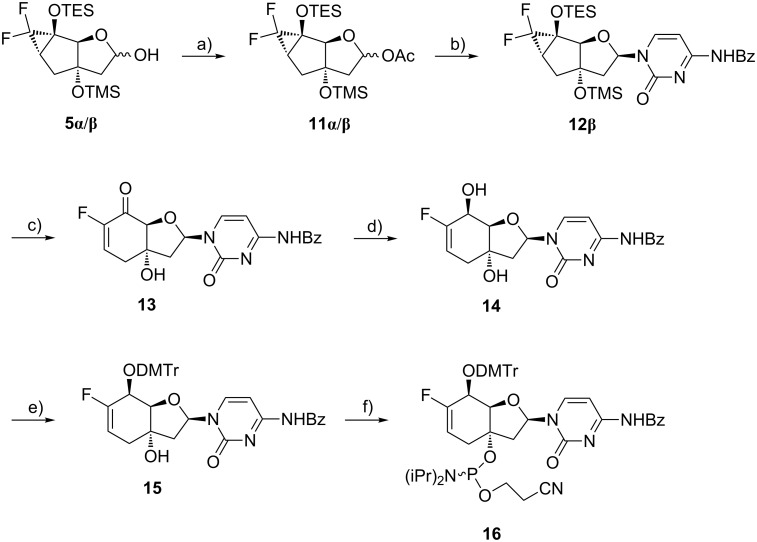
Synthesis of the cytidine phosphoramidite building block **16**. Reagents and conditions: a) Ac_2_O, pyridine, 0 °C to rt, 17 h, 87%; b) *N*-benzoylcytosine, BSA, TMSOTf, ACN, 0 °C to rt, 3.5 h, 41%; c) HF-pyridine, DCM/pyridine 5:1, 0 °C, 15 min, 91%; d) i) CeCl_3_·7H_2_O, NaBH_4_, MeOH, −78 °C, 20 min; ii) Bz_2_O, DMF, rt, 7 h, 94%; e) DMTr-OTf, DCM/pyridine 1:2, rt, 19.5 h, 44%; f) CEP-Cl, DIPEA, THF, rt, 75 min, 43%.

Since significant amounts of the alcohols **5α**/**β** were obtained, it was decided to redirect our initial synthetic plan for the cytidine phosphoramidite. Hence, the sugars **5α**/**β** were first acetylated yielding the intermediate **11α**/**β** (Scheme 3), which is a standard glycosyl donor for nucleoside synthesis. The nucleosidation was carried out by applying classical Vorbrüggen conditions [57] on the sugars **11α**/**β**, yielding the β-nucleoside **12β** as major anomer. The α/β-ratio of 1:1.5 was acceptable and the configuration at the C(1’) was assigned by ^1^H,^1^H-ROESY experiments (Supporting Information File 1). The *gem*-difluorinated tricyclic nucleoside **12β** was then converted into the bicyclic fluoroenone **13** via desilylation and ring-enlargement by short exposure to HF-pyridine. During the following Luche reduction of derivative **13** the benzoyl protecting group of the nucleobase was partially removed. As a consequence an additional benzoylation step was needed to obtain the allylic alcohol **14** in high yields. Verification of the configuration at C(5’) was again accomplished by ^1^H,^1^H-ROESY experiments (Supporting Information File 1). Tritylation of the nucleoside **14** with in situ-prepared 4,4-dimethoxytrityl methanesulfonate (DMTr-OTf) [58–59] provided the protected derivative **15** which was phosphitylated yielding the cytidine phosphoramidite **16**.

### Synthesis of oligonucleotides

**Figure 2:**
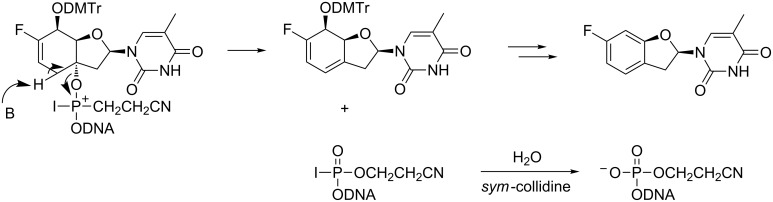
Proposed mechanism for the formation of the 5’-phosphorylated fragments during the oxidation step in the synthesis of **ON1** and **ON2**.

A series of oligonucleotides containing single or multiple incorporations of the thymidine or cytidine building blocks **10** and **16** were synthesized to study the pairing properties of the new modification with complementary DNA and RNA. At the beginning, the synthesis of the oligonucleotides was conducted using standard automated phosphoramidite chemistry (for details see the experimental part in Supporting Information File 1). However, in the synthesis of **ON1** and **ON2** the yield dropped to approximately 40% after the incorporation of the modified unit. The analysis of the crude product by LC–MS after cleavage from the solid support and deprotection, revealed the presence of 5’-phosphorylated fragments originating from a 3’-cleavage of the modification. We propose, that these fragments were formed through an E2 elimination during the oxidation step, most likely on the iodophosphonium ion level (Figure 2) [60]. Based on this hypothesis, we changed the oxidation agent from iodine to *tert*-butyl hydroperoxide (TBHP), which previously has been successfully applied for the synthesis of iso-tricyclo-T (iso-tc-T) or bc^en^-T containing oligonucleotides [61]. Indeed, under these conditions high coupling yields (>98%) of **ON3**–**7** were obtained and the absence of the 5’-phosphorylated fragment was noticed.

### Pairing properties with complementary DNA and RNA

To evaluate the effect of the unsaturated 6’F-bc^4,3^ modification on the thermal duplex stability we conducted UV-melting experiments of the modified oligonucleotides with DNA and RNA (Table 1). These studies showed that **ON1**–**4** bearing the modified thymidine unit exhibited a duplex destabilization when paired to DNA (Δ*T*_m_/mod = −1.5 to −3.7 °C). The *T*_m_ depression was more pronounced for multiple (Δ*T*_m_/mod ≈ −3.5 °C) than for single (Δ*T*_m_/mod ≈ −2.0 °C) inclusions. When the same four oligonucleotides were hybridized to RNA the duplex stability further decreased (Δ*T*_m_/mod ≈ −4.0 °C). However, the number of modified units seemed not to have an influence on the *T*_m_ value. The three oligonucleotides **ON5**–**7** bearing the cytidine modification also expressed a destabilizing pattern with both complements, but to a lesser amount than in the thymidine series. Again, the *T*_m_ depression was higher with complementary RNA, with pronounced sequence effects in the case of single inclusions. The less destabilizing behaviour of the 6’F-bc^4,3^-modified oligonucleotides versus complementary DNA suggested that this modification more presents a DNA than a RNA mimic. The better tolerance of the cytidine modification in both duplex types could be assigned to the nature of the nucleobase and was also observed in the case of the 7’,5’-bc-DNA [62].

**Table 1:** *T*_m_ and Δ*T*_m_/mod data from UV-melting curves (260 nm) of **ON1**–**7** in duplexes with complementary DNA and RNA.

Entry	Sequence^a^	*T*_m_ [°C] vs DNA	Δ*T*_m_/mod [°C]	*T*_m_ [°C] vs RNA	Δ*T*_m_/mod [°C]

**ON1**	5’-d(GGA TGT TCt CGA)-3’	46.0	−2.7	46.0	−4.0
**ON2**	5’-d(GGA tGT TCT CGA)-3’	47.2	−1.5	47.6	−2.4
**ON3**	5’-d(GGA TGt tCT CGA)-3’	41.3	−3.7	42.0	−4.0
**ON4**	5’-d(GCA ttt ttA CCG)-3’	30.3	−3.4	22.4	−4.4
**ON5**	5’-d(GGA TGT TcT CGA)-3’	47.1	−1.6	46.0	−4.0
**ON6**	5’-d(GGA TGT TCT cGA)-3’	46.8	−1.9	48.3	−1.7
**ON7**	5’-d(GGA TGT TcT cGA)-3’	44.1	−2.3	44.0	−3.0

^a^Lowercase letters: modified nucleotides, capital letters: natural DNA. Total strand conc. 2 μM in 10 mM NaH_2_PO_4_, 150 mM NaCl, pH 7.0. Reference *T*_m_ values: DNA1/DNA = 48.7 °C, DNA1/RNA = 50.0 °C, DNA2/DNA = 47.4 °C, DNA2/RNA = 44.4 °C; DNA1 = 5’-d(GGA TGT TCT CGA)-3’, DNA2 = 5’-d(GCA TTT TTA CCG)-3’.

The determination of the base pair selectivity of the 6’F-bc^4,3^ modification was carried out by UV-melting experiments of **ON1** with complementary DNA where the mismatched base was inserted at the opposing site of the modification (Table S2, Supporting Information File 1). All three possible mismatches were evaluated. As expected, in all cases the *T*_m_ value was significantly lowered, with the GT-Wobble pair having the least destabilizing effect (−8.5 °C). The *T*_m_ depression of the GT-Wobble pair and the CT-mismatch (−11.0 °C) was in the same range than for the natural duplex. However, a lager *T*_m_ discrimination was found in the TT-mismatch (−14.1 °C) as in the natural system (−9.7 °C). Taking together, these data suggest that classical Watson–Crick base pairing also occurs with this modification.

The thermodynamic parameters of duplex formation of **ON4** and the corresponding natural sequence versus both complements were extracted from their melting curves by a known curve fitting methodology (Table S3, Supporting Information File 1) [63]. The comparison of the modified with the natural duplexes disclosed an entropic stabilization (ΔΔ*S* = +28.9 and +38.0 cal·mol^−1^·K^−1^) and an enthalpic destabilization (ΔΔ*H* = +13.8 and +17.0 kcal·mol^−1^) for both the DNA and RNA complement. This pattern was observed along the whole bc-DNA series and was attributed to the conformational restriction of the sugar [62,64]. The Gibbs free energy of duplex formation corresponded well with the observed *T*_m_ values.

### CD spectroscopy

Circular dichroism of **ON1**–**7** paired with DNA or RNA was recorded to further analyze their helical conformation and to compare it with that of the corresponding natural duplexes (Figure S1, Supporting Information File 1). All seven modified oligonucleotides exhibited a B-type pattern when paired to DNA, indicating B-form helices. All modified oligonucleotides duplexed to RNA disclosed a similar pattern than the natural hybrid structure, giving evidence of mixed A/B-type helices.

### Molecular modeling

**Figure 3:**
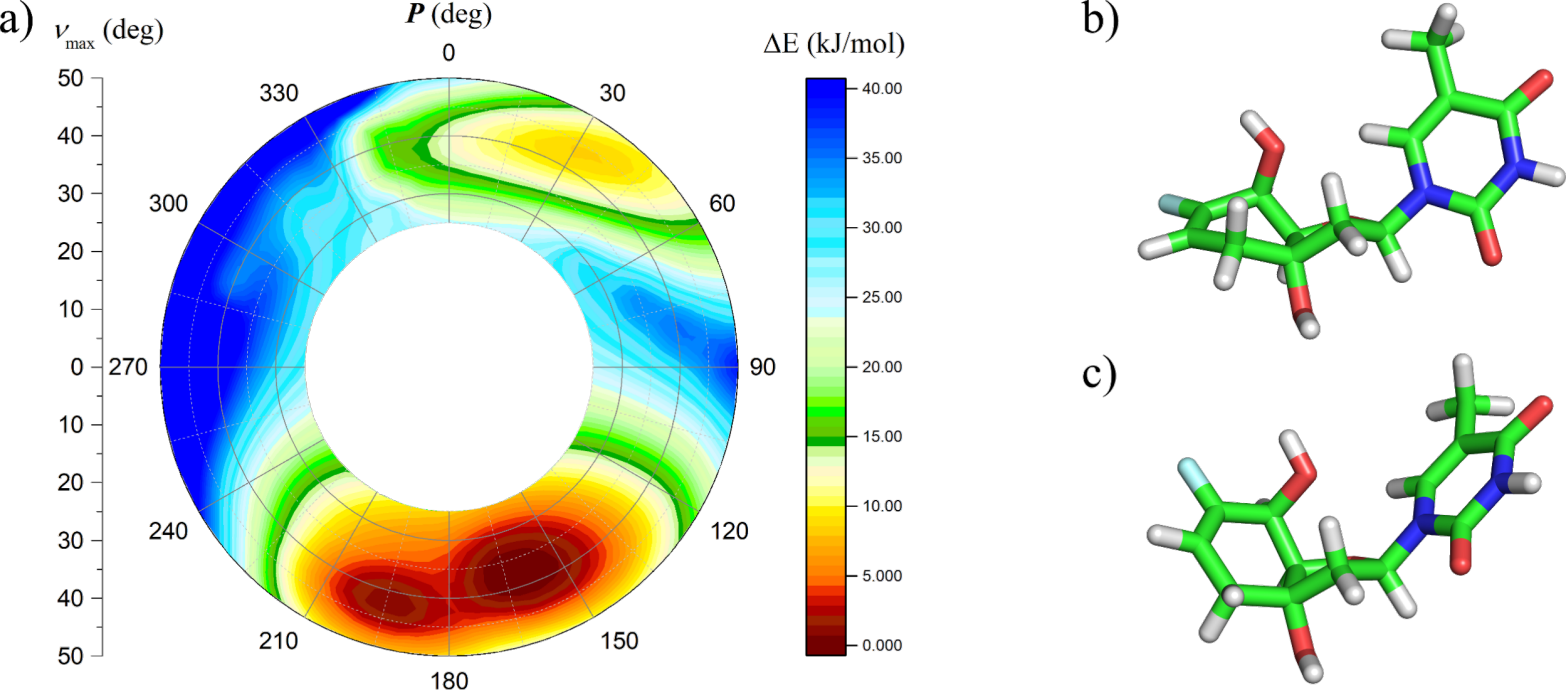
a) Potential energy profile versus pseudorotation phase angle of nucleoside **8** and its two minimal energy conformers: b) C2’-*endo* and c) C3’-*exo*.

To gain more information on the structural features of the 6’F-bc^4,3^ modification, we performed molecular dynamics simulations of the modified duplexes. We first calculated the potential energy profile versus pseudorotation phase angle of nucleoside **8** using quantum mechanical methods. The calculations were performed in vacuum with the Gaussian 09 software package [65] utilizing the second order Møller–Plesset perturbation theory (MP2) and the 6-311G* basis set. The energy profile of nucleoside **8** was obtained through a stepwise variation of the pseudorotation phase angle *P* at the range of the maximum puckering amplitude *ν*_max_ and was visualized in the pseudorotation wheel (Figure 3a). The two low energy regions appeared in the Southern hemisphere. The lowest energy conformer was associated with the furanose unit in a C2’-*endo* orientation and the six-membered ring in a twist-boat conformation (Figure 3b). Approximately 1 kJ/mol higher in energy was the second conformer where the furanose unit adopted a C3’-*exo* arrangement and the cyclohexene unit a half-chair conformation (Figure 3c). The C(5’) hydroxy group adopted in both conformers a pseudoaxial position. Consequently, the torsion angle γ was aligned in a *+sc* arrangement (C2’-*endo* conformer: 64°; C3’-*exo* conformer: 83°). The distance between the fluorine atom and the C(5’) oxygen was 3.3 Å in the C2’-*endo* conformer and 2.9 Å in the C3’-*exo* conformer.

These two conformers were then used to calculate the atomic charges of the corresponding nucleosides using the R.E.D. III.5 tools package [66]. The obtained parameters were added to the Amber94 force field [67] which besides the GROMACS 5.0.6 simulation package [68] was utilized for the molecular dynamics simulations. The duplexes investigated in the simulation encompassed: a unmodified DNA strand, **ON1**, **ON4** and a fully modified 6’F-bc^4,3^-DNA strand duplexed to complementary DNA and RNA as well as a 6’F-bc^4,3^-DNA homo-duplex (for details on the simulation see the experimental part in Supporting Information File 1).

**Figure 4:**
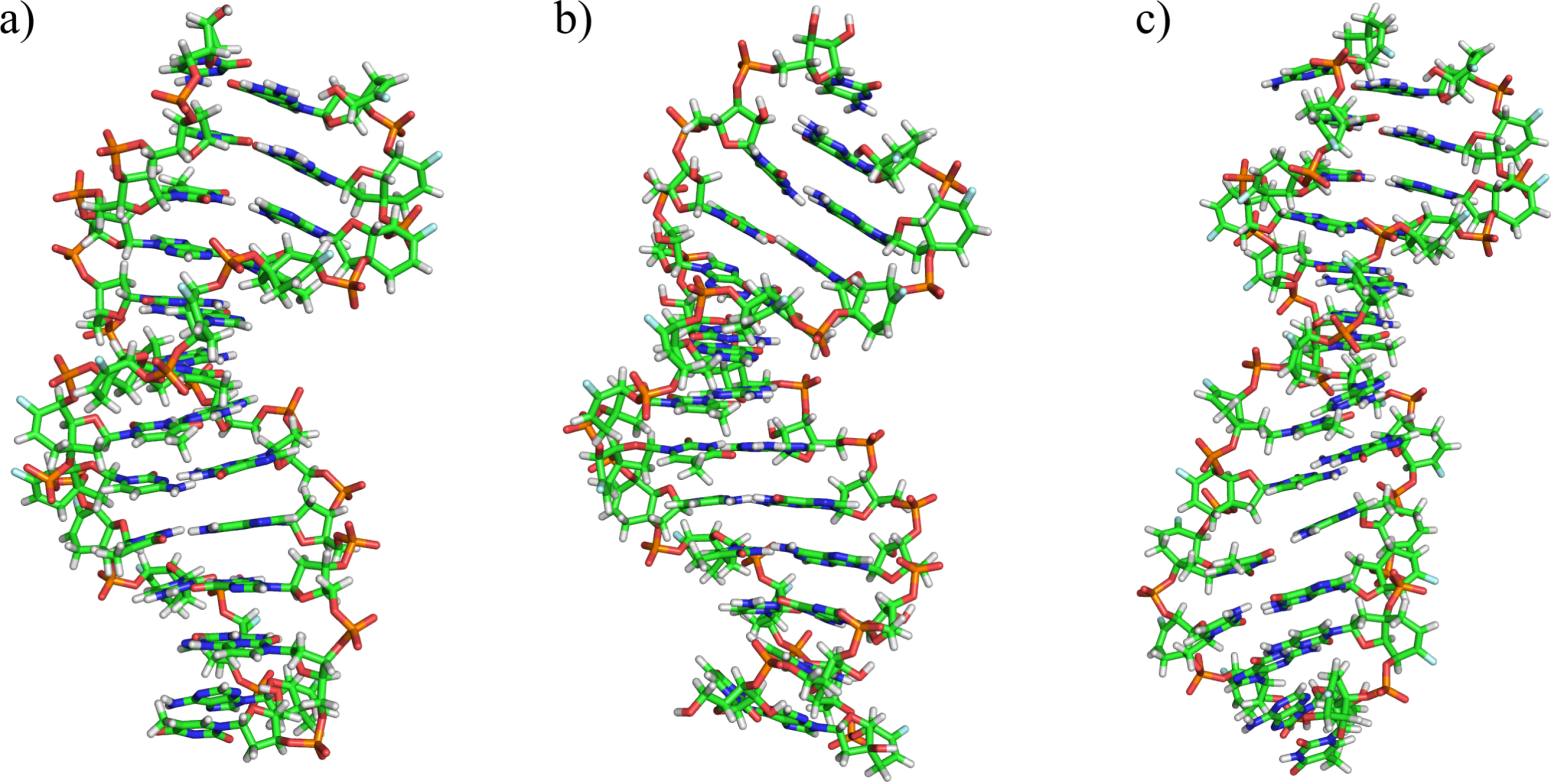
Average structures of the a) 6’F-bc^4,3^-DNA/DNA, b) 6’F-bc^4,3^-DNA/RNA, and c) 6’F-bc^4,3^-DNA/6’F-bc^4,3^-DNA duplexes obtained from the last nanosecond of the simulation by firstly extracting a frame each 50 ps and secondly by doing an averaging of them.

**Figure 5:**
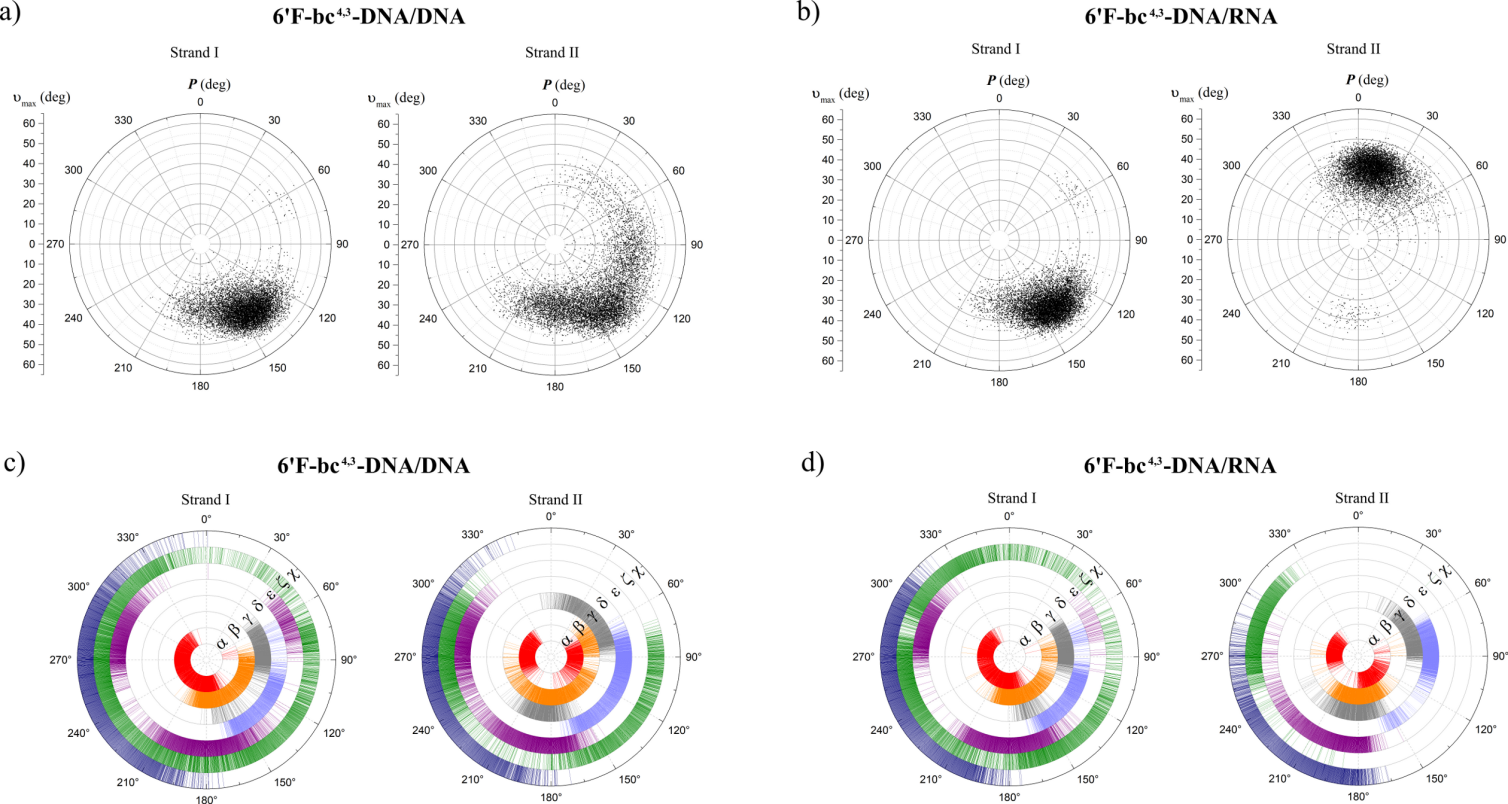
Preferred sugar pucker of a) 6’F-bc^4,3^-DNA/DNA, and b) 6’F-bc^4,3^-DNA/RNA duplexes and torsion angles of c) 6’F-bc^4,3^-DNA/DNA, and d) 6’F-bc^4,3^-DNA/RNA duplexes extracted from a 100 ns molecular dynamics trajectory.

The duplex of the fully modified 6’F-bc^4,3^-DNA strand with DNA still featured a B-type helix (Figure 4a) whereas the 6’F-bc^4,3^-DNA/RNA duplex maintained an A-form (Figure 4b). Interestingly, the fully modified 6’F-bc^4,3^-DNA strand exhibited almost identical backbone angles and sugar conformation regardless if paired to DNA or RNA. The preferred sugar arrangement was found in a narrow range in the Southern area of the pseudorotation wheel (C1’-*exo*, C2’-*endo*, Figure 5a and b), indicating that this modification is a DNA mimic. This finding is in agreement with the observed *T*_m_ values and also reflects the entropical stabilization of the duplex structure. The cyclohexene ring of the modified unit adopted either a twist-boat or a boat alignment in the fully modified strand of both duplex types. Consequently, the fluorine atom was arranged in a way that the repulsive electrostatic interactions with the C(5’) oxygen were minimized. The analysis of the backbone torsion angles revealed that the fused ring system affected all backbone torsion angles (Figure 5c and d). Specifically, the angle α adopted values in the *+ap* to *−sc* range which was in contrast to the canonical parameters (DNA: *±sc*, *−ac*; RNA *+ac*, *+ap*, *−sc*). The angle β was found in the *+ac* or *anti* orientation, most likely due to either the boat or the twist-boat conformation of the cyclohexene ring. Furthermore, the angle γ was constrained to a *+sc* arrangement as also found in canonical A- or B-type helices. The torsion angle ε exhibited values in the *±sc* and *anti* range, whereas the angle ζ adopts all values between 0–360°. The reason for the flexibility of the angle ζ might lie in its compensatory nature to balance the constrained backbone angles that lay within the carbocyclic system.

The DNA or RNA strand in these hybrid duplexes displayed the same structural preference as in the natural reference structures (Figures S2 and S3, Supporting Information File 1). The evaluation of the base pair body parameters of the 6’F-bc^4,3^-DNA strand hybridized to DNA or RNA revealed the expected Watson–Crick base pairing between the two strands and the characteristic parameters of a B- or A-type helix, respectively (Figures S5–S7, Supporting Information File 1) [69]. Furthermore, the examination of the minor groove distances [70] disclosed for the 6’F-bc^4,3^-DNA/RNA duplex a flexibility switching between values of an A- and B-helix (Figure S8, Supporting Information File 1). This variation of the minor groove distance is thought to play a crucial role for RNase H activation [9,71].

The structure displayed by the fully modified 6’F-bc^4,3^-DNA homo-duplex was neither an A- nor B-type helix (Figure 4c). This structure featured a very variable minor groove (≈8 to 18 Å), an increased rise (≈3.4 Å), a positive slide (≈1.6 Å) and a positive roll (≈4.6 Å; Figures S6–S8, Supporting Information File 1). As a consequence of the latter the x-displacement (≈1.1 Å) was shifted towards a positive value. The sugar conformation in the two strands (Figure S4, Supporting Information File 1) was in the same range (C1’-*exo*, C2’-*endo*) as described above for the hybrid duplexes. The backbone torsion angles of the homo-duplex exhibited identical conformations in both strands (Figure S4, Supporting Information File 1). Some variations in the torsion angle ζ (*−sc* to *+ac*) and the glycosidic bond angle χ (200–360°) were observed compared to the fully modified hybrid duplexes.

The structural data of **ON1** and **ON4** containing either one or five consecutive modifications are shown in Figures S2, S3, and S8 in Supporting Information File 1.

## Conclusion

In this study, we presented the successful synthesis of the two 6’F-bc^4,3^ pyrimidine phosphoramidite building blocks **10** and **16** starting from a bicyclic silyl enol ether. The key step in the synthesis was the transformation of a *gem*-difluorinated tricyclic nucleoside into a ring-enlarged bicyclic fluoroenone by simultaneous desilylation and cyclopropane ring opening which proceeded in high yields. The two phosphoramidite building blocks were successfully incorporated into oligonucleotides by automated solid-phase DNA synthesis with *tert*-butyl hydroperoxide as the oxidation agent. The CD spectra of the 6’F-bc^4,3^-T or -C-modified oligonucleotides displayed a B-type helix when paired to DNA and an intermediate A/B form when the counter part was RNA.

The modified oligonucleotides exhibited a significant destabilization versus both complements, but with complementary DNA being less discriminating (Δ*T*_m_/mod = −1.5 to −3.7 °C) than complementary RNA. This finding indicates that the 6’F-bc^4,3^ modification is more a DNA mimic than an RNA mimic. In accordance with this were the results obtained from the molecular dynamics simulation of the duplexes where the sugar pucker preferably adopted a Southern conformation (C1’-*exo*, C2’-*endo*). Furthermore the simulations revealed a very rigid bicyclic sugar system with a diminished conformational adaptability of the cyclohexene unit. Mainly this rigidity in combination with the repulsive electrostatic interactions of the fluorine atom and the C(5’) oxygen seem to be responsible for the duplex destabilization. Nevertheless, the MD simulations pointed to a flexible minor groove for the modified oligonucleotides hybridized to RNA, indicating together with the preferred Southern conformation of the modified unit, that this modification might be a substrate for RNase H.

## Supporting Information

Additional tables and figures, the experimental part, as well as copies of the NMR spectra (^1^H, ^13^C, ^19^F, ^31^P) of the new compounds are given in the Supporting Information.

File 1 Additional data, experimental part, and NMR spectra.
