# Supplementary material for: 6’-Fluoro[4.3.0]bicyclo nucleic acid: synthesis, biophysical properties and molecular dynamics simulations
Source: Beilstein J Org Chem. 2018 Dec 20;14:3088–97. doi: 10.3762/bjoc.14.288 (PMC6317435; doi:10.3762/bjoc.14.288)
Supplement: File 1 — Additional data, experimental part, and NMR spectra. [file Beilstein_J_Org_Chem-14-3088-s001.pdf]

BEILSTEIN JOURNAL OF ORGANIC CHEMISTRY

# Supporting Information

for

# 6’-Fluoro[4.3.0]bicyclo nucleic acid: synthesis, biophysical properties and molecular dynamics simulations

Sibylle Frei, Andrei Istrate and Christian J. Leumann

*Beilstein J. Org. Chem.* **2018,** *14,* 3088–3097. doi:10.3762/bjoc.14.288

## Additional data, experimental part, and NMR spectra

License and Terms: This is a supporting information file under the terms of the Creative Commons Attribution License (http://creativecommons.org/licenses/by/4.0). Please note that the reuse, redistribution and reproduction in particular requires that the authors and source are credited.
The license is subject to the *Beilstein Journal of Organic Chemistry* terms and conditions: (https://www.beilstein-journals.org/bjoc)

**Table of Contents**


1. Formation of glycal **4** and alcohol **5α/β**………………………………………… S3

2. Mismatch discrimination…………………………………………………………… S3

3. Thermodynamic parameters of duplex formation……………………………….. S4

4. CD spectra of the modified duplexes……………………………………………... S4

5. Sugar pucker conformation and torsion angles of the modeled duplexes……. S5

6. Base pair body parameters of the modeled duplexes…………………………... S7

7. Minor groove distance of the modeled duplexes………………………………… S8

8. Experimental part…………………………………………………………………. S9

8.1 General…………………………………………………………………………… S9

8.2 UV-melting curves……………………………………………………………….... S9

8.3 Circular dichroism spectroscopy ………………………………………........ S10

8.4 Molecular modeling…………………………………………………………….. S10

8.5 Oligonucleotide synthesis and purification………………………………………S11

8.6 Numbering of the sugars and nucleosides…………………………………..... S14

8.7 Synthesis and characterization………………………………………………... S14

9. NMR spectra of the new synthesized compounds……………………………… S29

10. References……………………………………………………………………… S56

## 1. Formation of glycal 4 and alcohol 5α/β

**Table S1:** Time-dependent product formation in the TMSOTf-mediated elimination of the methoxy group starting from the sugar **3α**.

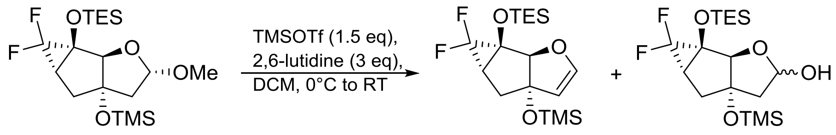


**3α** **4** **5α/β**

| Entry | Time [h] | Yield **4** [%] | Yield **5** [%] |
|---|---|---|---|
| 1 | 1.2 | 21 | 60 |
| 2 | 1.5 | 28 | 54 |
| 3 | 1.7 | 42 | 21 |
| 4 | 2.1 | 41 | 39 |
| 5 | 2.6 | 67 | 24 |

## 2. Mismatch discrimination

**Table S2:** $T_m$ data [°C] from UV-melting curves (260 nm) of **DNA1** and **ON1** in duplex with complementary mismatched DNA.

| Entry | Sequence[a] | X = A | X = T | X = G | X = C |
|---|---|---|---|---|---|
| **DNA1** | 5'-d(GGA TGT TCT CGA)-3' | 48.7 | 39.0 | 40.2 | 37.3 |
| **DNA** | 5'-d(TCG XGA ACA TCC)-3' | | (-9.7) | (-8.5) | (-11.4) |
| **ON1** | 5'-d(GGA TGT TCt CGA)-3' | 46.0 | 31.9 | 37.1 | 35.0 |
| **DNA** | 5'-d(TCG XGA ACA TCC)-3' | | (-14.1) | (-8.9) | (-11.0) |

[a]Lowercase letter: modified nucleotide, capital letters: natural DNA. Total strand conc. 2 μM in 10 mM $NaH_2PO_4$, 150 mM NaCl, pH 7.0.

## 3. Thermodynamic parameters of duplex formation

**Table S3:** Thermodynamic data of duplex formation for **DNA2** and **ON4** hybridized to complementary DNA and RNA.

| Entry | vs[a] | $\Delta H$ [kcal/mol] | $\Delta S$ [cal/mol*K] | $\Delta G_{25°C}$ [kcal/mol] |
|---|---|---|---|---|
| **DNA2** | DNA | -97.3 ± 0.8 | -270.3 ± 2.4 | -16.8 ± 0.1 |
| **ON4** | | -83.5 ± 0.4 | -241.4 ± 1.3 | -11.5 ± 0.1 |
| **DNA2** | RNA | -88.4 ± 1.0 | -246.9 ± 3.1 | -14.8 ± 0.1 |
| **ON4** | | -71.4 ± 1.1 | -208.9 ± 3.4 | -9.1 ± 0.1 |

[a]Total strand conc. 2 μM in 10 mM $NaH_2PO_4$, 1 M NaCl, pH 7.0.

## 4. CD spectra of the modified duplexes

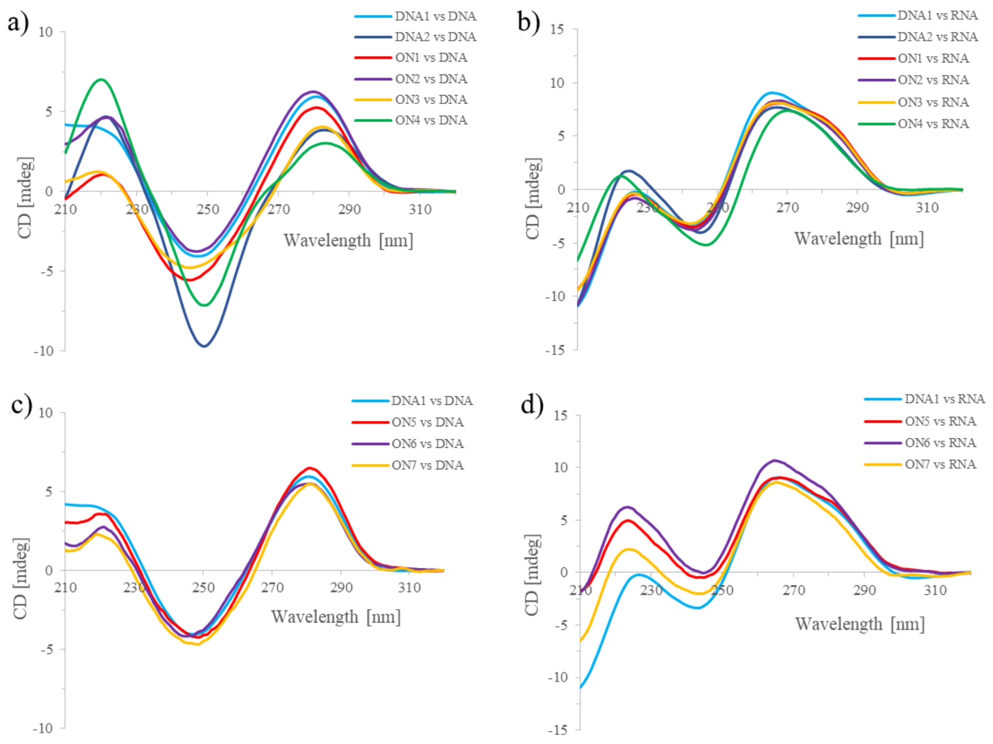


**Figure S1:** CD spectra of the **ON1** - **7**. ONs containing the 6'F-bc$^{4,3}$-T residues in the DNA backbone with complementary a) DNA, and b) RNA. ONs containing the 6'F-bc$^{4,3}$-C residues in the DNA backbone with complementary c) DNA, and d) RNA. Total strand conc. 2 μM in 10 mM $NaH_2PO_4$, 150 mM NaCl, pH 7.0.

## 5. Sugar pucker conformation and torsion angles of the modeled duplexes

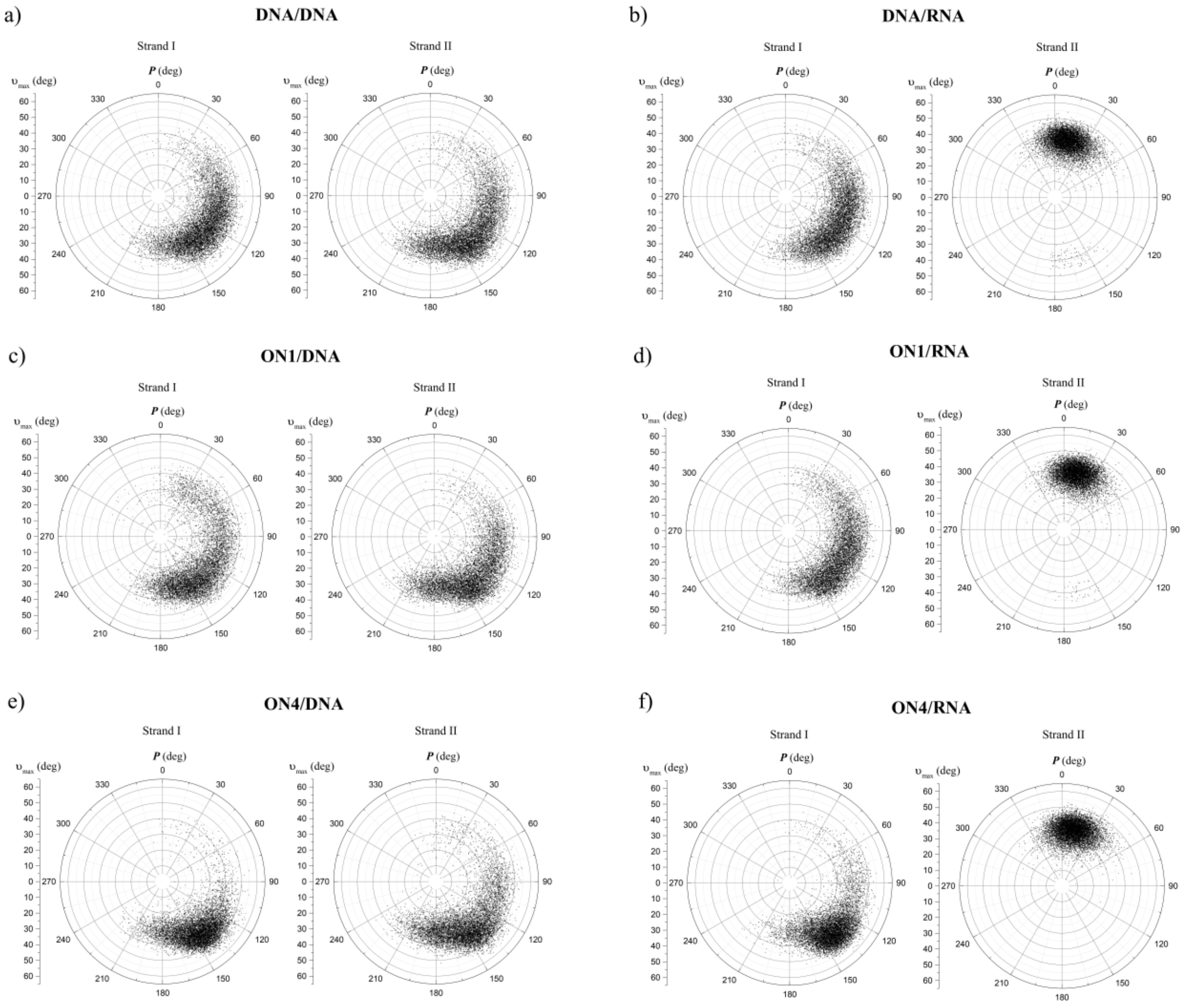


**Figure S2:** Preferred sugar pucker of a) DNA/DNA, b) DNA/RNA, c) **ON1**/DNA, d) **ON1**/RNA, e) **ON4**/DNA, and f) **ON4**/RNA duplexes extracted from a 100 ns molecular dynamics trajectory.

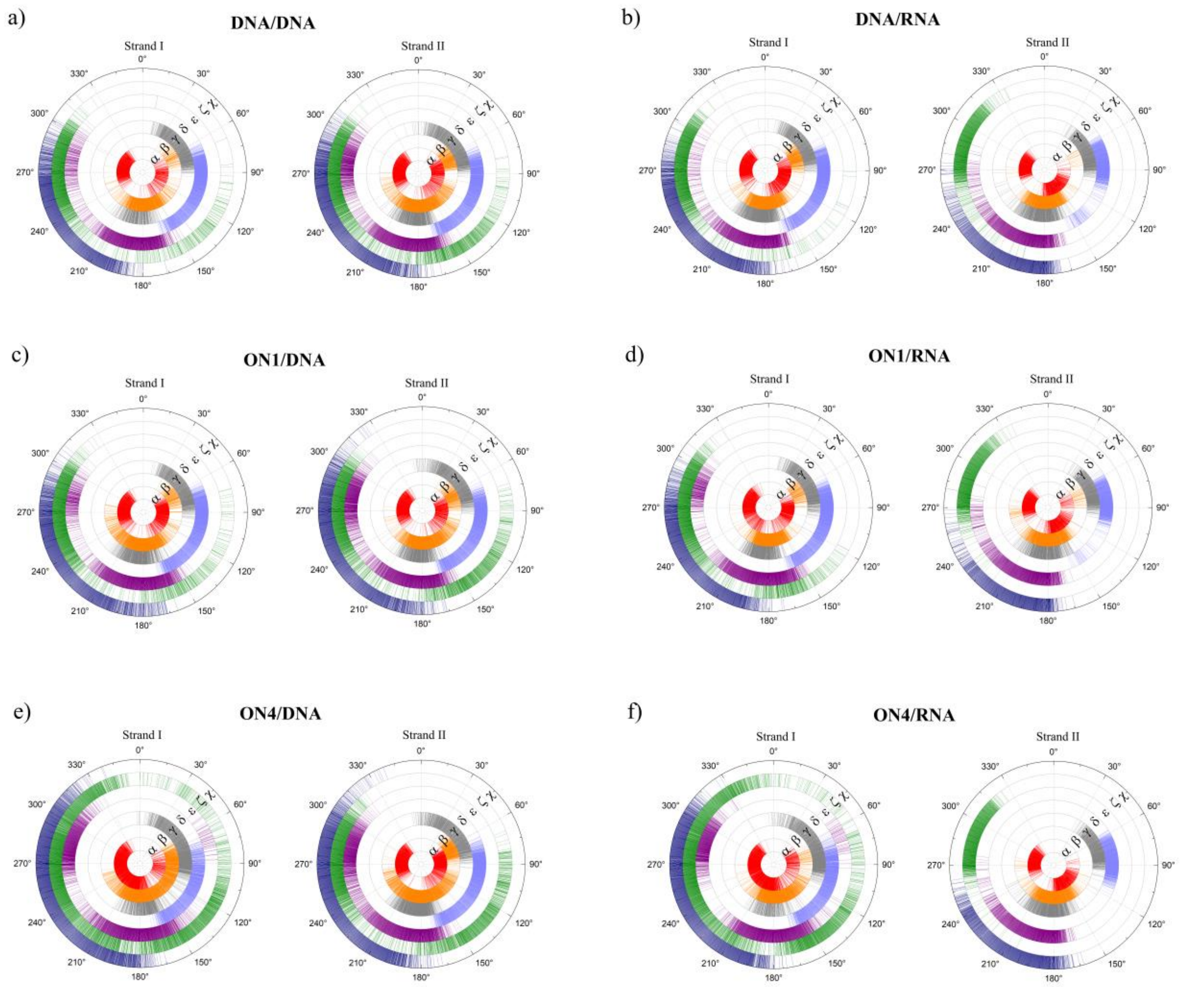


**Figure S3:** Torsion angles of a) DNA/DNA, b) DNA/RNA, c) **ON1**/DNA, d) **ON1**/RNA, e) **ON4**/DNA, and f) **ON4**/RNA duplexes extracted from a 100 ns molecular dynamics trajectory.

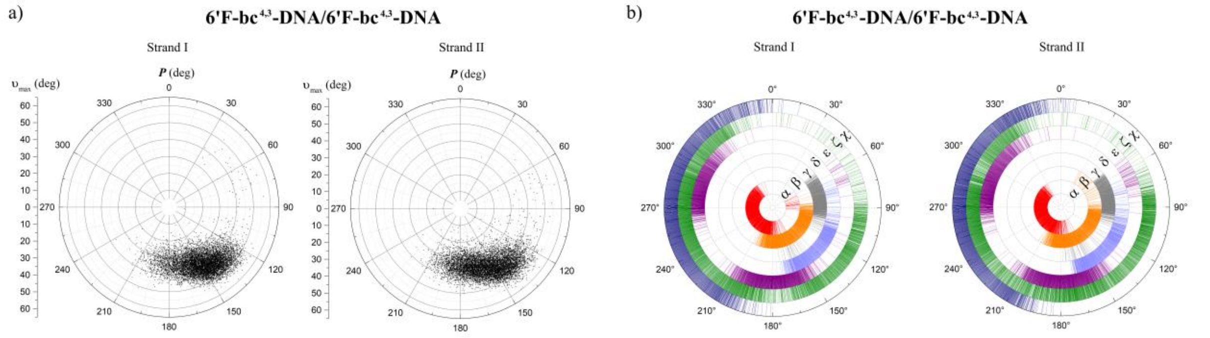


**Figure S4:** a) Preferred sugar pucker conformation and b) torsion angles of the 6’F-bc$^{4,3}$-DNA homo-duplex extracted from a 100 ns molecular dynamics trajectory.

## 6. Base pair body parameters of the modeled duplexes

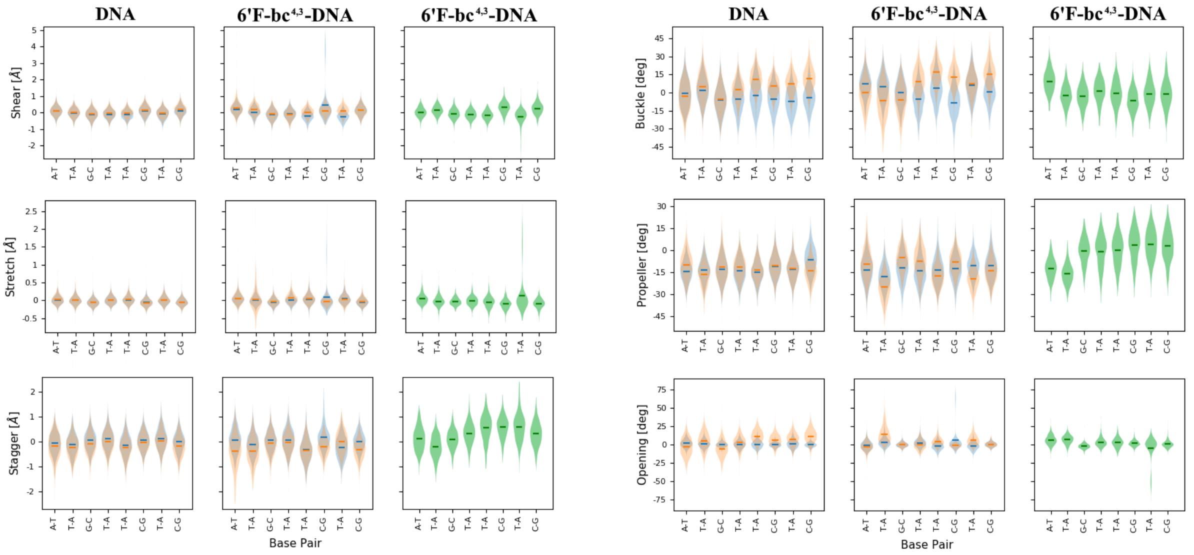


**Figure S5:** Base pair parameters of DNA and 6'F-bc$^{4,3}$-DNA hybridized to DNA (blue) or RNA (red) and of the 6'F-bc$^{4,3}$-DNA homo-duplex (green) extracted from a 100 ns molecular dynamics trajectory.

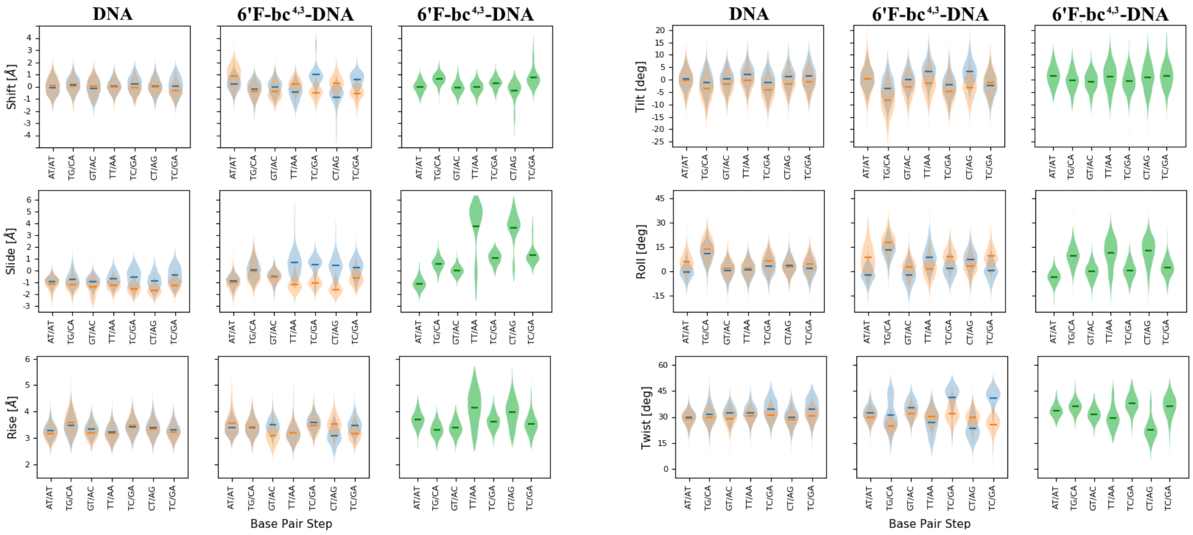


**Figure S6:** Base pair step parameters of DNA and 6'F-bc$^{4,3}$-DNA hybridized to DNA (blue) or RNA (red) and of the 6'F-bc$^{4,3}$-DNA homo-duplex (green) extracted from a 100 ns molecular dynamics trajectory.

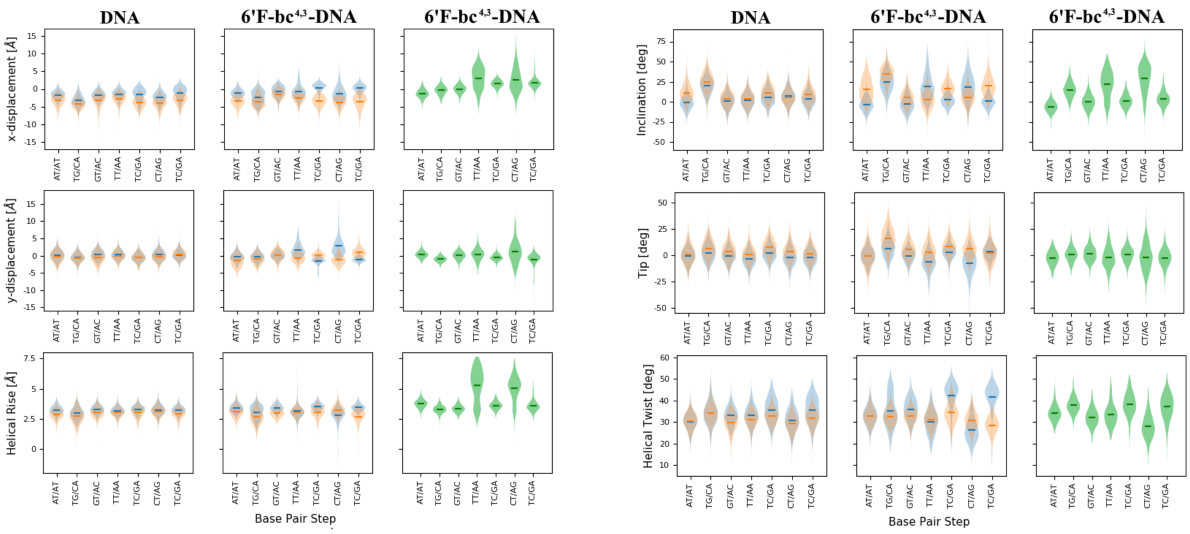


**Figure S7:** Helical base pair step parameters of DNA and 6’F-bc$^{4,3}$-DNA hybridized to DNA (blue) or RNA (red) and of the 6’F-bc$^{4,3}$-DNA homo-duplex (green) extracted from a 100 ns molecular dynamics trajectory.

## 7. Minor groove distance of the modeled duplexes

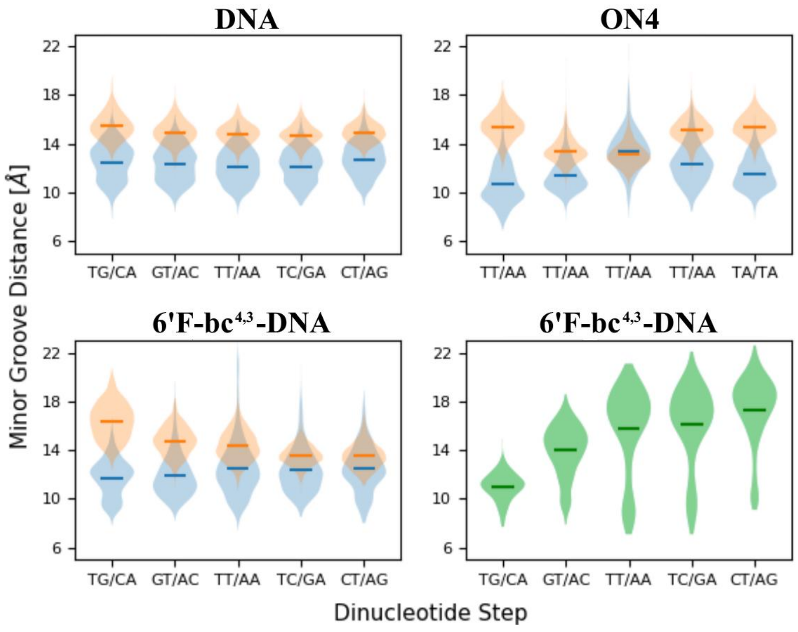


**Figure S8:** Minor groove distance of DNA, **ON4**, and 6’F-bc$^{4,3}$-DNA hybridized to DNA (blue) and RNA (red) and of the 6’F-bc$^{4,3}$-DNA homo-duplex (green) extracted from a 100 ns molecular dynamics trajectory.

## 8. Experimental Part

### 8.1 General

The chemicals used for the synthesis were purchased from Sigma Aldrich, TCI, ABCR, Acros Organics, SynQuest Labs, Apollo Scientific, Carbosynth, and Fluka in the highest purity available. All reactions were performed under an atmosphere of argon in oven-dried glassware. The anhydrous solvents for the reactions were obtained either by filtration through activated aluminium oxide or by storage over activated molecular sieves (4 Å). The solvents used for column chromatography (CC) were of technical grade and distilled prior to use. CC was performed on silica gel (SiliCycle, Silica Flash® P60, particle size 40–63 μm or Sigma Aldrich, pore size 60 Å, 230–400 mesh, neutralized with 0.1% w/Ca) as the stationary phase. Thin-layer chromatography (TLC) was performed on pre-coated silica gel plates (Marchery-Nagel, SIL G-25 $UV_{254}$). Visualisation was achieved either under UV light ($\lambda_{max}$ 254 nm) or by staining in dip solution [*p*-anisaldehyde (10 ml), acetic acid (2 ml), concentrated $H_2SO_4$ (10 ml), and ethanol (180 ml)], followed by heating with a heat gun. NMR spectra were recorded at room temperature on a Bruker Avance III HD 300, Bruker Avance II 400, or Bruker Avance III HD 400 instrument. Chemical shifts (δ) are reported relative to the undeuterated residual solvent peak [$CHCl_3$: 7.26 ppm ($^1H$) and 77.16 ppm ($^{13}C$); $CHD_2CD_3SO$: 2.50 ppm ($^1H$) and 39.52 ppm ($^{13}C$)]. APT, COSY, HSQC and HMBC experiments were routinely used to assign $^1H$ and $^{13}C$ signals. Difference $^1H\{^1H\}$-NOE and $^1H/^1H$-ROESY experiments were recorded at 400 MHz. The chemical shifts of $^{19}F$ and $^{31}P$ NMR are reported relative to 80% $CFCl_3$ or 85% $H_3PO_4$ as the external standard. Electrospray ionisation mass spectrometry (ESI) was performed on a LTQ Orbitrap XL instrument in positive or negative ion mode.

### 8.2 UV-melting curves

The UV-melting curves were recorded on a Varian Cary Bio 100 UV/vis spectrometer. The absorbance was monitored at 260 nm. Three cooling-heating circles (80 → 15 or 0 °C) were performed for every sample with a temperature gradient of 0.5 °C/min. The maxima of the curves first derivative was extracted with the Varian WinUV software and the $T_m$ values were reported as an average of at least five ramps (±1 °C error). All measurements were carried out in a NaCl (150 mM)/$NaH_2PO_4$ (10 mM) buffer (pH 7) with a duplex concentration of 2 μM. The samples were covered with a layer of dimethylpolysiloxane to avoid the evaporation of the solvent during the measurement.

### 8.3 Circular dichroism spectroscopy

The CD spectra were recorded on a Jasco J-715 spectropolariometer equipped with a Jasco PFO-350S temperature controller using the Spectra Manager v1.54 software. All spectra were measured in triplicates in the range of 210–320 nm at a scanning speed of 50 nm/min at 20 °C. They were baseline-corrected with a blank spectra (NaCl (150mM)/$NaH_2PO_4$ (10 mM) buffer pH 7), smoothed and normalized (320 nm = 0 mdeg). The sample conditions were the same as for the UV melting curves.

### 8.4 Molecular modeling

The potential energy profiles of nucleoside **8** versus pseudorotation phase angles were carried out with the Gaussian 09 software [1] by using the MP2 theory (second order Møller–Plesset perturbation theory) and the 6-311G* basis set. The energy profiles were obtained in varying the pseudorotation phase angle *P* from 0–360° (in 10° steps) at the range of $\nu_{max}$ (maximum puckering amplitude). The output was afterwards visualised with the OriginPro 2016 software. The molecular dynamics (MD) simulations were carried out using the GROMACS 5.0.6 simulation package [2] and the Amber 94 force field [3]. The additional force field parameters for the modified nucleosides were obtained using the R.E.D. III.5 tools package [4]. The duplex structures for the simulation were constructed with the Discovery Studio 4.5 program. The minimal sugar conformation ($\nu_{max}$ = 35, *P* = 160°) form the potential energy profile of nucleoside **8** was used to construct the duplexes, and were therefore combined with the different nucleobases. The starting duplex conformation was of B-type when the complementary strand was natural DNA and of A-type when the complementary strand consisted of natural RNA. For the homo 6'F-bc$^{4,3}$-DNA the starting duplex structure was in the B-conformation. After that, the duplexes were placed in a cubic cell at least 1.0 nm form the box edges away. The box was then filled with water (TIP3P) [5] and neutralized with $Na^+$ as counter ions. The cut-off for short range electrostatic interactions and van der Waals forces were set to 1.0 nm. The long range electrostatic interactions were calculated using Particle Mesh Ewald method [6]. The energy minimisation of the neutral structures was performed using the steepest descent algorithm. Then two equilibration steps were conducted, the first step was carried out under constant number of particles, volume and temperature (isothermal-isochoric ensemble) and the second one under constant number of particles, pressure and temperature (isothermal-isobaric ensemble). Both simulations were performed with a time step of 2 fs computed by leap-frog integrator, in periodic boundary conditions using the LINCS algorithm [7], and were run for 100 ps at 300 K. A position

restrained algorithm was applied on all the heavy atoms during these two steps, in order to prevent the system from collapsing. The resulting system was then subjected to the MD simulation where no restrains were applied and which was run for 100 ns. The MD trajectories were analyzed using the GROMACS package and the do_x3dna package [8].

### 8.5 Oligonucleotide synthesis and purification

The oligonucleotide synthesis was performed on a LBK Gene Assembler Plus (Pharmacia) DNA synthesiser in a 1.3 µM scale. The solid support (dA-Q-CPG 500, dmf-dG-Q-CPG 500) and the natural DNA phosphoramidites (dT, $dC^{4bz}$, $dG^{2DMF}$, $dA^{6Bz}$) were purchased from Glen Research. The natural DNA phosphoramidites were prepared as a 0.1 M solutions in ACN, whereas the modified phosphoramidites were prepared as 0.15 M solution in ACN. The coupling time for natural phosphoramidites was 90 s. For the modified phosphoramidites, the coupling time was extended to 12 min to achieve high coupling efficiency (based on trityl assay). As a coupling agent 5-(ethylthio)-1*H*-tetrazole (0.25 M in ACN) was used. The capping was performed with a solution of DMAP in ACN (0.5 M, Cap A) and a solution of 25% $Ac_2O$ and 12.5% *sym*-collidine in ACN (Cap B). For the oxidation was either used a solution of 20 mM $I_2$ and 0.45 M *sym*-collidine in 2:1 ACN/$H_2O$ (**ON1**, **ON2**) or a solution of 1.1 M TBHP in DCM (**ON3**–**ON7**). The detritylation step was conducted with a solution of 3% dichloroacetic acid in dichloroethane.

The deprotection of the oligonucleotides as well as the cleavage from the solid support was achieved by treatment with concentrated ammonia at 55 °C for 16 h. After cooling back to rt, the solutions were centrifuged, the supernatants were collected, and the remaining beads were washed with MQ-water (3 × 500 µL). The combined supernatants were filtered using spin-columns (Amicon Ultra 0.5 ml centrifugal filters, MWCO 3kDa) and concentrated to dryness a the Speed-Vac (UniVapo 150, UniEquip).

The crude oligonucleotides were purified by ion-exchange HPLC (DNAPac PA200, Dionex) on an ÄktaTM basic 10/100 system (Amersham Pharmacia Biotech). As mobile phase the buffers “A” (25 mM Tizma in $H_2O$, pH 8) and “B” (25 mM Trizma, 1.25 M NaCl in $H_2O$, pH 8) were used. A linear gradient of B in A was applied over 50 min with a flow rate of 1 ml/min and detection at 260 nm. The purified oligonucleotides were desalted, quantified and analyzed by $ESI^-$ mass spectrometry or by LC–MS. The purities were measured by HPLC. The natural DNA and RNA oligonucleotides were purchased from Microsynth and purified by HPLC and desalted if necessary.

Desalting protocol: The oligonucleotides were desalted using Sep-Pak Classic C18 Cartridges (Waters). The longer end of the column was attached to a 10 mL syringe without

the plunger. The column was washed with 5 mL ACN and 10 mL $H_2O$. Then it was conditioned with 2.5 mL 10 mM $AcONH_4$ solution. Afterwards the oligonucleotide was loaded as a water solution (max. of 4 mL as total volume) on the column. Following that, it was washed with 5 mL 1 M $AcONH_4$ solution and 10 mL water, before the oligonucleotide was eluted with 7 mL of a 3:2 $H_2O$/MeOH solution. The fractions of the column were quantified by measuring the absorbance at 260 nm with a Nanodrop spectrometer (Thermo Scientific). The fractions containing the oligonucleotide were combined, concentrated, dissolved in $H_2O$ and stored at −18 °C.

**Table S4:** List of synthesized oligonucleotides containing the modified nucleotides 6'F-bc$^{4,3}$-T or 6'F-bc$^{4,3}$-C and their characterization by ESI$^-$–MS or LC–MS.

| Entry | Sequence[a] | Modification | $m/z_{calcd}$ | $m/z_{found}$ |
|---|---|---|---|---|
| **ON1** | 5'-d(GGA TGT TCt CGA)-3' | 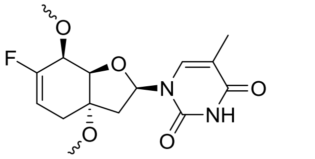 | 3732.5 | 3732.0 |
| **ON2** | 5'-d(GGA tGT TCT CGA)-3' | | 3732.5 | 3732.0 |
| **ON3** | 5'-d(GGA TGt tCT CGA)-3' | | 3788.5 | 3787.2 |
| **ON4** | 5'-d(GCA ttt ttA CCG)-3' | | 3891.6 | 3890.2 |
| **ON5** | 5'-d(GGA TGT TcT CGA)-3' | 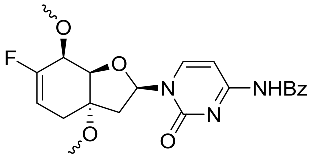 | 3732.5 | 3731.0 |
| **ON6** | 5'-d(GGA TGT TCT cGA)-3' | | 3732.5 | 3731.0 |
| **ON7** | 5'-d(GGA TGT TcT cGA)-3' | | 3788.5 | 3787.0 |

[a]Lowercase letter: modified nucleotide, capital letters: natural DNA.

**Table S5:** List of synthesized oligonucleotides containing the modified nucleotides 6'F-bc$^{4,3}$-T or 6'F-bc$^{4,3}$-C and their characterization by ion exchange HPLC.

| Entry | Sequence[a] | Modification | HPLC Gradient[b] | Retention Time[c] [min] | Purity [%] |
|---|---|---|---|---|---|
| **ON1** | 5'-d(GGA TGT TCt CGA)-3' | 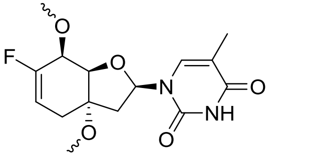 | B: 10 → 32% | 8.91 | 98.1 |
| **ON2** | 5'-d(GGA tGT TCT CGA)-3' | | B: 10 → 50% | 9.87 | 94.8 |
| **ON3** | 5'-d(GGA TGt tCT CGA)-3' | | B: 0 → 50% | 10.37 | 100.0 |
| **ON4** | 5'-d(GCA ttt ttA CCG)-3' | | B: 10 → 60% | 10.69 | 99.5 |
| **ON5** | 5'-d(GGA TGT TcT CGA)-3' | 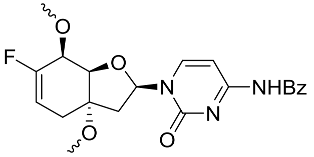 | B: 20 → 45% | 14.88 | 97.0 |
| **ON6** | 5'-d(GGA TGT TCT cGA)-3' | | B: 30 → 45% | 13.04 | 90.8 |
| **ON7** | 5'-d(GGA TGT TcT cGA)-3' | | B: 20 → 45% | 12.31 | 97.4 |

[a]Lowercase letter: modified nucleotide, capital letters: natural DNA. [b]Mobile phase A: 25 mM Trizma in $H_2O$, pH 8; mobile phase B: 25 mM Trizma, 1.25 M NaCl in $H_2O$, pH 8. [c]**ON1**: B = 20 → 35%, length CV = 1.5; **ON2–4**: B = 20 → 40%, length CV = 2, **ON5**, **7**: B = 30 → 60%, length CV = 4; **ON6**: B = 35 → 60% length CV = 4; flow rate for all ONs 1 mL/min.

## 8.6 Numbering of the sugars and nucleosides

Sugars Nucleosides

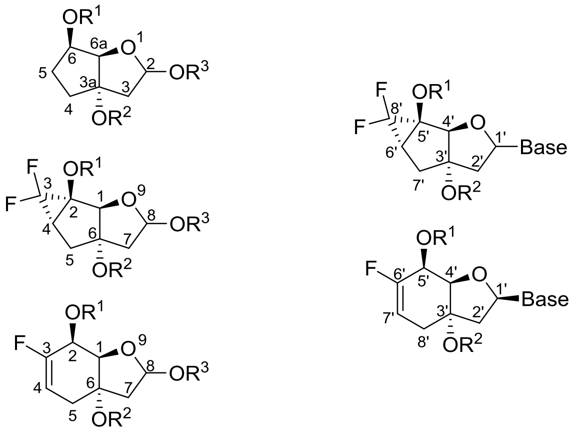


## 8.7 Synthesis and characterization

**(2*S*,3a*S*,6a*S*)-2-Methoxy-6-triethylsilanyloxy-3a-trimethylsilanyloxy-3,3a,4,6a-tetrahydro-2*H*-cyclopenta[*b*]furan (2α)**

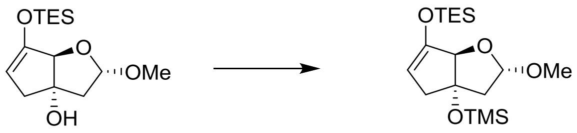


**1α** **2α**

BSA (2.6 ml, 10.63 mmol, 2.5 equiv) was added dropwise to a solution of the sugar **1α** (1.21 g, 4.25 mmol) in dry DCM (5 mL). After stirring for 17.4 h at rt the reaction mixture was evaporated to dryness. CC (hex/EtOAc 500:1 → 100:1) yielded the TMS-protected sugar **2α** (1.31 g, 3.66 mmol, 86%) as a colourless oil.

Data for **1α**: $R_f$ = 0.63 (hex/EtOAc 9:1); the other analytical data were in accordance with the literature [9].

**(2*R*,3a*S*,6a*S*)-2-Methoxy-6-triethylsilanyloxy-3a-trimethylsilanyloxy-3,3a,4,6a-tetrahydro-2*H*-cyclopenta[*b*]furan (2β)**

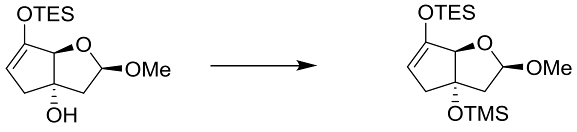


**1β** **2β**

BSA (13.1 ml, 53.5 mmol, 2.5 equiv) was added dropwise to a solution of bicyclo sugar **1β** (6.14 g, 21.4 mmol) in dry DCM (25 mL). The reaction mixture was stirred for 17.9 h at rt, before it was evaporated to dryness. CC (hex/EtOAc 500:1 → 100:1) yielded the TMS-protected sugar **2β** (6.77 g, 6.8 mmol, 88%) as a colourless oil.

Data for **1β**: $R_f$ = 0.74 (hex/EtOAc 9:1); the other analytical data were in accordance with the literature [10].

**(1*S*,2*R*,4*S*,6*S*,8*S*)-2-[(Triethylsilyl)oxy]-3,3-difluroro-8-methoxy-6-[(trimethylsilyl)oxy]-9-oxatricyclo[4.3.0$^{1,6}$.0$^{2,4}$]nonane (3α)**

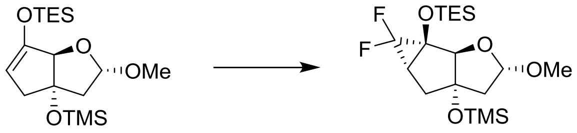


**2α** **3α**

The silyl enol ether **2α** (3.0 g, 8.4 mmol), NaI (252 mg, 1.7 mmol, 0.2 equiv), $TMSCF_3$ (3.1 mL, 21.0 mmol, 2.5 equiv) and dry THF (17 mL) were added to a pressure tube. The tube was closed and put in an oil bath at 70 °C. After 2 h the pressure tube was taken out of the oil bath and cooled down to rt. The reaction mixture was evaporated to dryness. The crude was diluted with $Et_2O$ (200 mL) and washed with $H_2O$ (200 mL), sat. $Na_2SO_3$ sol. (200 mL), sat. $NaHCO_3$ sol. (200 mL) and $H_2O$ (200 mL). The organic phase was dried ($MgSO_4$) and concentrated. CC (hex → hex/$Et_2O$ 10:1) yielded the *exo*-tricyclo sugar **3α** (2.578 g, 6.3 mmol, 75%) as a colourless oil.

Data for **3α**: $R_f$ = 0.16 (hex/$Et_2O$ 20:1); $^1H$ NMR (300 MHz, $CDCl_3$) δ 5.14 (*dd*, *J* = 5.9, 1.9 Hz, 1H, H-C(8)), 4.37 (*s*, 1H, H-C(1)), 3.39 (*s*, 3H, *Me*O), 2.34 – 2.26 (*m*, 1H, H-C(5)), 2.30 (*dd*, *J*

= 13.9, 5.9 Hz, 1H, H-C(7)), 2.18 (*dd*, *J* = 13.9, 1.9 Hz, 1H, H-C(7)), 1.93 – 1.85 (*m*, 2H, H-C(4), H-C(5)), 0.98 (*t*, *J* = 7.9 Hz, 9H, $(CH_3CH_2)_3Si$), 0.69 (m, 6H, $(CH_3CH_2)_3Si$), 0.12 (s, 9H, $Me_3Si$); $^{13}C$ NMR (101 MHz, $CDCl_3$) δ 113.9 (*dd*, *J* = 311.1, 284.6 Hz, C(3)), 106.7 (C(8)), 91.3 (*d*, *J* = 3.3 Hz, C(6)), 88.6 (C(1)), 68.1 – 67.0 (*m*, C(2)), 55.4 (*Me*O), 48.2 (C(7)), 35.6 (*d*, *J* = 3.0 Hz, C(5)), 33.1 (*dd*, *J* = 13.0, 9.4 Hz, C(4)), 6.7 (($(CH_3CH_2)_3Si$), 5.2 (($(CH_3CH_2)_3Si$), 1.9 ($Me_3Si$); $^{19}F$ NMR (376 MHz, $CDCl_3$) δ -135.6 (*dd*, *J* = 162.0, 17.2 Hz, 1F), -144.9 (*d*, *J* = 162.0 Hz, 1F); $ESI^+$-HRMS *m/z* calcd for $C_{18}H_{34}O_4F_2NaSi_2$ $[(M + Na)^+]$ 431.1856, found 431.1870.

### (1*S*,2*R*,4*S*,6*S*,8*R*)-2-[(Triethylsilyl)oxy]-3,3-difluroro-8-methoxy-6-[(trimethylsilyl)oxy]-9-oxatricyclo[4.3.0$^{1,6}$.0$^{2,4}$]nonane (3β)

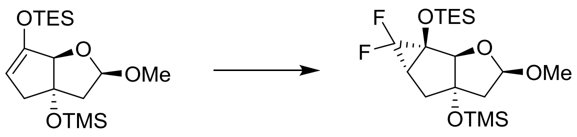


**2β** **3β**

The silyl enol ether **2β** (2.75 g, 7.7 mmol), NaI (231 mg, 1.5 mmol, 0.2 equiv), dry THF (17 mL and $TMSCF_3$ (2.9 mL, 19.6 mmol, 2.5 equiv) were added in that order to a pressure tube. The tube was closed and put in an oil bath at 70 °C for 4.5 h. Then the reaction mixture was cooled down to rt and evaporated to dryness. The crude was diluted with $Et_2O$ (100 mL) and washed with $H_2O$ (100 mL), sat. $Na_2SO_3$ sol. (100 mL), sat. $NaHCO_3$ sol. (100 mL) and $H_2O$ (100 mL). The organic phase was dried ($MgSO_4$) and concentrated. Purification by CC (hex → hex/ $Et_2O$ 250:1) gave the tricyclo sugar **3β** (2.03 g, 5 mmol, 65%) as a colourless oil.

Data for **3β**: $R_f$ = 0.37 (hex/$Et_2O$ 20:1); $^1H$ NMR (300 MHz, $CDCl_3$) δ 5.12 (*t*, *J* = 3.4 Hz, 1H, H-C(8)), 4.30 (*s*, 1H, H-C(1)), 3.41 (*s*, 3H, *Me*O), 2.65 – 2.52 (*m*, 1H, H-C(5)), 2.22 (*d*, *J* = 3.4 Hz, 2H, H-C(7)), 1.99 (*d*, *J* = 13.8 Hz, 1H, H-C(5)), 1.96 – 1.86 (*m*, 1H, H-C(4)), 1.02 – 0.95 (*m*, 9H, $(CH_3CH_2)_3Si$), 0.76 – 0.65 (*m*, 6H, $(CH_3CH_2)_3Si$), 0.11 (s, 9H, $Me_3Si$); $^{13}C$ NMR (101 MHz, $CDCl_3$) δ 114.4 (*dd*, *J* = 311.9, 285.3 Hz, C(3)), 107.0 (C(8)), 92.1 – 92.0 (*m*, C(6)), 91.3 (C(1)), 69.3 (*dd*, *J* = 11.2, 7.7 Hz, C(2)), 55.7 (*Me*O), 49.8 (C(7)), 38.1 (*d*, *J* = 2.5 Hz, C(5)), 34.7 (*dd*, *J* = 12.4, 8.6 Hz, C(4)), 6.8 (($(CH_3CH_2)_3Si$), 5.3 (*d*, *J* = 1.6 Hz, $(CH_3CH_2)_3Si$), 1.9 ($Me_3Si$); $^{19}F$ NMR (282 MHz, $CDCl_3$) δ -135.1 (*dd*, *J* = 163.4, 17.4 Hz, 1F), -145.3 (*d*, *J* = 163.4 Hz, 1F); $ESI^+$-HRMS *m/z* calcd for $C_{18}H_{34}O_4F_2NaSi_2$ $[(M + Na)^+]$ 431.1856, found 431.1862.

**(1*S*,2*R*,4*S*,6*S*)-2-[(Triethylsilyl)oxy]-3,3-difluroro-6-[(trimethylsilyl)oxy]-9-oxatricyclo-[4.3.0$^{1,6}$.0$^{2,4}$]non-7-ene (4)**

**and**

**(1*S*,2*R*,4*S*,6*S*,8*R* and *S*)-2-[(triethylsilyl)oxy]-3,3-difluroro-8-hydroxy-6-[(trimethylsilyl)oxy]-9-oxatricyclo[4.3.0$^{1,6}$.0$^{2,4}$]nonane (5α/β)**

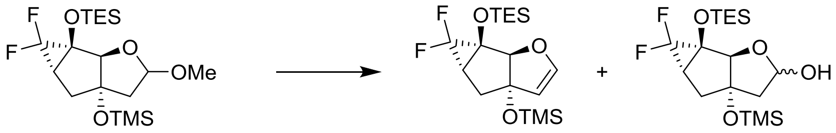


**3α** or **3β** **4** **5α/β**

Starting material **3α**: To a solution of the tricyclo sugar **3α** (5.42 g, 13.3 mmol) in dry DCM (25 mL) was added 2,6-lutidine (4.6 mL, 39.5 mmol, 3 equiv) at rt. The resulting solution was cooled to 0 °C. TMSOTf (3.6 mL, 19.9 mmol, 1.5 equiv) was added dropwise, and the reaction mixture was stirred for 2.1 h. During this period of time it was allowed to warm to rt. Then it was diluted with $Et_2O$ (200 mL) and washed with sat. $NaHCO_3$ sol. (2 × 200 mL). The combined aqueous phase was extracted with $Et_2O$ (3 × 400 mL). The combined organic phases were dried ($MgSO_4$) and concentrated. The crude product was purified by CC (hex → hex/$Et_2O$ 1:1) to yield the glycal **4** (2.04 g, 5.4 mmol, 41%) as a colourless oil and an anomeric mixture of tricyclo compound **5α/β** (2.03 g, 5.1 mmol, 39 %) as a light yellow oil.

Starting material **3β**: 2,6-lutidine (3.2 mL, 27.48 mmol, 3 equiv) was added to a solution of the sugar **3β** (3.72 g, 9.10 mmol) in dry DCM (30 mL) at rt. The resulting solution was cooled to 0 °C. After stirring for 21 min at 0 °C, TMSOTf (2.5 mL, 13.81 mmol, 1.5 equiv) was added dropwise and the reaction mixture was allowed to warm to rt. After another 6.25 h the reaction mixture was diluted with $Et_2O$ (150 L and washed with sat. $NaHCO_3$ sol. (2 × 150 mL). The combined aqueous phase was extracted with $Et_2O$ (3 × 300 mL). The combined organic phases were dried ($MgSO_4$), filtered and the solvent was evaporated. The crude product was purified by CC (hex → hex/$Et_2O$ 1:1) to yield the glycal **4** (1.99 g, 5.28 mmol, 58%) as a colourless oil and the tricyclo compound **5α/β** (1.04 g, 2.64 mmol, 29%) as a light yellow oil.

Data for **4**: $R_f$ = 0.60 (hex/$Et_2O$ 20:1); $^1$H NMR (400 MHz, $CDCl_3$) δ 6.43 (*d*, *J* = 2.6 Hz, 1H, H-C(8)), 5.04 (*d*, *J* = 2.6 Hz, 1H, H-C(7)), 4.56 (*d*, *J* = 1.2 Hz, 1H, H-C(1)), 2.47 (*ddt*, *J* = 13.7, 7.9, 2.1 Hz, 1H, H-C(5)), 2.03 (*d*, *J* = 13.7 Hz, 1H, H-C(5)), 1.88 (*ddd*, *J* = 16.7, 7.9, 1.0 Hz, 1H, H-C(4)), 0.97 (*t*, *J* = 7.9 Hz, 9H, $(CH_3CH_2)_3Si$), 0.67 (*m*, 6H, $(CH_3CH_2)_3Si$), 0.09 (*s*, 9H,

$Me_3$Si); $^{13}$C NMR (101 MHz, CDCl$_3$) δ 147.4 (C(8)), 113.2 (*dd*, *J* = 307.2, 287.9 Hz, C(3)), 108.2 (C(7)), 95.4 (*dd*, *J* = 4.8, 1.9 Hz, C(6)), 90.6 – 90.4 (*m*, C(1)), 67.5 (*dd*, *J* = 11.3, 7.9 Hz, C(2)), 37.8 (*d*, *J* = 4.1 Hz, C(5)), 33.0 (*dd*, *J* = 12.3, 8.4 Hz, C(4)), 6.7 (($CH_3CH_2)_3$Si), 5.3 (($CH_3CH_2)_3$Si), 1.9 ($Me_3$Si); $^{19}$F NMR (282 MHz, CDCl$_3$) δ -138.3 (*dd*, *J* = 163.1, 16.9 Hz, 1F), -148.3 (*d*, *J* = 163.1 Hz, 1F); ESI$^+$-HRMS *m/z* calcd for $C_{17}H_{31}O_3F_2Si_2$ [(M + H)$^+$] 377.1774, found 377.1770.

Data for **5α/β**: $R_f$ = 0.79 (hex/Et$_2$O 1:1); $^1$H NMR (400 MHz, CDCl$_3$) δ 5.64 (*ddd*, *J* = 7.5, 5.2, 3.9 Hz, 1H, H-C(8)), 5.44 (*dd*, *J* = 12.0, 5.1 Hz, 1H, H-C(8)), 4.41 (*s*, 1H, H-C(1)), 4.26 (*s*, 1H, H-C(1)), 3.69 (*d*, *J* = 12.0 Hz, 1H, O*H*), 3.01 (*d*, *J* = 7.5 Hz, 1H, O*H*), 2.47 – 2.41 (*m*, 1H, H-C(5)), 2.41 (*dd*, *J* = 14.0, 5.2 Hz, 1H, H-C(7)), 2.31 – 2.24 (*m*, 1H, H-C(5)), 2.23 (*dd*, *J* = 13.4, 5.1 Hz, 1H, H-C(7)), 2.07 (*dd*, *J* = 14.0, 4.0 Hz, 1H, H-C(7))), 2.03 (*d*, *J* = 13.4 Hz, 1H, H-C(7)), 1.96 (*ddd*, *J* = 17.0, 7.9, 1.3 Hz, 1H, H-C(4)), 1.88 (*ddd*, *J* = 16.7, 8.1, 1.8 Hz, 1H, H-C(4)), 1.81 (*d*, *J* = 13.5 Hz, 1H, H-C(5)), 1.78 (*dd*, *J* = 13.3, 1.8 Hz, 1H, H-C(5)), 0.97 (*q*, *J* = 8.0 Hz, 18H, 2x ($CH_3CH_2)_3$Si), 0.81 – 0.61 (*m*, 12H, 2x ($CH_3CH_2)_3$Si), 0.17 (s, 9H, $Me_3$Si), 0.12 (s, 9H, $Me_3$Si); $^{13}$C NMR (101 MHz, CDCl$_3$) δ 114.1 (*dd*, *J* = 308.5, 288.5 Hz, C(3)), 113.8 (*dd*, *J* = 308.1, 290.2 Hz, C(3)), 101.3, 101.3 (C(8)), 94.0 (*dd*, *J* = 5.9, 1.6 Hz, C(6)), 93.6 (*dd*, *J* = 6.7, 1.5 Hz, C(6)), 89.4 – 89.2 (*m*, C(1)), 87.5 – 87.4 (*m*, C(1)), 67.1 (*dd, J* = 11.8, 7.8 Hz, C(2)), 65.5 (*dd*, *J* = 11.4, 8.0 Hz, C(2)), 51.0, 46.2 (C(7)), 33.7 (*d*, *J* = 3.8 Hz, C(5)), 33.0 (*dd*, *J* = 12.5, 9.0 Hz, C(4)), 32.0 (*dd*, *J* = 12.6, 9.6 Hz, C(4)), 29.2 (*d*, *J* = 4.6 Hz, C(5)), 6.7 (2x ($CH_3CH_2)_3$Si), 5.3 (*d*, *J* = 0.7 Hz, ($CH_3CH_2)_3$Si), 5.2 (*d*, *J* = 1.8 Hz, ($CH_3CH_2)_3$Si), 1.8 (2x $Me_3$Si); $^{19}$F NMR (282 MHz, CDCl$_3$) δ -135.1 (*dd*, *J* = 162.1, 17.1 Hz, 1F), -137.6 (*dd*, *J* = 160.9, 16.7 Hz, 1F), -145.8 (*d*, *J* = 162.1 Hz), -146.3 (*d*, *J* = 160.9 Hz, 1F); ESI$^+$-HRMS *m/z* calcd for $C_{17}H_{32}O_4F_2NaSi_2$ [(M + Na)$^+$] 417.1699, found 417.1686.

**1-[(3'*S*,5'*R*,6'*S*)-2'-Deoxy-3',5'-ethano-5'-*O*-triethylsilyl-8',8'-dilfluoro-5',6'-methano-3'-*O*-trimethylsilyl-α,β-D-ribofuranosyl]thymine (6α/β)**

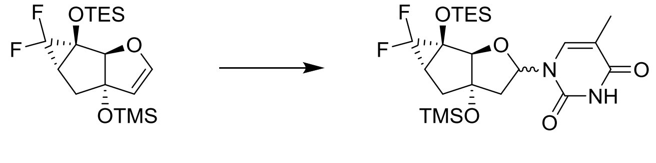


**4** **6α/β**

BSA (1.5 ml, 6.13 mmol, 4.4 equiv) was added dropwise to a suspension of thymine (530 mg, 4.20 mmol, 3 equiv) in dry DCM (7 mL) at rt. The mixture was stirred for 1.5 h

before a solution of the glycal **4** (528 mg, 1.40 mmol) in dry DCM (0.6 mL) was slowly added. The resulting mixture was cooled down to 0 °C and NIS (473 mg, 2.10 mmol, 1.5 equiv) was added in three portions over a period of 34 min. Afterwards the reaction mixture was stirred for an additional 71 min at 0 °C and for 3.1 h at rt. The orange solution was then diluted with $Et_2O$ (40 mL) and washed with sat. $Na_2O_3S_2$ sol. (2 × 40 mL) and sat. $NaHCO_3$ sol. (40 mL). The combined aqueous phase was extracted with $Et_2O$ (3 × 120 mL). The combined organic phase was dried ($MgSO_4$), concentrated and dried at HV. The crude mixture (1.303 g, >100%) was subjected to the next reaction without further purification.

To a suspension of the crude (1.303 g, 1.40 mmol) in dry toluene (14 mL) was added $Bu_3SnH$ (0.56 mL, 2.08 mmol, 1.5 equiv) and AIBN (115 mg, 0.70 mmol, 0.5 equiv) at rt. Then the reaction mixture was heated up to 90 °C and was stirred for 31 min at this temperature. After cooling down to rt the solvent was removed under reduced pressure. The residue was adsorbed on silica gel and purified by CC (hex/$Et_2O$ 10:1 → 1:5) to yield an anomeric mixture of the tricyclo nucleoside **6α/β** (496 mg, 0.99 mmol, 70% over two steps, α/β-ratio 1:4.5).

Data for **6α/β**: $R_f$ = 0.30 (hex/$Et_2O$ 1:2); $^1$H NMR (400 MHz, $CDCl_3$) δ 9.39 (*s*, 1H, H-N(3)), 9.34 (*s*, 1H, H-N(3)), 7.70 (*d*, *J* = 1.3 Hz, 1H, H-C(6)), 7.57 (*d*, *J* = 1.3 Hz, 1H, H-C(6)), 6.51 (*dd*, *J* = 9.6, 4.8 Hz, 1H, H-C(1')), 6.36 (*dd*, *J* = 8.2, 2.0 Hz, 1H, H-C(1')), 4.43 (*s*, 1H, H-C(4')), 4.27 (*s*, 1H, H-C(4')), 2.61 (*dd*, *J* = 14.8, 8.2 Hz, 1H, H-C(2')), 2.37 (*dd*, *J* = 13.2, 4.8 Hz, 1H, H-C(2')), 2.33 – 2.24 (*m*, 2H, 2x H-C(7')), 2.16 (*dt*, *J* = 14.8, 1.5 Hz, 1H, H-C(2')), 2.12 – 2.00 (*m*, 1H, H-C(6')), 2.07 (*dd*, *J* = 13.2, 9.6 Hz, 1H, H-C(2')), 1.97 – 1.89 (*m*, 1H, H-C(6')), 1.95 – 1.92 (*m*, 6H, 2x *Me*-C(5)), 1.81 – 1.72 (*m*, 2H, 2x H-C(7')), 0.99 – 0.89 (*m*, 18H, $(CH_3CH_2)_3Si$), 0.73 – 0.60 (*m*, 12H, $(CH_3CH_2)_3Si$), 0.15 (*s*, 9H, $Me_3Si$), 0.11 (*s*, 9H, $Me_3Si$); $^{13}$C NMR (101 MHz, $CDCl_3$) δ 164.2, 164.1 (C(4)), 150.6, 150.5 (C(2)), 136.9, 136.4 (C(6)), 113.8 (*dd*, *J* = 307.8, 291.0 Hz, C(8')), 113.4 (*dd*, *J* = 307.3, 288.2 Hz, C(8')), 111.1, 110.3 (C(5)), 93.6 – 93.4 (*m*, C(3')), 92.2 (*d*, *J* = 6.9 Hz, C(3')), 89.2 (*d*, *J* = 3.8 Hz, C(4')), 88.8 (*d*, *J* = 2.9 Hz, C(4')), 86.6, 86.4 (C(1')), 66.8 (*dd*, *J* = 11.2, 8.0 Hz, C(5')), 65.4 (*dd*, *J* = 11.6, 8.2 Hz, C(5')), 45.8, 45.4 (C(2')), 33.1 (*dd*, *J* = 12.0, 9.1 Hz, C(6')), 31.7 (*dd*, *J* = 12.5, 9.1 Hz, C(6')), 31.2 (*d*, *J* = 4.8 Hz, C(7')), 29.5 (*d*, *J* = 4.0 Hz, C(7')), 12.8, 12.7 (*Me*-C(5)), 6.7, 6.6 ($(CH_3CH_2)_3Si$), 5.2 ($(CH_3CH_2)_3Si$), 4.9 (*d*, *J* = 1.9 Hz, $(CH_3CH_2)_3Si$)), 1.8, 1.8 ($Me_3Si$); $^{19}$F NMR (282 MHz, $CDCl_3$) δ -135.3 (*dd*, *J* = 163.1, 17.0 Hz, 1F), -136.4 (*dd*, *J* = 161.2, 17.0 Hz, 1F), -145.8 (*d*, *J* = 161.2 Hz, 1F), -146.6 (*d*, *J* = 163.1 Hz, 1F); $ESI^+$-HRMS *m/z* calcd for $C_{22}H_{37}O_5N_2F_2Si_2$ [$(M + H)^+$] 503.2204, found 503.2191.

**1-[(3'*S*)-2'-Deoxy-6'-fluoro-3',5'-prop-6'-en-5'-on-β-D-ribofuranosyl]thymine (7β)**

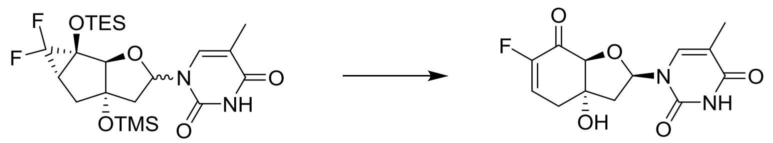


**6α/β** **7β**

The tricyclic nucleoside **6α/β** (402 mg, 0.80 mmol) was dissolved in a mixture of dry DCM (7.5 mL) and dry pyridine (1.5 mL). The resulting solution was cooled to 0 °C, and HF-pyridine (≈70% HF, 0.33 mL, 12.7 mmol, 15.9 equiv) was added dropwise. The reaction mixture was stirred for 33 min at 0 °C and for 1 h at rt. Then silica gel (1.8 g) was added and the solvent was removed under reduced pressure. CC (1% →5% MeOH in DCM) afforded the β-fluoroenone **7β** (168 mg, 0.57 mmol, 71%) as a white foam.

Data for **7β**: $R_f$ = 0.35 (10% MeOH in DCM); $^1$H NMR (400 MHz, DMSO-$d_6$) δ 11.30 (*br*, 1H, N*H*), 7.32 (*d*, *J* = 1.2 Hz, 1H, H-C(6)), 6.67 (*ddd*, *J* = 14.0, 6.1, 3.0 Hz, 1H, H-C(7')), 6.12 (*dd*, *J* = 9.1, 5.7 Hz, 1H, H-C(1')), 5.88 (*s*, 1H, O*H*), 4.41 (*s*, 1H, H-C(4')), 2.89 (*ddd*, *J* = 19.2, 6.1, 4.5 Hz, 1H, H-C(8')), 2.77 (*ddd*, *J* = 19.2, 6.6, 3.0 Hz, 1H, H-C(8')), 2.30 (*dd*, *J* = 13.3, 9.1 Hz, 1H, H-C(2')), 2.21 (*ddd*, *J* = 13.3, 5.7, 1.3 Hz, 1H, H-C(2')), 1.74 (*d*, *J* = 1.2 Hz, 3H, *Me*-C(5)); $^{13}$C NMR (101 MHz, DMSO-$d_6$) δ 189.8 (*d*, *J* = 18.6 Hz, (C(5')), 163.7 (C(4)), 151.6 (*d*, *J* = 257.7 Hz, C(6')), 150.3 (C(2)), 136.7 (C(6)), 124.0 (*d*, *J* = 14.7 Hz, C(7')), 108.9 (C(5)), 86.4 (C(1')), 86.2 (*d*, *J* = 5.8 Hz, C(4')), 78.3 (*d*, *J* = 1.4 Hz, C(3')), 40.7 (C(2')), 31.8 (*d*, *J* = 5.9 Hz, C(8')), 12.5 (*Me*-C(5)); $^{19}$F NMR (282 MHz, DMSO-$d_6$) δ -132.6 – -132.8 (*m*, 1F); ESI$^+$-HRMS *m/z* calcd for $C_{13}H_{14}O_5N_2F$ [(M + H)$^+$] 297.0881, found 297.0883.

**1-[(3'*S*,5'*S*)-2'-Deoxy-6'-fluoro-3',5'-prop-6'-eno-β-D-ribofuranosyl]thymine (8)**

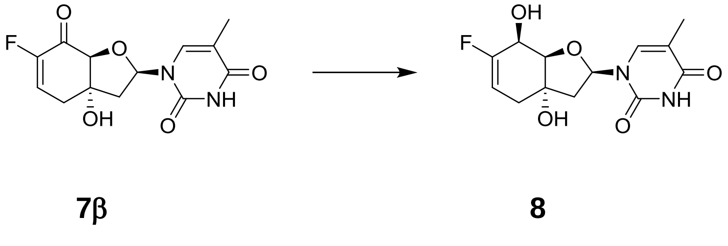


**7β** **8**

$CeCl_3 \cdot 7\ H_2O$ (320 mg, 0.86 mmol, 2.0 equiv) was added to a solution of the ketone **7β** (128 mg, 0.43 mmol) in dry MeOH (6 mL). The resulting solution was cooled to 0 °C. $NaBH_4$

(33 mg, 0.87 mmol, 2.0 equiv) was added in three portions over a period of 22 min. After stirring for additional 60 min at 0 °C, the reaction mixture was diluted with EtOAc (50 mL) and quenched with sat. $NH_4Cl$ sol. (50 mL). The aqueous phase was extracted with EtOAc (14 × 50 mL). The combined organic phases were dried ($MgSO_4$) and concentrated. Purification by CC (1% →4% MeOH in DCM) yielded the diol **8** (118 mg, 0.40 mmol, 92%) as white foam.

Data for **8**: $R_f$ = 0.20 (10% MeOH in DCM); $^1$H NMR (400 MHz, DMSO-$d_6$) $\delta$ 11.28 (*s*, 1H, N*H*), 8.05 (*d*, *J* = 1.2 Hz, 1H, H-C(6)), 6.20 (*dd*, *J* = 9.8, 5.1 Hz, 1H, H-C(1')), 6.00 (*d*, *J* = 5.4 Hz, 1H, *H*O-C(5')), 5.41 – 5.32 (*m*, 1H, C(7')), 5.39 (*s*, 1H, *H*O-C(3')), 4.24 (*dt*, *J* = 10.9, 5.0 Hz, 1H, H-C(5')), 3.95 (*d*, *J* = 5.2 Hz, 1H, H-C(4')), 2.63 (*ddd*, *J* = 17.6, 5.8, 3.5 Hz, 1H, H-C(8')), 2.31 (*dt*, *J* = 17.6, 5.3 Hz, 1H, (H-C(8')), 2.17 (*dd*, *J* = 12.4, 5.1 Hz, 1H, H-C(2')), 1.95 (*dd*, *J* = 12.4, 9.8 Hz, 1H, H-C(2')), 1.75 (*d*, *J* = 1.2 Hz, 3H, *Me*-C(5)); $^{13}$C NMR (101 MHz, DMSO-$d_6$) $\delta$ 163.7 (C(4)), 158.6 (d, *J* = 260.9 Hz, C(6')), 150.6 (C(2)), 136.8 (C(6)), 108.8 (C(5)), 102.4 (*d*, *J* = 15.4 Hz, C(7')), 85.8 (*d*, *J* = 9.9 Hz, C(4')), 83.4 (C(1')), 75.5 (C(3')), 64.7 (*d*, *J* = 26.1 Hz, C(5')), 44.2 (C(2')), 33.5 (*d*, *J* = 8.0 Hz, C(8')), 12.3 (*Me*-C(5)); $^{19}$F NMR (376 MHz, DMSO-$d_6$) $\delta$ -112.9 (*tt*, *J* = 12.2, 5.4 Hz, 1F); ESI$^+$-HRMS *m/z* calcd for $C_{13}H_{15}O_5N_2FNa$ [(M + Na)$^+$] 321.0857, found 321.0863.

**1-{(3'*S*,5'*S*)-2'-Deoxy-6'-fluoro-5'-*O*-[(4,4'-dimethyoxytriphenyl)methyl]-3',5'-prop-6'-eno-β-D-ribofuranosyl}thymine (9)**

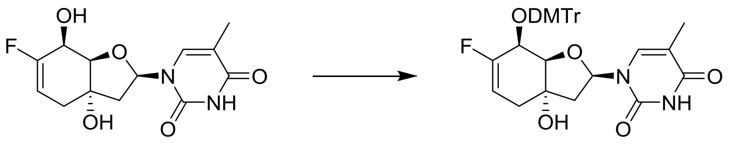


**8** **9**

The nucleoside **8** (112 mg, 0.38 mmol) was dissolved in dry pyridine (1.5 ml) at rt. DMTr-Cl (449 mg, 1.33 mmol, 3.5 equiv) was added in seven equivalent portions over a period of 4 days. Afterwards the reaction mixture was stirred for another 3 days at rt. Then it was diluted with EtOAc (20 mL) and washed with sat. $NaHCO_3$ sol. (2 × 20 mL). The combined aqueous phase was extracted with EtOAc (6 × 40 mL). The combined organic phases were dried ($MgSO_4$), concentrated and purified by CC (1% MeOH in DCM + 0.5% $Et_3N$). The DMTr-protected nucleoside **9** (172 mg, 0.29 mmol, 76%) was isolated as white foam.

Data for **9**: $R_f$ = 0.37 (3% MeOH in DCM); $^1$H NMR (400 MHz, DMSO-$d_6$) δ 11.37 (*br*, 1H, N*H*), 7.47 – 7.42 (*m*, 2H, H-arom), 7.36 – 7.26 (*m*, 7H, H-C(6), H-arom), 7.26 – 7.19 (*m*, 1H, H-arom), 6.90 – 6.85 (*m*, 4H, H-arom), 6.10 (*dd*, *J* = 9.6, 5.2 Hz, 1H, H-C(1’)), 5.37 (*s*, 1H, O*H*), 5.28 (*dt*, *J* = 11.2, 5.5 Hz, 1H, H-C(7’)), 4.23 (*dd*, *J* = 6.1, 4.4 Hz, 1H, H-(5’)), 3.73 (2*s*, 6H, 2x *Me*O), 3.57 (*dd*, *J* = 4.4, 2.3 Hz, 1H, H-C(4’)), 2.63 (*dt*, *J* = 15.6, 4.6 Hz, 1H, H-C(8’)), 2.27 (*dd*, *J* = 12.9, 5.2 Hz, 1H. H-C(2’)), 2.20 (*ddd*, *J* = 15.6, 6.1, 3.5 Hz, 1H, H-C(8’)), 1.98 (*dd, J* = 12.9, 9.6 Hz, 1H, H-C(2’)), 1.28 (*d*, *J* = 1.2 Hz, 3H, *Me*-C(5)); $^{13}$C NMR (101 MHz, DMSO-$d_6$) δ 163.5 (C(4)), 158.7 (*d*, *J* = 269.7 Hz, C(6’)) 158.3, 158.3 (C-arom), 150.2 (C(2)), 145.0, 135.4, 135.3 (C-arom), 135.2 (C(6)), 130.2, 127.9, 127.8, 126.9, 113.1, 113.1 (*C*H-arom), 109.5 (C(5)), 102.3 (*d*, *J* = 13.3 Hz, C(7’)), 87.5 (*C*(Ph)$_3$), 87.1 (*d*, *J* = 4.3 Hz, C(4’)), 83.0 (C(1’)), 77.3 (C(3’)), 67.8 (*d*, *J* = 23.4 Hz, (C(5’)), 55.1, 55.0 (*Me*O-DMTr), 45.3 (C(2’)), 33.5 (*d*, *J* = 6.6 Hz, C(8’)), 11.5 (*Me*-C(5)); $^{19}$F NMR (376 MHz, DMSO-$d_6$) δ -108.9 (*br*, 1F); ESI$^+$-HRMS *m/z* calcd for $C_{34}H_{33}O_7N_2FNa$ [(M + Na)$^+$] 623.2164, found 623.2161.

**1-{(3’*S*,5’*S*)-3’-*O*-[(2-Cyanoethoxy)-diisopropylaminophosphanyl]-2’-deoxy-6’-fluoro-5’-*O*-[(4,4’-dimethyoxytriphenyl)methyl]-3’,5’-prop-6’-eno-β-D-ribofuranosyl}thymine (10)**

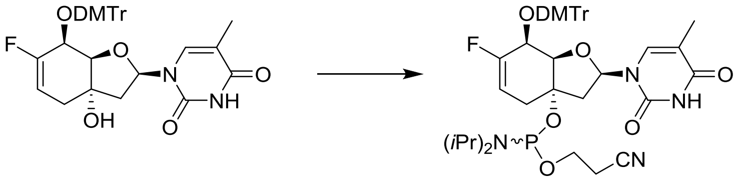


**9** **10**

To a solution of **9** (159 mg, 0.26 mmol) in dry THF (2 mL) was added DIPEA (0.18 mL, 1.03 mmol, 3.9 equiv) and CEP-Cl (0.12 mL, 0.54 mmol, 2.0 equiv). The resulting suspension was stirred at rt for 3.9 h. Afterwards the solvent was removed under reduced pressure and the residue was purified by CC (hex/EtOAc 1:1 + 0.5% $Et_3N$) to yield the phosphoramidite **10** (131 mg, 0.16 mmol, 62%) as a mixture of two isomers and as white foam.

Data for **10**: $R_f$ = 0.76 & 0.70 (hex/$Et_2O$ 1:5); $^1$H NMR (400 MHz, DMSO-$d_6$) δ 11.39 (*br*, 2H, 2x N*H*), 7.48 – 7.44 (*m*, 4H, H-arom), 7.37 – 7.26 (*m*, 14H, 2x H-C(6), H-arom), 7.25 – 7.19 (*m*, 2H, H-arom), 6.90 – 6.85 (*m*, 8H, H-arom), 6.13 (*dd*, *J* = 9.3, 5.1 Hz, 1H, H-C(1’)), 6.08 (*dd*, *J* = 9.3, 5.2 Hz, 1H, H-C(1’)), 5.32 (*dt*, *J* = 11.1, 5.3 Hz, 2H, 2x H-C(7’)), 4.34 (*t*, *J* = 5.0 Hz, 1H, H-C(5’)), 4.27 (*dd*, *J* = 6.3, 4.5 Hz, 1H, H-C(5’)), 3.81 – 3.77 (*m*, 1H, H-C(4’)), 3.73 (*s*, 12H, 4x *Me*O), 3.71 – 3.47 (*m*, 8H, 2x OC$H_2$CH$_2$CN, 2x (Me$_2$C*H*)$_2$N)), 3.65 – 3.62 (*m,* 1H, H-

C(4')), 2.85 – 2.68 (*m*, 2H, H-C(8')), 2.75 (*t*, *J* = 5.9 Hz, 2H, $OCH_2CH_2CN$), 2.70 (*t*, *J* = 5.8 Hz, 2H, $OCH_2CH_2CN$), 2.68 – 2.53 (*m*, 4H, 2x H-C(2'), 2x H-C(8')), 2.09 (*td*, *J* = 13.5, 9.4 Hz, 2H, H-C(2')), 1.34 (*d*, *J* = 1.2 Hz, 3H, *Me*-C(5)), 1.28 (*d*, *J* = 1.1 Hz, 3H, *Me*-C(5)), 1.14 – 1.09 (*m*, 18H, $(Me_2CH)_2N$), 1.05 (*d*, *J* = 6.7 Hz, 6H, $(Me_2CH)_2N$); $^{13}C$ NMR (101 MHz, DMSO-$d_6$) δ 163.5, 163.5 (C(4)), 158.6 (2*d*, *J* = 269.5 and 270.0 Hz, C(6')), 158.4, 158.3 (C-arom), 150.2, 150.1 (C(2)), 145.0, 144.9, 135.3, 135.3, 135.2, 135.1 (C-arom), 135.0, 134.9 (C(6)), 130.2, 127.8, 127.7, 126.9 (*C*H-arom), 119.0, 118.9 (*C*N), 113.1, 113.1 (*C*H-arom), 109.6 (*br*, C(5)), 102.0 (*d*, *J* = 14.4 Hz, C(7')), 101.7 (*d*, *J* = 14.3 Hz, C(7')), 87.7, 87.6 ($C(Ph)_3$), 86.6 – 86.3 (*m*, C(4')), 86.1 – 85.8 (*m*, C(4')), 83.0 (C(1')), 82.8 (*d*, *J* = 9.5 Hz, C(3')), 82.8 (C(1')), 82.7 (*d*, *J* = 8.4 Hz, C(3')), 67.5 (*d*, *J* = 23.5 Hz, C(5')), 57.9 (*d*, *J* = 7.5 Hz, $OCH_2CH_2CN$), 57.8 (*d*, *J* = 6.8 Hz, $OCH_2CH_2CN$), 55.0 (*Me*O-DMTr), 43.9, 43.8 (C(2')), 42.9, 42.7, 42.6 ($(Me_2CH)_2N$), 32.0 – 31.7 (*m*, C(8')), 24.3, 24.2, 24.1, 23.9, 23.9, 23.8, 23.8 ($(Me_2CH)_2N$), 19.7, 19.6 ($OCH_2CH_2CN$), 11.6, 11.4 (*Me*-C(5); $^{19}F$ NMR (376 MHz, DMSO-$d_6$) δ -107.9 (*br*, 1F), -108.6 (*br*, 1F); $^{31}P$ NMR (162 MHz, DMSO-$d_6$) δ 139.4, 139.1; ESI$^+$-HRMS *m/z* calcd for $C_{43}H_{50}O_8N_4FNaP$ $[(M + Na)^+]$ 823.3243, found 823.3255.

### (1*S*,2*R*,4*S*,6*S*,8*R* and *S*)-8-Acteyloxy-2-[(triethylsilyl)oxy]-3,3-difluroro-6-[(trimethylsilyl)oxy]-9-oxatricyclo[$4.3.0^{1,6}.0^{2,4}$]nonane (11α/β)

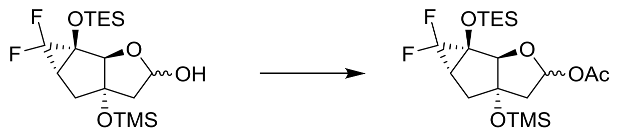


**5α/β** **11α/β**

A solution of the sugar **5α/β** (5.09 g, 12.9 mmol) in dry pyridine (25 mL) was cooled to 0 °C and then $Ac_2O$ (16.5 mL, 174.6 mmol, 13.5 equiv) was added. After 37 min the reaction mixture was allowed to warm to rt and was further stirred for 16.4 h. Afterwards the solvent was removed under reduced pressure. The crude product was purified by CC (hex/$Et_2O$ 20:1) to yield an anomeric mixture of the sugar derivative **11α/β** (4.9 g, 11.2 mmol, 87%) as a colourless oil.

Data for **11α/β**: $R_f$ = 0.31 & 0.25 (hex/$Et_2O$ 10:1); $^1H$ NMR (400 MHz, $CDCl_3$) δ 6.39 (*dd*, *J* = 5.0, 2.3 Hz, 1H, H-C(8)), 6.34 (*d*, *J* = 5.5 Hz, 1H, H-C(8)), 4.46 (*s*, 1H, H-C(1)), 4.35 (*s*, 1H, H-C(1)), 2.55 (*ddt*, *J* = 13.8, 7.3, 2.1 Hz, 1H, H-C(5)), 2.40 – 2.20 (*m*, 5H, H-C(5), 4x H-C(7)), 2.05 (*s*, 3H, $Me_3CO$), 2.05 – 2.03 (*m*, 1H, H-C(5)), 2.01 (*s*, 3H, $Me_3CO$), 1.93 (*dd*, *J* = 15.8,

7.5 Hz, 1H, H-C(4)), 1.93 – 1.85 (*m*, 1H, H-C(4)), 1.79 – 1.73 (*m*, 1H, H-C(5)), 0.95 (*td*, *J* = 7.9, 4.3 Hz, 18H, 2x ($CH_3CH_2)_3Si$), 0.72 – 0.61 (*m*, 12H, 2x ($CH_3CH_2)_3Si$), 0.13 (*s*, 9H, $Me_3Si$), 0.11 (*s*, 9H, $Me_3Si$); $^{13}C$ NMR (101 MHz, $CDCl_3$) δ 170.2, 170.0 ($Me_3CO$), 114.0 (*dd*, *J* = 310.1, 287.5 Hz, C(3)), 113.6 (*dd*, *J* = 308.5, 288.5 Hz, C(3)), 99.9, 99.2 (C(8)), 92.4 (*dd*, *J* = 4.1, 2.1 Hz, C(6)), 92.1 (*dd*, *J* = 5.9, 1.5 Hz, C(6)), 91.6 – 91.5 (*m*, C(1)), 89.7 (*d*, *J* = 3.1 Hz, C(1)), 68.3 (*dd*, *J* = 11.1, 7.7 Hz, C(2)), 66.2 (*dd*, *J* = 11.5, 8.2 Hz, C(2)), 49.0, 45.8 (C(7)), 37.1 (*d*, *J* = 3.2 Hz, C(5)), 34.1 (*dd*, *J* = 12.4, 8.7 Hz, C(4)), 32.3 (*dd*, *J* = 12.8, 9.5 Hz, C(4)), 32.2 (*d*, *J* = 4.4 Hz, C(5)), 21.5, 21.3 ($Me_3CO$), 6.7, 6,7 (($CH_3CH_2)_3Si$), 5.2 (($CH_3CH_2)_3Si$), 5.1 (d, *J* = 1.2 Hz, ($CH_3CH_2)_3Si$), 1.9, 1.8 ($Me_3Si$); $^{19}F$ NMR (282 MHz, $CDCl_3$) δ -136.0 (*dd*, *J* = 163.6, 16.9 Hz, 1F), -137.5 (*dd*, *J* = 161.6, 17.0 Hz, 1F), -145.8 (d, *J* = 163.6 Hz, 1F), -146.4 (d, *J* = 161.6 Hz, 1F); ESI$^+$-HRMS *m/z* calcd for $C_{19}H_{34}O_5F_2NaSi_2$ [(M + Na)$^+$] 459.1805, found 459.1794.

**1-[(3'*S*,5'*R*,6'*S*)-2'-Deoxy-3',5'-ethano-5'-*O*-triethylsilyl-8',8'-dilfluoro-5',6'-methano-3'-*O*-trimethylsilyl-β-D-ribofuranosyl]-4-*N*-benzoyl-cytosine (12β)**

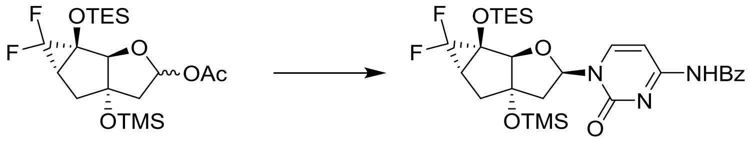


**11α/β** **12β**

To a suspension of *N*-benzoylcytosine (1.95g, 9.06 mmol, 2 equiv) in dry ACN (40 mL) was added BSA (5.5 mL, 22.49 mmol, 5 equiv). The mixture was stirred at rt for 63 min before it was cooled to 0 °C. The sugar **11α/β** (1.979 g, 4.53 mmol) dissolved in dry ACN (6 mL) and TMSOTf (1.6 mL, 8.84 mmol, 2 equiv) were added. After stirring for 37 min at 0 °C the reaction mixture was warmed to rt and stirring was continued for another 2.7 h. It was quenched with sat. $NaHCO_3$ sol. (350 mL) and extracted with $Et_2O$ (1 × 350 mL, 3 × 300 mL). The combined organic phases were dried ($MgSO_4$) and concentrated. Purification by CC (hex/$Et_2O$ 5:1 → 1:10) gave the β-nucleoside **12β** (1.088 g, 1.84 mmol, 41%) as a white foam.

Data for **12β**: $R_f$ = 0.33 (hex/$Et_2O$ 1:10); $^1H$ NMR (300 MHz, $CDCl_3$) δ 8.80 (*br*, 1H, N*H*), 8.55 (*d*, *J* = 7.5 Hz, 1H, H-C(6)), 7.98 – 7.79 (*m*, 2H, H-arom), 7.64 – 7.56 (*m*, 1H, H-arom), 7.55 – 7.46 (*m*, 3H, H-C(5), H-arom), 6.47 (*dd*, *J* = 8.0, 5.1 Hz, 1H, H-C(1')), 4.40 (*s*, 1H, H-C(4')), 2.71 (*dd*, *J* = 13.4, 5.1 Hz, 1H, H-C(2')), 2.28 – 2.17 (*m*, 1H, H-C(7')), 2.10 (*dd, J* = 13.4, 8.0

Hz, 1H, H-C(2’)), 2.04 (*dd*, *J* = 16.8, 7.5 Hz, 1H, H-C(6’)), 1.84 (*d*, *J* = 13.9 Hz, 1H, H-C(7’)), 1.01 – 0.94 (*m*, 9H, $(CH_3CH_2)_3Si$), 0.77 – 0.66 (*m*, 6H, $(CH_3CH_2)_3Si$), 0.15 (*s*, 9H, $Me_3Si$); $^{13}C$ NMR (101 MHz, $CDCl_3$) δ 167.1 (*br*, *C*ONH), 162.4 (C(4)), 154.8 (*br*, C(2)), 145.4 (C(6)), 133.3 (*C*H-arom), 133.2 (C-arom) , 129.1, 127.7 (*C*H-arom), 113.3 (*dd*, *J* = 308.3, 287.2 Hz, C(8’)), 96.7 (*br*, C(5)), 93.1 – 93.0 (*m*, C(3’)), 90.3 (C(4’)), 89.1 (C(1’)), 67.2 (*dd*, *J* = 11.3, 8.2 Hz, C(5’)), 47.5 (C(2’)), 33.5 (*dd*, *J* = 12.3, 9.1 Hz, C(6’)), 32.5 (*d*, *J* = 4.1 Hz, C(7’)), 6.7 ($(CH_3CH_2)_3Si$), 5.1 (d, *J* = 2.0 Hz, $(CH_3CH_2)_3Si$), 1.8 ($Me_3Si$); $^{19}F$ NMR (282 MHz, $CDCl_3$) δ -134.8 (*dd*, *J* = 164.0, 16.7 Hz, 1F), -145.7 (*d*, *J* = 164.0 Hz, 1F); $ESI^+$-HRMS *m/z* calcd for $C_{28}H_{40}O_5N_3F_2Si_2$ $[(M + H)^+]$ 592.2469, found 592.2467.

### 1-[(3’*S*)-2’-Deoxy-6’-fluoro-5’-on-3’,5’-prop-6’-eno-β-D-ribofuranosyl]-4-*N*-benzoyl-cytosine (13)

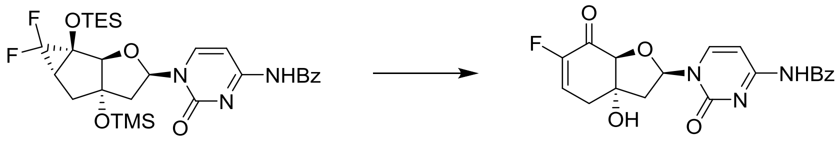


**12β** **13**

The tricyclic sugar **12β** (740 mg, 1.25 mmol) was dissolved in a mixture of dry pyridine (2.4 mL) and dry DCM (12 mL) at rt. The solution was cooled to 0 °C and HF-pyridine (≈70% HF, 0.65 mL, 25.0 mmol, 20 equiv) was added dropwise. The resulting mixture was stirred at 0 °C for 15 min before silica gel (2.86 g) was added. Then it was warmed to rt, and the solvent was removed under reduced pressure. Purification by CC (3% MeOH in DCM) yielded the fluoroenone **13** (440 mg, 1.14 mmol, 91%) as a white foam.

Data for **13**: $R_f$ = 0.33 (10% MeOH in DCM); $^1H$ NMR (400 MHz, DMSO-$d_6$) δ 11.21 (*s*, 1H, N*H*), 8.14 (*d*, *J* = 7.5 Hz, 1H, H-C(6)), 8.03 – 7.96 (*m*, 2H, H-arom), 7.66 – 7.58 (*m*, 1H, H-arom), 7.55 – 7.48 (*m*, 2H, H-arom), 7.35 (*d*, *J* = 7.5 Hz, 1H, H-C(5)), 6.69 (*ddd*, *J* = 13.9, 6.1, 3.1 Hz, 1H, H-C(7’)), 6.12 (*dd*, *J* = 8.5, 5.5 Hz, 1H, H-C(1’)), 5.96 (*s*, 1H, O*H*), 4.60 (*s*, 1H, H-C(4’)), 2.92 (*ddd*, *J* = 19.3, 6.0, 4.4 Hz, 1H, H-C(8’)), 2.80 (*ddd*, *J* = 19.3, 6.6, 3.1 Hz, 1H, H-C(8’)), 2.57 (*dd*, *J* = 13.6, 5.5 Hz, 1H, H-C(2’)), 2.08 (*dd*, *J* = 13.6, 8.5 Hz, 1H, H-C(2’)); $^{13}C$ NMR (101 MHz, DMSO-$d_6$) δ 189.3 (*d*, *J* = 18.6 Hz, C(5’)), 167.2 (*C*ONH), 163.1 (C(4)), 154.3 (C(2)), 151.4 (*d*, *J* = 257.6 Hz, C(6’)), 144.8 (C(6)), 133.1 (C-arom), 132.7, 128.4 (*C*H-arom), 124.5 (*d*, *J* = 14.5 Hz, C(7’)), 95.9 (C(5)), 88.2 (C(1’)), 86.8 (*d*, *J* = 5.8 Hz, C(4’)), 78.4 (*d*, *J* = 1.2 Hz, C(3’)), 42.7 (C(2’)), 31.7 (*d*, *J* = 5.8 Hz (C(8’)); $^{19}F$ NMR (282 MHz, DMSO-$d_6$)

δ -132.6 – -132.8 (*m*, 1F); ESI$^+$-HRMS *m/z* calcd for $C_{19}H_{17}O_5N_3F$ [(M + H)$^+$] 386.1147, found 386.1150.

**1-[(3'*S*,5'*S*)-2'-Deoxy-6'-fluoro-3',5'-prop-6'-eno-β-D-ribofuranosyl]-4-*N*-benzoyl-cytosine (14)**

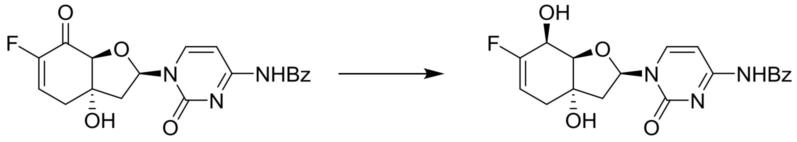


**13** **14**

$CeCl_3$·7 $H_2O$ (458 mg, 1.23 mmol, 1.1 equiv) was added to a suspension of **13** (430 mg, 1.12 mmol) in dry MeOH (12 mL) at rt. The solution was cooled to −78 °C and $NaBH_4$ (51 mg, 1.35 mmol, 1.2 equiv) was added in one portion. After stirring for 20 min at this temperature silica gel (566 mg) was added, and the mixture was warmed to rt. The solvent was removed under reduced pressure. The residue was filtrated over silica gel and washed with a mixture of MeOH (3% → 60%) in EtOAc. The obtained white solid (515 mg, >100%) was used for the next step without further purification.

To a solution of the crude product in DMF (9 mL) was added $Bz_2O$ (443 mg, 1.96 mmol, 1.7 equiv) at rt. After stirring for 7.1 h the solvent was removed under reduced pressure. The residue was purified by CC (5% →10% MeOH in DCM) to afford the diol **14** (407 mg, 1.05 mmol, 94% over two steps) as a white foam.

Data for **14**: $R_f$ = 0.26 (10% MeOH in DCM); $^1$H NMR (400 MHz, DMSO-*d*$_6$) δ 11.27 (*br*, 1H, N*H*), 8.64 (*d*, *J* = 7.5 Hz, 1H, H-C(6)), 8.03 – 7.99 (*m*, 2H, H-arom), 7.65 – 7.56 (*m*, 1H, H-arom), 7.54 – 7.45 (*m*, 2H, H-arom), 7.32 (*d*, *J* = 7.5 Hz, 1H, H-(5)), 6.16 (*dd*, *J* = 8.7, 5.2 Hz, 1H, H-C(1')), 5.92 (*br*, 1H, *H*O-C(5')), 5.48 (*br*, 1H, *H*O-C(3')), 5.34 (*ddd*, *J* = 14.1, 5.2, 3.6 Hz, 1H, H-C(7')), 4.32 (*dd*, *J* = 10.5, 5.3 Hz, 1H, H-C(5')), 4.12 (*d*, *J* = 5.3 Hz, 1H, H-C(4')), 2.60 (*ddd*, *J* = 17.8, 5.7, 3.6 Hz, 1H, H-C(8')), 2.54 – 2.48 (*m*, H-C(2')), 2.33 (*dt*, *J* = 17.8, 5.2 Hz, 1H, H-C(8')), 1.93 (*dd*, *J* = 12.5, 8.7 Hz, 1H, H-C(2')); $^{13}$C NMR (101 MHz, DMSO-*d*$_6$) δ 167.6 (*br*, *C*ONH), 162.9 (C(4)), 158.1 (*d*, *J* = 259.7 Hz, C(6')), 154.4 (C(2)), 145.7 (C(6)), 133.3 (C-arom), 132.7, 128.5, 128.4 (*C*H-arom), 102.4 (*d*, *J* = 15.8 Hz, C(7')), 95.6 (C(5)), 86.4 – 85.7 (*m*, C(1'), C(4')), 75.6 (C(3')), 64.4 (*d*, *J* = 25.9 Hz, C(5')), 45.5 (C(2')), 33.3 (*d*, *J*

= 8.1 Hz, C(8')); $^{19}$F NMR (282 MHz, DMSO-$d_6$) δ -113.5 – -113.7 (*m*, 1F); ESI$^+$-HRMS *m/z* calcd for $C_{19}H_{19}O_5N_3F$ [(M + H)$^+$] 388.1303, found 388.1305.

**1-{(3'*S*,5'*S*)-3'-*O*-[(2-Cyanoethoxy)-diisopropylaminophosphanyl]-2'-deoxy-6'-fluoro-5'-*O*-[(4,4'-dimethyoxytriphenyl)methyl]-3',5'-prop-6'-eno-β-D-ribofuranosyl}-4-*N*-benzoyl-cytosine (15)**

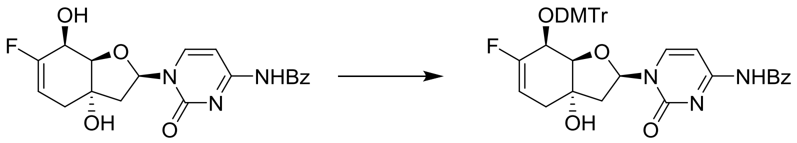


**14** **15**

To a suspension of AgOTf (1.54 g, 6 mmol) in dry DCM (1 mL) was added a solution of DMTr-Cl (2.03 g, 6 mmol) in dry DCM (5 mL). The resulting mixture was stirred 1 h at rt. Four equivalent portions of the supernatant (2.24 mL, 2.24 mmol, 2.7 equiv) were then added to a solution of the nucleoside **14** (321 mg, 0.83 mmol) in a mixture of dry pyridine (3.6 mL) and dry DCM (1.8 mL) over a period of 97 min. Afterwards the reaction mixture was stirred 19.5 h at rt before it was diluted with EtOAc (50 mL) and washed with sat. $NaHCO_3$ sol. (2 × 50 mL). The combined aqueous phase was extracted with EtOAc (3 × 100 mL). The combined organic phases were dried ($MgSO_4$), concentrated and purified by CC (1% → 4% MeOH in DCM + 0.5% $Et_3N$). The DMTr-protected nucleoside **15** (251 mg, 0.36 mmol, 44%) was obtained as white foam.

Data for **15**: $R_f$ = 0.36 (5% MeOH in DCM); $^1$H NMR (400 MHz, DMSO-$d_6$) δ 11.25 (*br*, 1H, N*H*), 8.04 – 7.97 (*m*, 3H, H-C(6), H-arom), 7.66 – 7.60 (*m*, 1H, H-arom), 7.55 – 7.49 (*m*, 2H, H-arom), 7.48 – 7.42 (*m*, 2H, H-arom), 7.38 – 7.28 (*m*, 6H, H-arom), 7.27 – 7.19 (*m*, 1H, H-arom), 7.04 (*d*, *J* = 7.4 Hz, 1H, H-C(5)), 6.90 (*dd*, *J* = 8.9, 3.8 Hz, 4H, H-arom), 6.03 (*dd*, *J* = 8.2, 5.6 Hz, 1H, H-C(1')), 5.42 (*s*, 1H, O*H*), 5.32 (*dt*, *J* = 12.6, 5.3 Hz, 1H, H-C(7')), 4.20 (*t*, *J* = 4.7 Hz, 1H, H-C(5')), 3.74 (*s*, 3H, *Me*O), 3.73 (s, 3H, *Me*O), 3.60 (*dd*, *J* = 4.7, 3.0 Hz, 1H, H-C(4')), 2.57 (*dt*, *J* = 16.2, 4.7 Hz, 1H, H-C(8')), 2.51 – 2.45 (*m*, 1H, H-C(2')), 2.20 (*dt*, *J* = 16.2, 5.0 Hz, 1H, H-C(8')), 1.91 (*dd*, *J* = 13.2, 8.2 Hz, 1H, H-C(2')); $^{13}$C NMR (101 MHz, DMSO-$d_6$) δ 167.3 (*br*, *C*ONH), 163.0 (*br*, C(4)), 158.3, 158.3 (C-arom), 157.7 (*d*, *J* = 267.4 Hz, C(6')), 154.0 (*br*, C(2)), 145.0 (C-arom), 144.2 – 143.8 (*m*, C(6)), 135.7, 135.1, 133.1 (C-arom), 132.7, 130.3, 130.1, 128.4, 127.9, 127.7, 126.8 , 113.1, 113.1 (*C*H-arom), 102.3 (*d*, *J* = 14.0 Hz, C(7')), 96.2 – 95.8 (*m*, C(5)), 87.9 (*C*(Ph)$_3$), 86.9 (*d*, *J* = 4.7 Hz, C(4')), 85.0

(C(1')), 76.9 (C(3')), 67.2 (*d*, *J* = 22.8 Hz, C(5')), 55.0, 55.0 (*Me*O-DMTr), 47.1 (C(2')), 33.4 (*d*, *J* = 7.2 Hz, C(8')); $^{19}$F NMR (376 MHz, DMSO-$d_6$) δ -110.9 (*br*, 1F); ESI$^+$-HRMS *m/z* calcd for $C_{40}H_{37}O_7N_3F$ [(M + H)$^+$] 690.2610, found 690.2598.

**1-{(3'*S*,5'*S*)-3'-*O*-[(2-Cyanoethoxy)-diisopropylaminophosphanyl]-2'-deoxy-6'-fluoro-5'-*O*-[(4,4'-dimethyoxytriphenyl)methyl]-3',5'-prop-6'-eno-β-D-ribofuranosyl}-4-*N*-benzoyl-cytosine (16)**

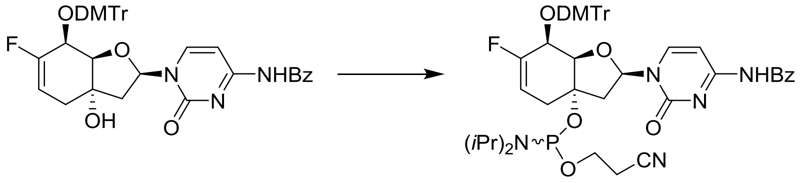


**15** **16**

The DMTr-protected nucleoside **15** (215 mg, 0.31 mmol) was dissolved in dry THF (2.5 mL) at rt. DIPEA (0.22 mL, 1.26 mmol, 4.1 equiv) and CEP-Cl (0.14 mL, 0.63 mmol, 2.0 equiv) were added and the mixture was stirred 76 min at rt. Then the solvent was removed under reduced pressure and the residue was purified by CC (hex/EtOAc 1:1 + 0.5% $Et_3N$). The phosphoramidite **16** (118 mg, 0.13 mmol, 43%) was obtained as a mixture of two isomers.

Data for **16**: $R_f$ = 0.46 & 0.37 (hex/EtOAc 1:5); $^1$H NMR (400 MHz, DMSO-$d_6$) δ 11.27 (*br*, 2H, 2x N*H*), 8.04 – 7.97 (*m*, 6H, 2x H-C(6), H-arom), 7.68 – 7.59 (*m*, 2H, H-arom), 7.55 – 7.49 (*m*, 4H, H-arom), 7.49 – 7.43 (*m*, 4H, H-arom), 7.38 – 7.27 (*m*, 12H, H-arom), 7.26 – 7.19 (*m*, 2H, H-arom), 7.19 – 7.03 (*m*, 2H, 2x H-C(5)), 6.94 – 6.86 (*m*, 8H, H-arom), 6.04 (*dd*, *J* = 7.6, 5.9 Hz, 1H, H-C(1')), 5.97 (*dd*, *J* = 7.5, 6.0 Hz, 1H, H-C(1')), 5.43 – 5.29 (*m*, 2H, 2x H-C(7')), 4.32 (*t*, *J* = 4.3 Hz, 1H, H-C(5')), 4.24 (*t*, *J* = 4.7 Hz, 1H, H-C(5')), 3.77 – 3.71 (*m*, 1H, H-C(4')), 3.74 (*s*, 6H, 2x *Me*O), 3.73 (*s*, 6H, 2x *Me*O), 3.70 – 3.57 (*m*, 4H, 2x OC$H_2$C$H_2$CN), 3.57 – 3.45 (*m*, 5H, H-C(4'), 2x ($Me_2$C*H*)$_2$N), 2.84 – 2.74 (*m*, 2H, H-C(2')) 2.75 (*t*, *J* = 5.9 Hz, 2H, OC$H_2$C*H*$_2$CN), 2.70 (*t*, *J* = 5.9 Hz, 2H, OC$H_2$C*H*$_2$CN), 2.70 – 2.65 (*m*, 1H, H-C(8')), 2.65 – 2.56 (*m*, 3H, 3x H-C(8')), 2.05 (*td*, *J* = 13.0, 7.7 Hz, 2H, H-C(2')), 1.14 – 1.01 (*m*, 24H, (*Me*$_2$CH)$_2$N); $^{13}$C NMR (101 MHz, DMSO-$d_6$) δ 167.2 (*br*, *C*ONH), 163.7 (C(4)), 158.4, 158.3 (C-arom), 157.6 (*d*, *J* = 267.0 Hz, C(6')), 157.5 (*d*, *J* = 267.0 Hz, C(6')), 154.0 (*br*, C(2)), 145.0, 145.0 (C-arom), 144.3 – 143.5 (*m*, C(6)), 135.7, 135.6, 135.0, 134.9 (C-arom), 133.1 (*br*, C-arom), 132.7, 130.4, 130.3, 130.1, 128.4, 128.4, 127.9, 127.8, 126.9 (*C*H-arom), 119.0, 119.0 (*C*N), 113.1 (*C*H-arom), 102.2 (*d*, *J* = 14.6 Hz, C(7')), 101.9 (*d*, *J* = 15.1 Hz, C(7')), 96.4 – 96.0 (*m*, C(5)), 88.1, 87.9 (*C*(Ph)$_3$), 86.0 – 85.6 (*m*, C(4')), 85.4 – 85.2 (*m*,

C(4')), 84.9, 84.7 (C(1')), 81.9 (*d*, *J* = 3.5 Hz, C(3')), 81.8 (*d*, *J* = 2.5 Hz, C(3')), 66.9 (*d*, *J* = 23.0 Hz, C(5')), 57.9 (*d*, *J* = 17.0 Hz, O$CH_2CH_2$CN), 57.8 (*d*, *J* = 16.5 Hz, O$CH_2CH_2$CN), 55.0, 55.0 (*Me*O-DMTr), 45.8 – 45.4 (*m*, C(2')),42.9, 42.8, 42.7, 42,7 (($Me_2$*C*H)$_2$N), 32.0 – 31.7 (*m*, C(8')), 24.3, 24.2, 24.1, 24.0, 24.0, 23.9, 23.9 ((*Me*$_2$CH)$_2$N), 19.7, 19.7 (O$CH_2$*C*$H_2$CN); $^{19}$F NMR (376 MHz, DMSO-$d_6$) δ -110.5 (*br*, 1F), -111.1 (*br*, 1F); $^{31}$P NMR (162 MHz, DMSO-$d_6$) δ 140.0, 139.9; ESI$^-$-HRMS *m/z* calcd for $C_{49}H_{52}O_8N_5FP$ [(M - H)$^+$] 888.3532, found 888.3545.

## 9. NMR spectra of the new synthesized compounds

**(1*S*,2*R*,4*S*,6*S*,8*S*)-2-[(Triethylsilyl)oxy]-3,3-difluroro-8-methoxy-6-[(trimethylsilyl)oxy]-9-oxatricyclo[4.3.0$^{1,6}$.0$^{2,4}$]nonane (3α)**

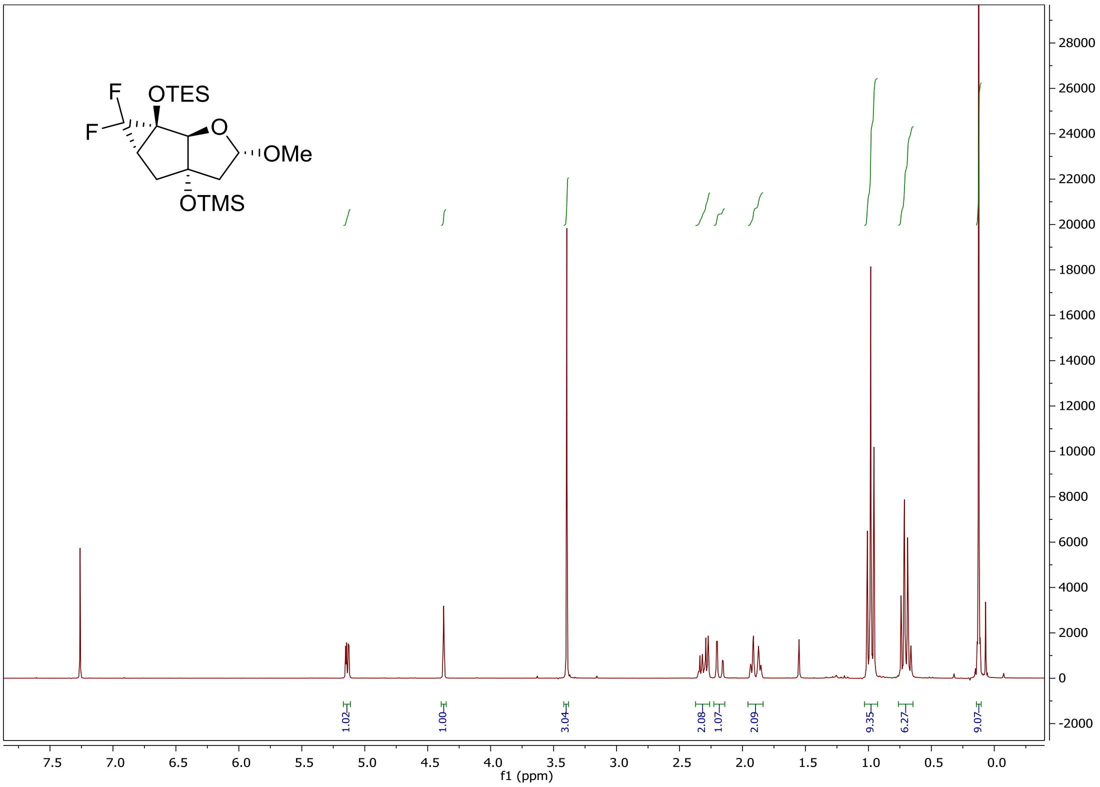


**Figure S9:** $^1$H NMR (300 MHz, $CDCl_3$) spectra of **3α**.

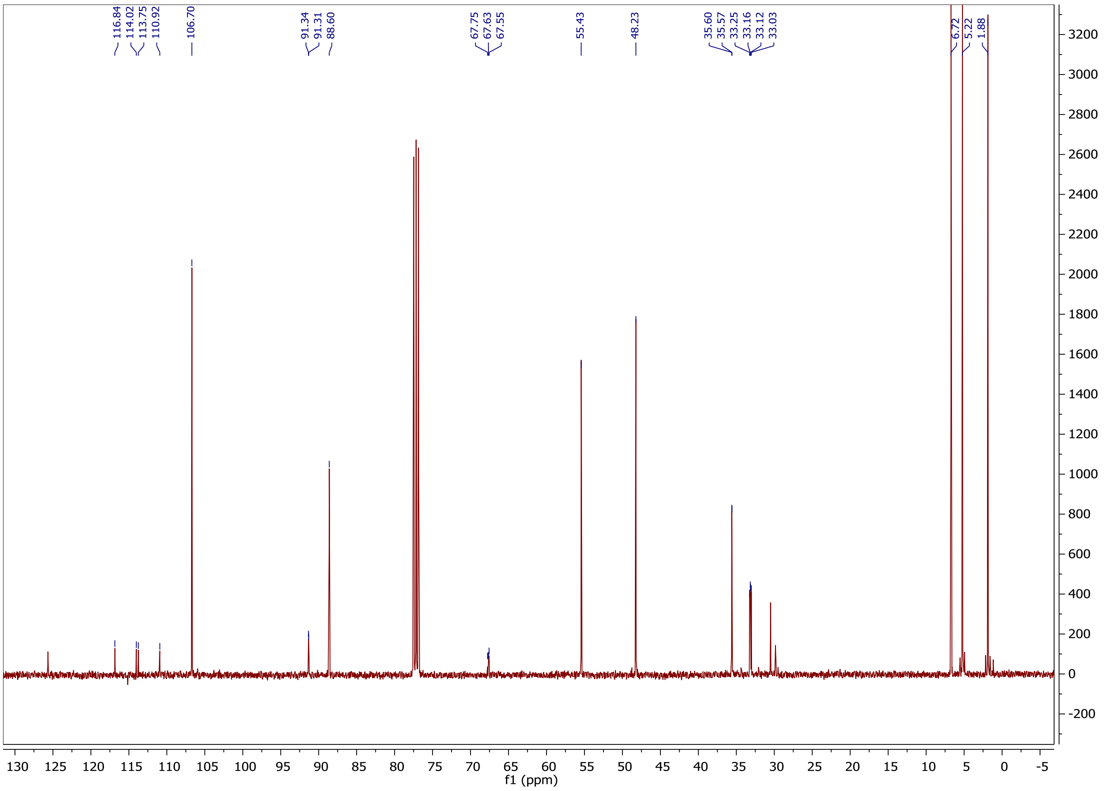


**Figure S10:** $^{13}$C NMR (101 MHz, $CDCl_3$) spectra of **3**α.

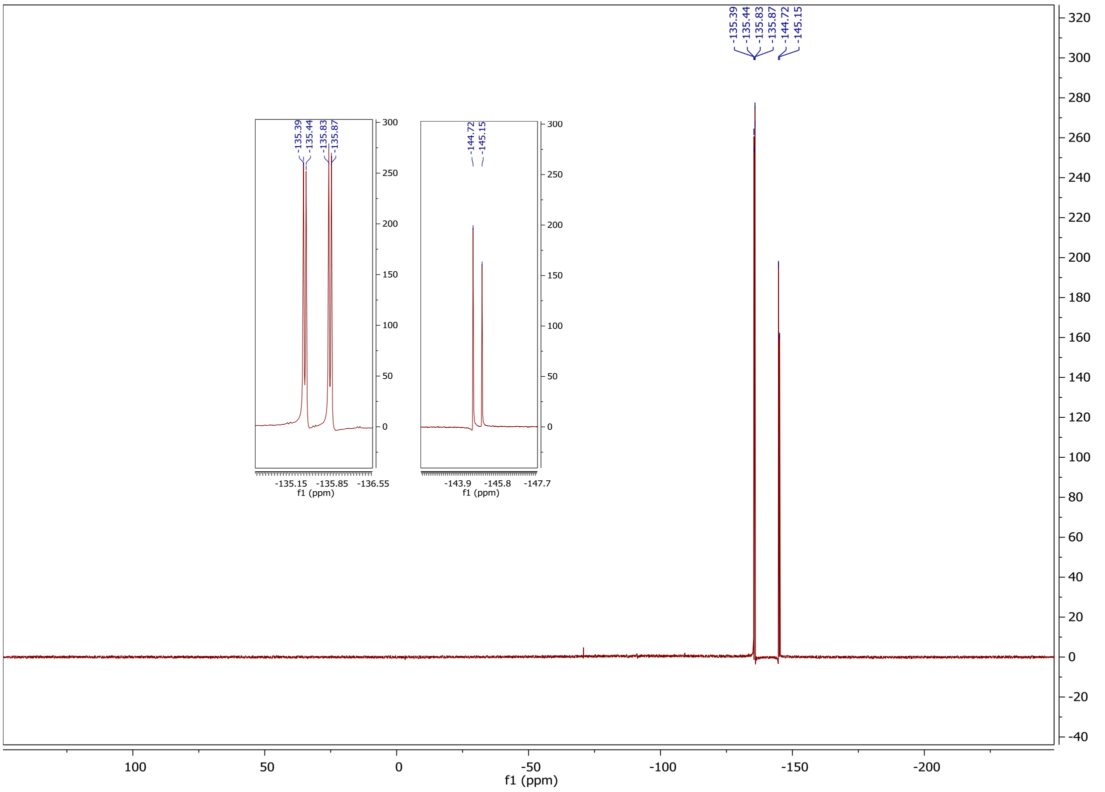


**Figure S11:** $^{19}$F NMR (376 MHz, $CDCl_3$) spectra of **3**α.

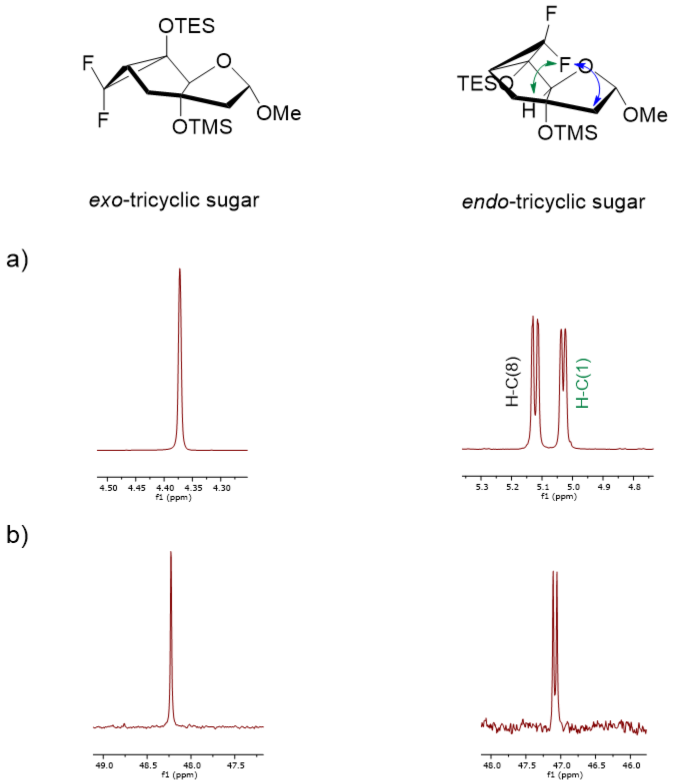


**Figure S12:** NMR-signals of a) the H-C(1) atom (400 MHz, $CDCl_3$) and b) the C(7) atom (101 MHz, $CDCl_3$, proton decoupled) of the *exo*-tricyclic sugar **3α** (left column) and its *endo*-isomer (right column).

**(1*S*,2*R*,4*S*,6*S*,8*R*)-2-[(Triethylsilyl)oxy]-3,3-difluroro-8-methoxy-6-[(trimethylsilyl)oxy]-9-oxatricyclo[4.3.0$^{1,6}$.0$^{2,4}$]nonane (3β)**

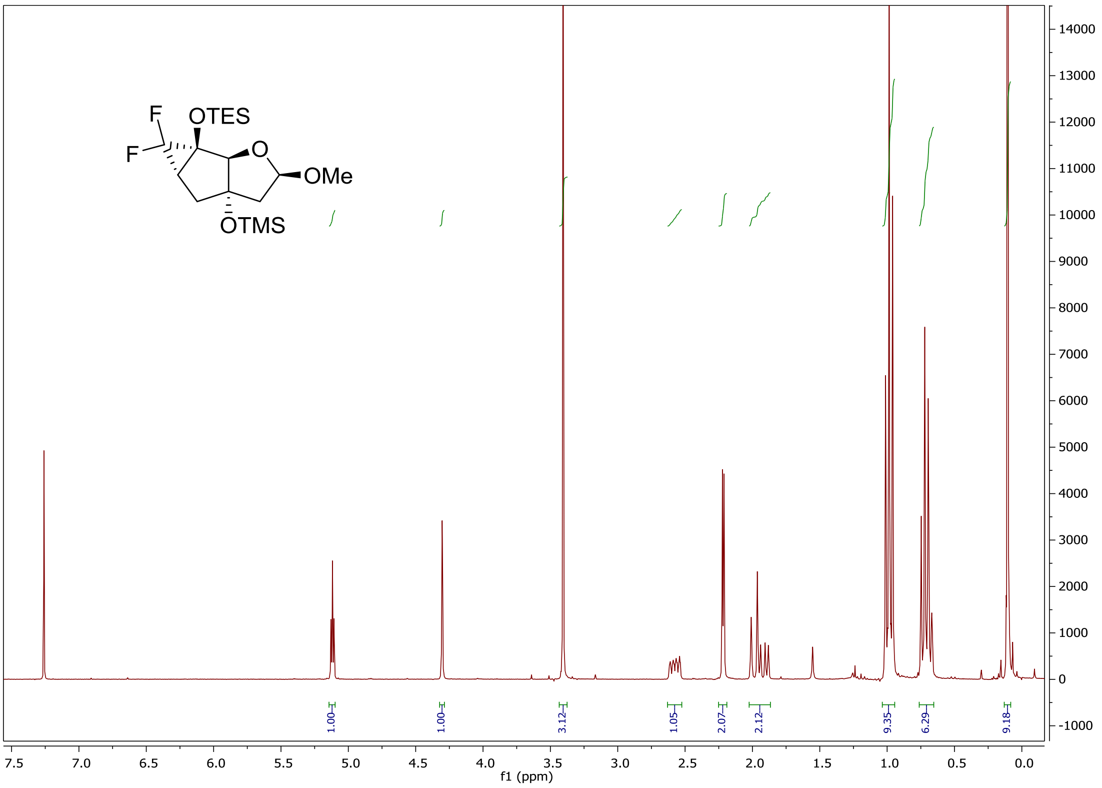


**Figure S13:** $^{1}$H NMR (300 MHz, $CDCl_3$) spectra of **3β**.

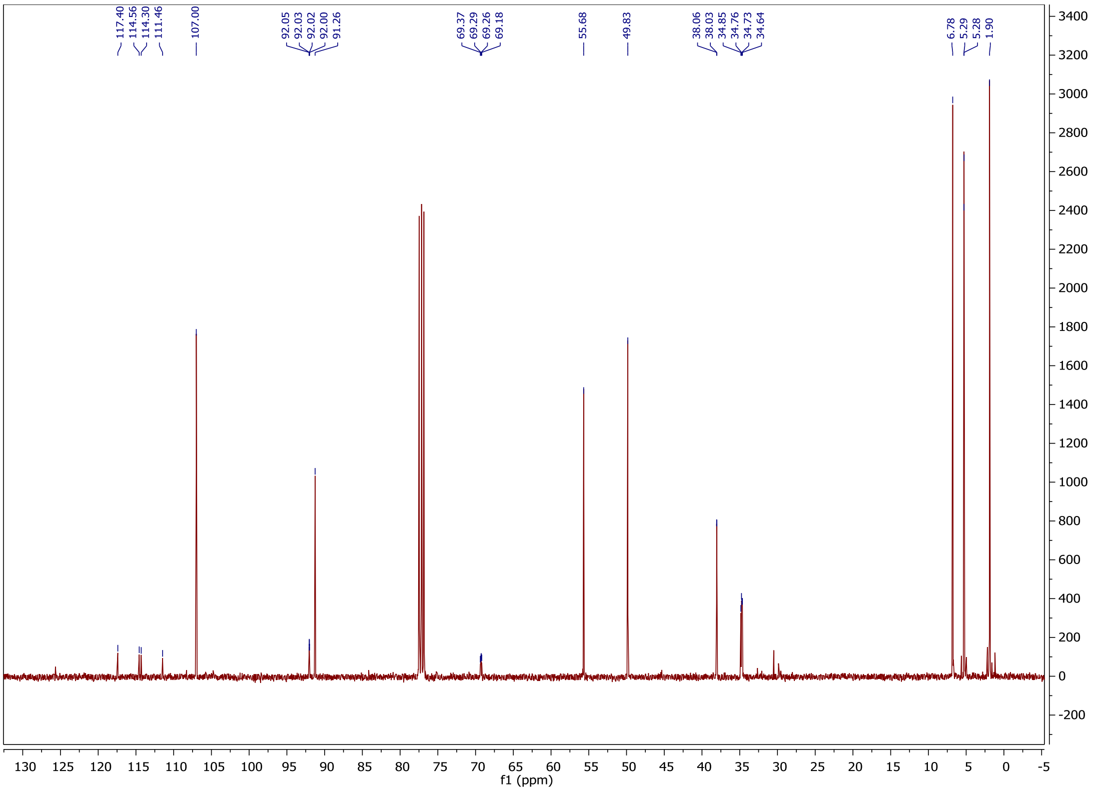


**Figure S14:** $^{13}$C NMR (101 MHz, $CDCl_3$) spectra of **3β**.

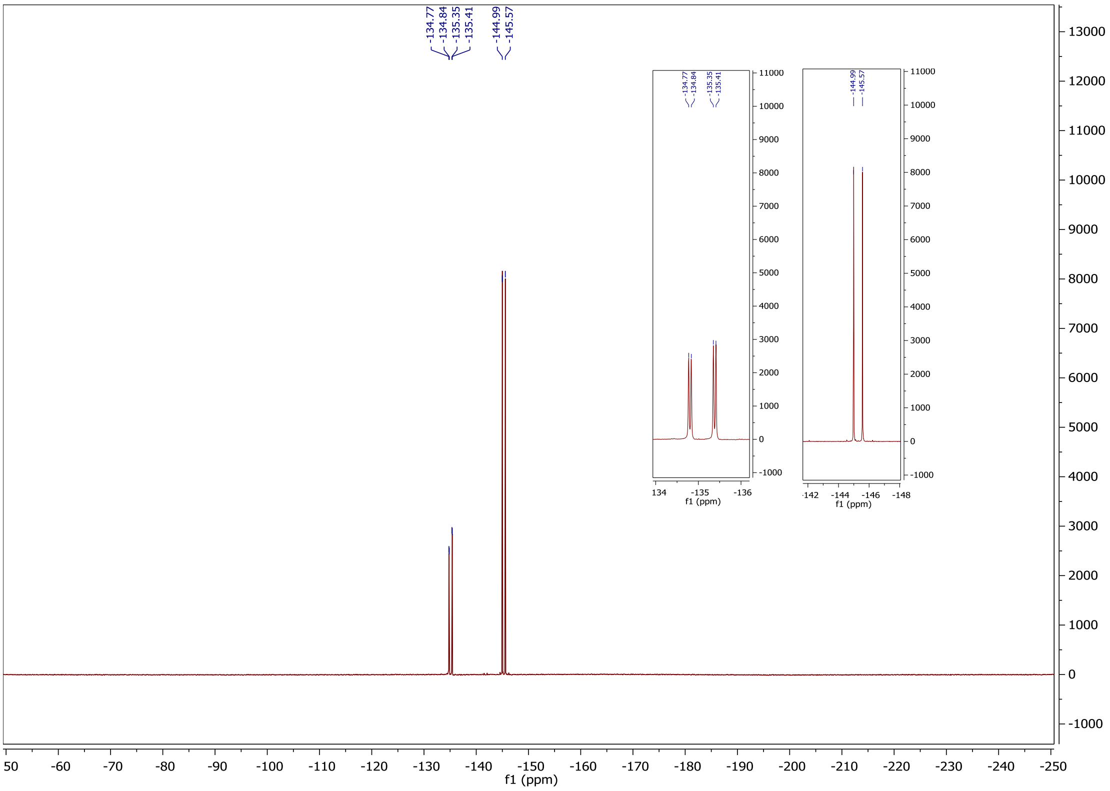

**Figure S15:** $^{19}$F NMR (282 MHz, CDCl3) spectra of **3β**.

## (1*S*,2*R*,4*S*,6*S*)-2-[(Triethylsilyl)oxy]-3,3-difluroro-6-[(trimethylsilyl)oxy]-9-oxatricyclo-[4.3.0$^{1,6}$.0$^{2,4}$]non-7-ene (4)

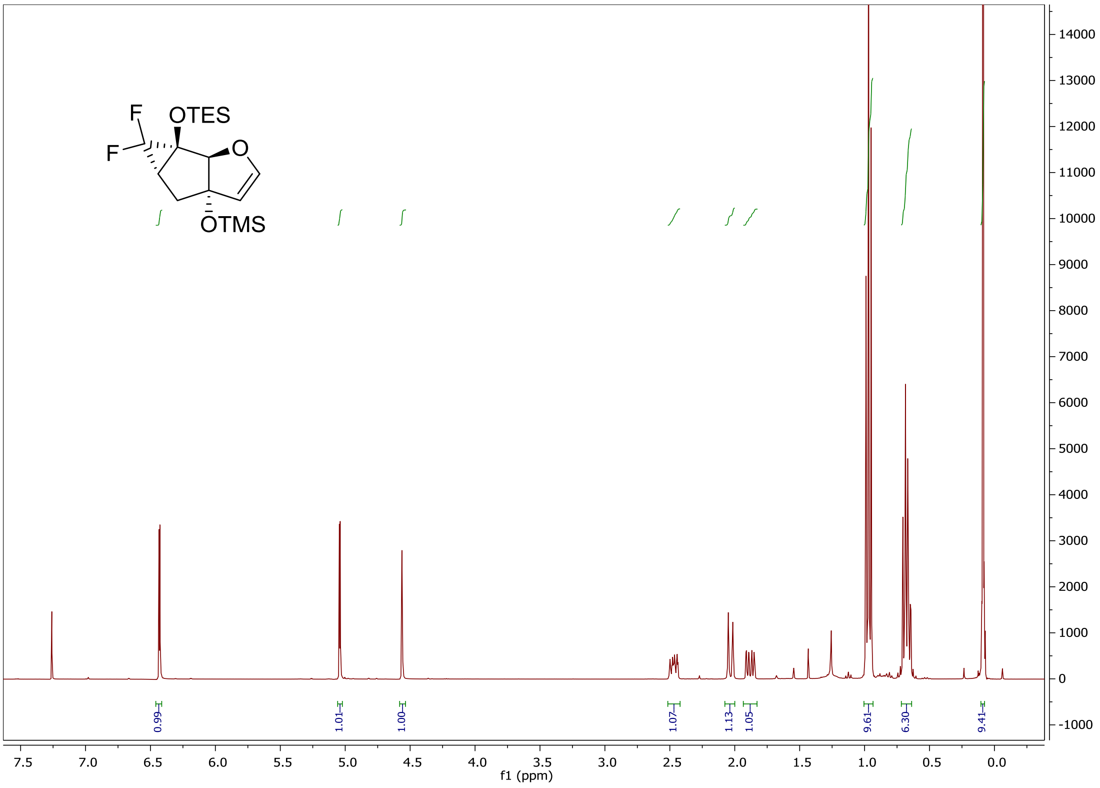


**Figure S16:** $^1$H NMR (400 MHz, $CDCl_3$) spectra of **4**.

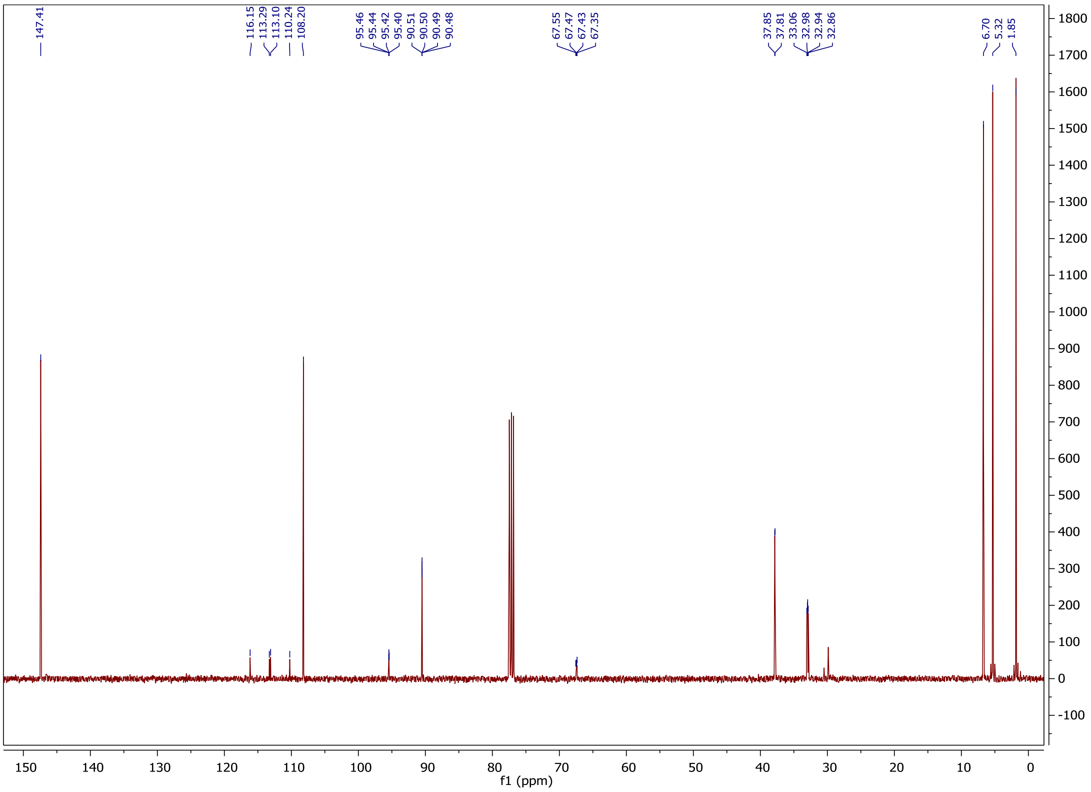


**Figure S17:** $^{13}C$ NMR (101 MHz, $CDCl_3$) spectra of **4**.

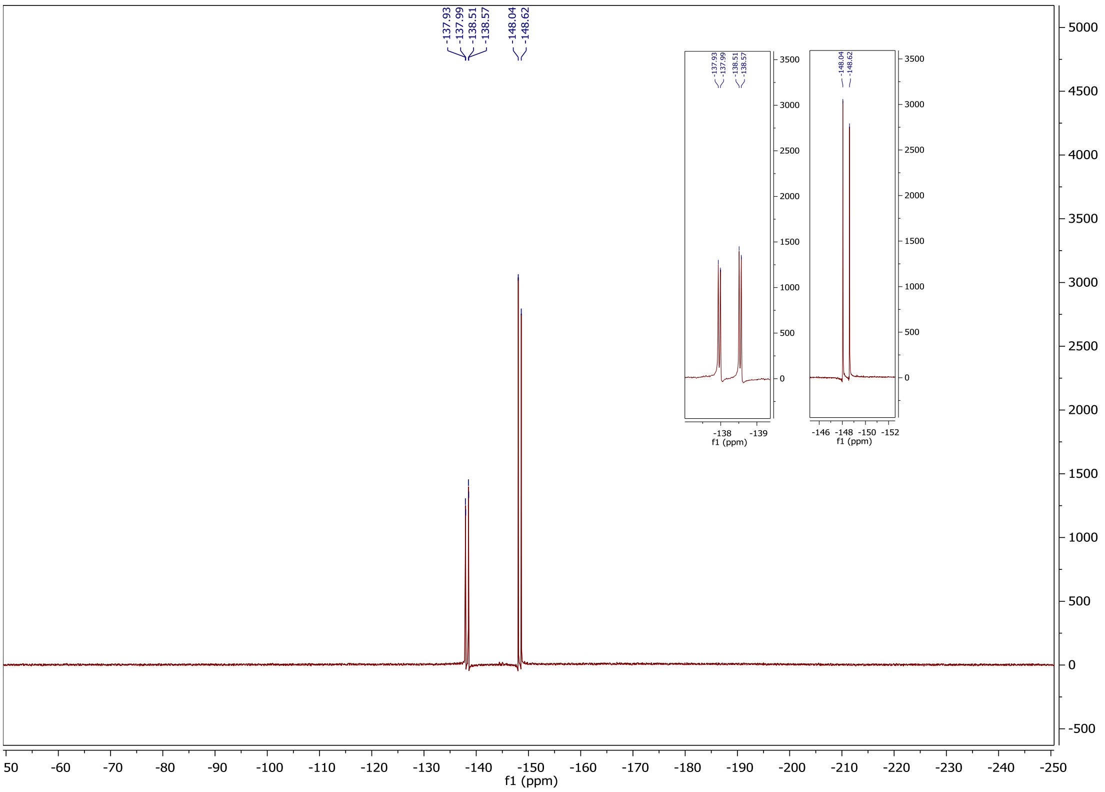


**Figure S18:** $^{19}F$ NMR (282 MHz, $CDCl_3$) spectra of **4**.

## (1*S*,2*R*,4*S*,6*S*,8*R* and *S*)-2-[(Triethylsilyl)oxy]-3,3-difluroro-8-hydroxy-6-[(trimethylsilyl)oxy]-9-oxatricyclo[4.3.0$^{1,6}$.0$^{2,4}$]nonane (5α/β)

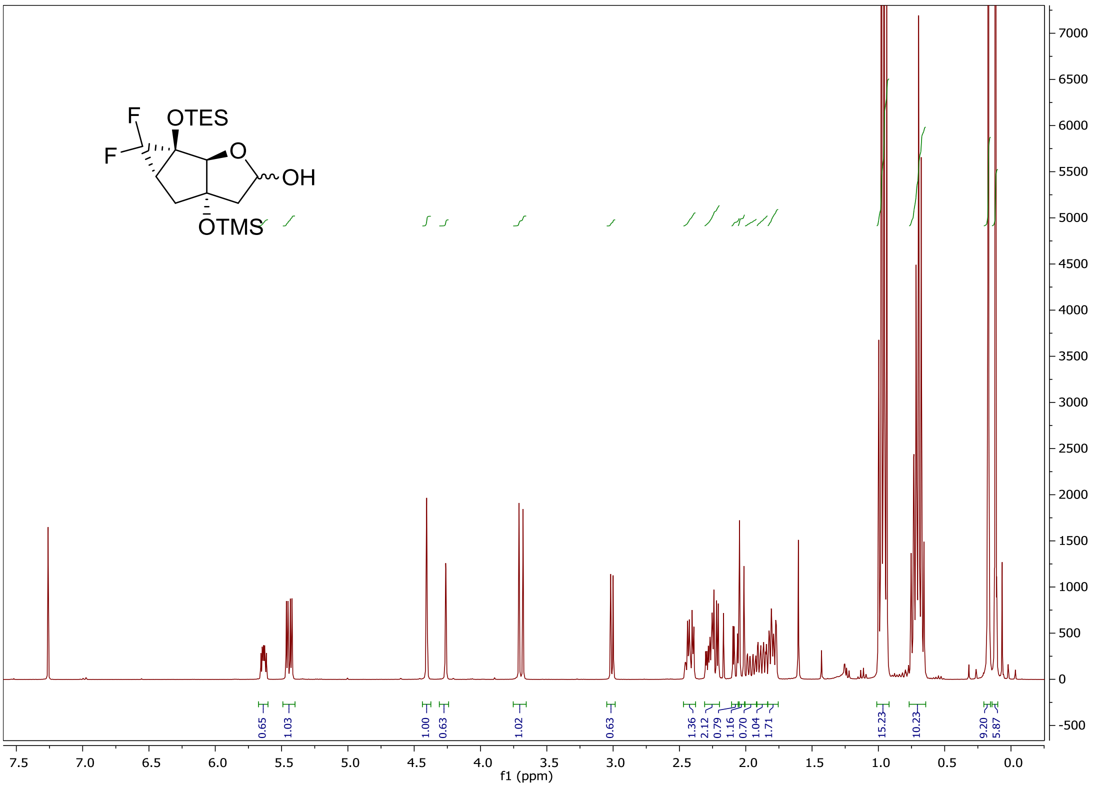


**Figure S19:** $^{1}H$ NMR (400 MHz, $CDCl_3$) spectra of **5α/β**.

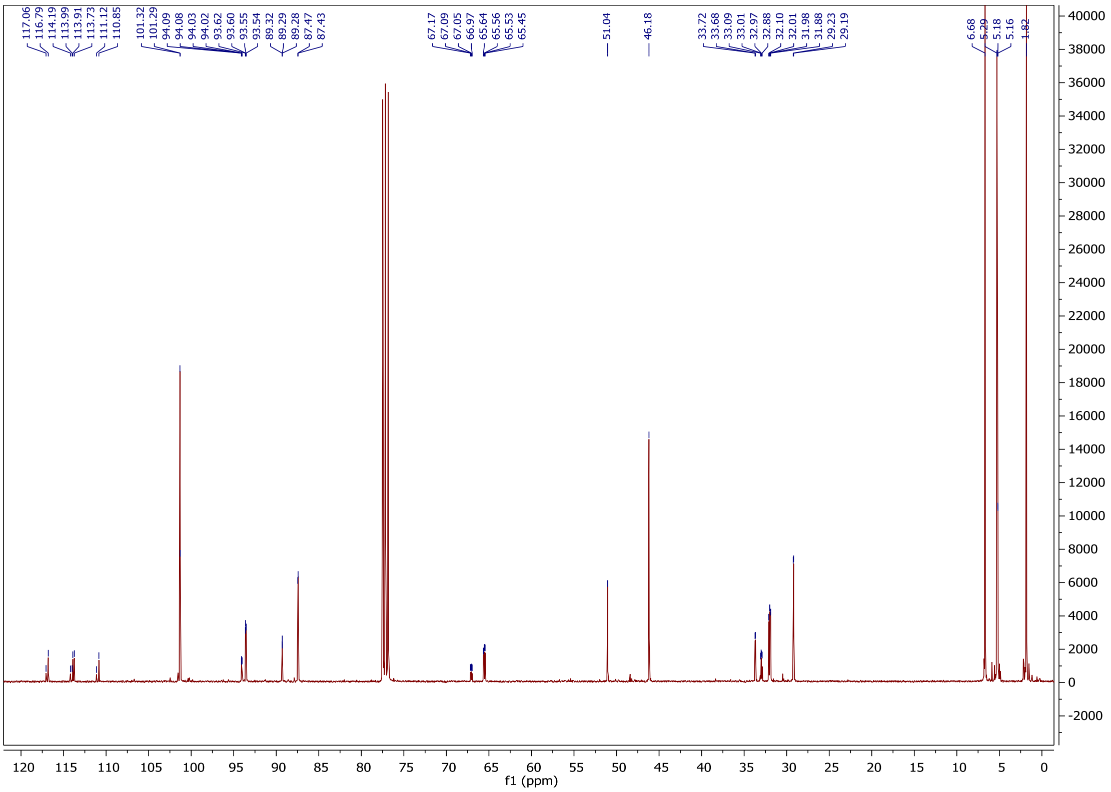


**Figure S20:** $^{13}C$ NMR (101 MHz, $CDCl_3$) spectra of **5α/β**.

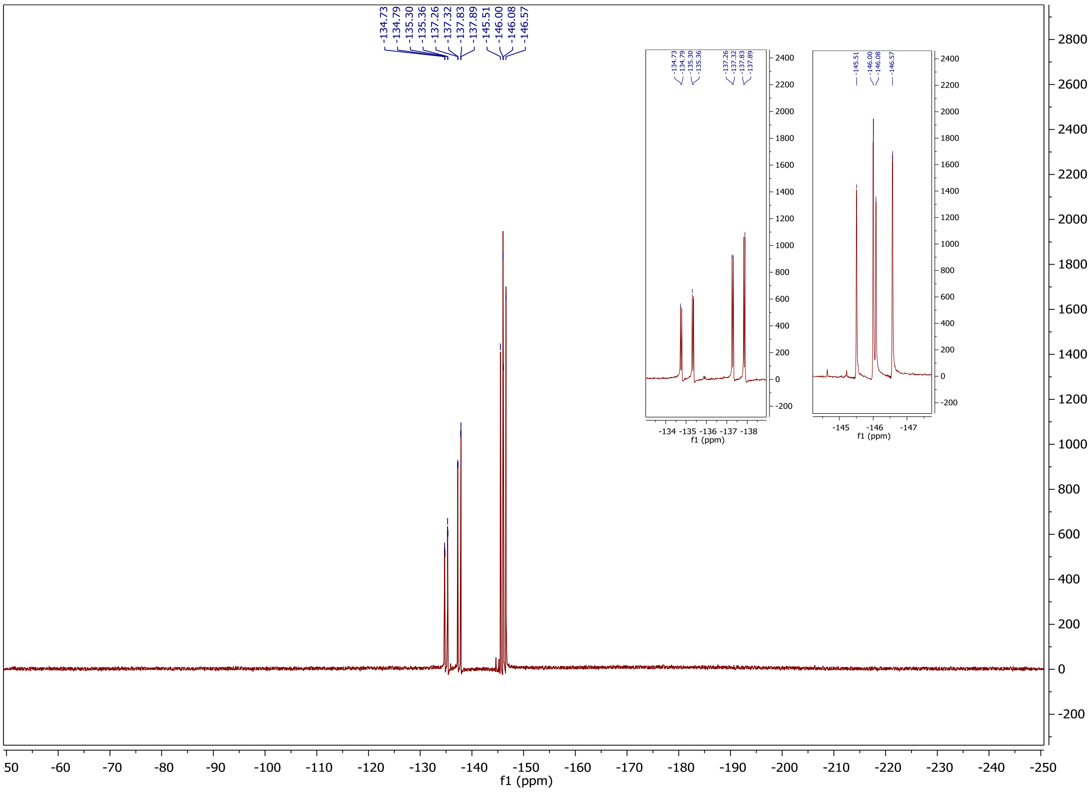

**Figure S21:** $^{19}$F NMR (282 MHz, $CDCl_3$) spectra of **5α/β**.

## 1-[(3'*S*,5'*R*,6'*S*)-2'-Deoxy-3',5'-ethano-5'-*O*-triethylsilyl-8',8'-dilfluoro-5',6'-methano-3'-*O*-trimethylsilyl-α,β-D-ribofuranosyl]thymine (6α/β)

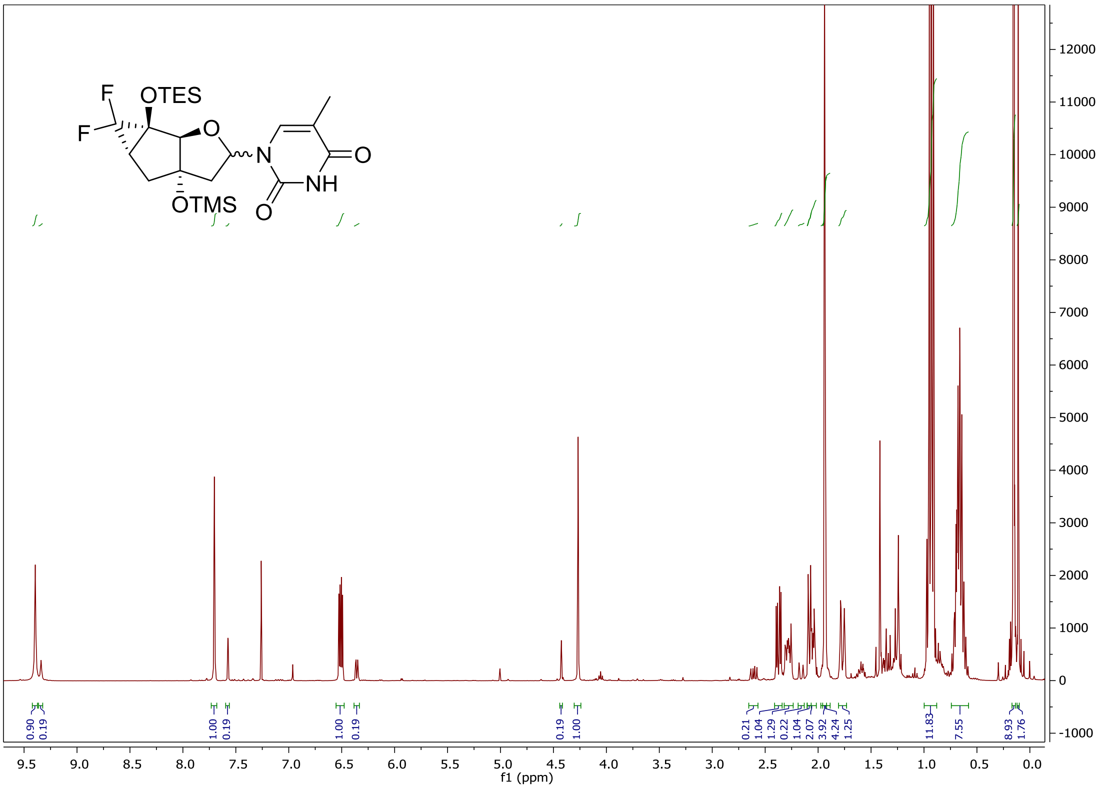


**Figure S22:** $^{1}$H NMR (400 MHz, $CDCl_3$) spectra of **6α/β**.

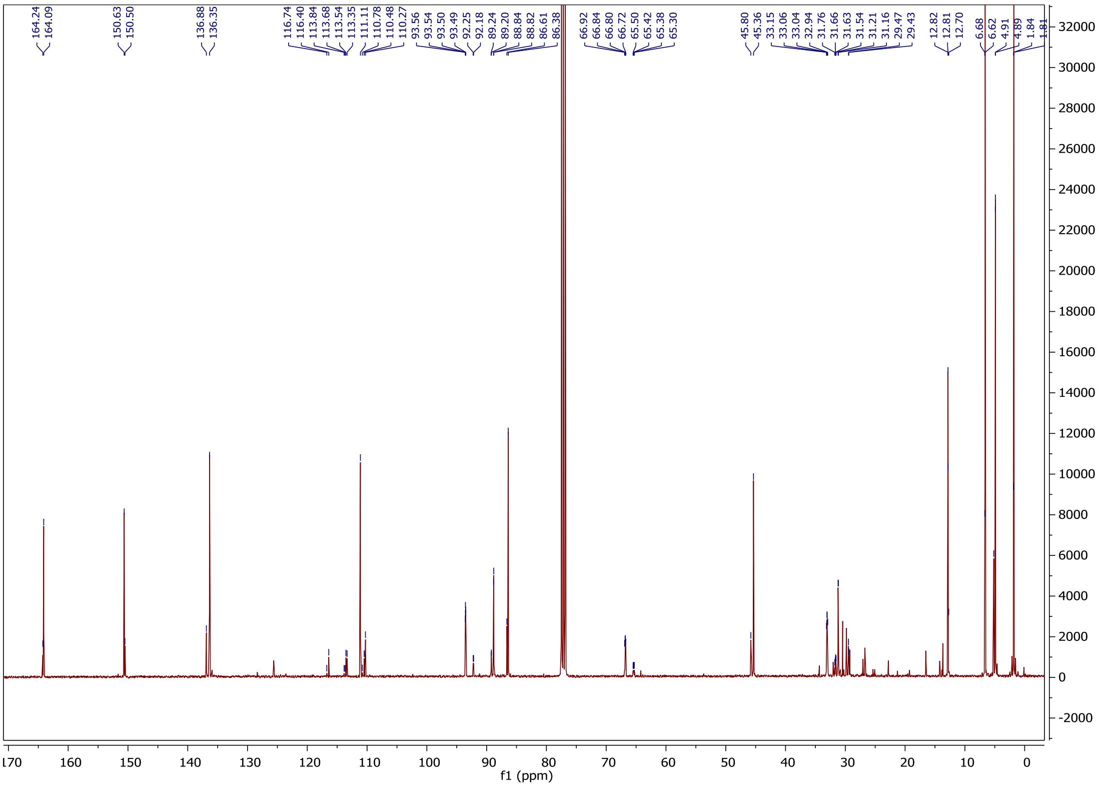

**Figure S23:** $^{13}$C NMR (101 MHz, $CDCl_3$) spectra of **6α/β**.

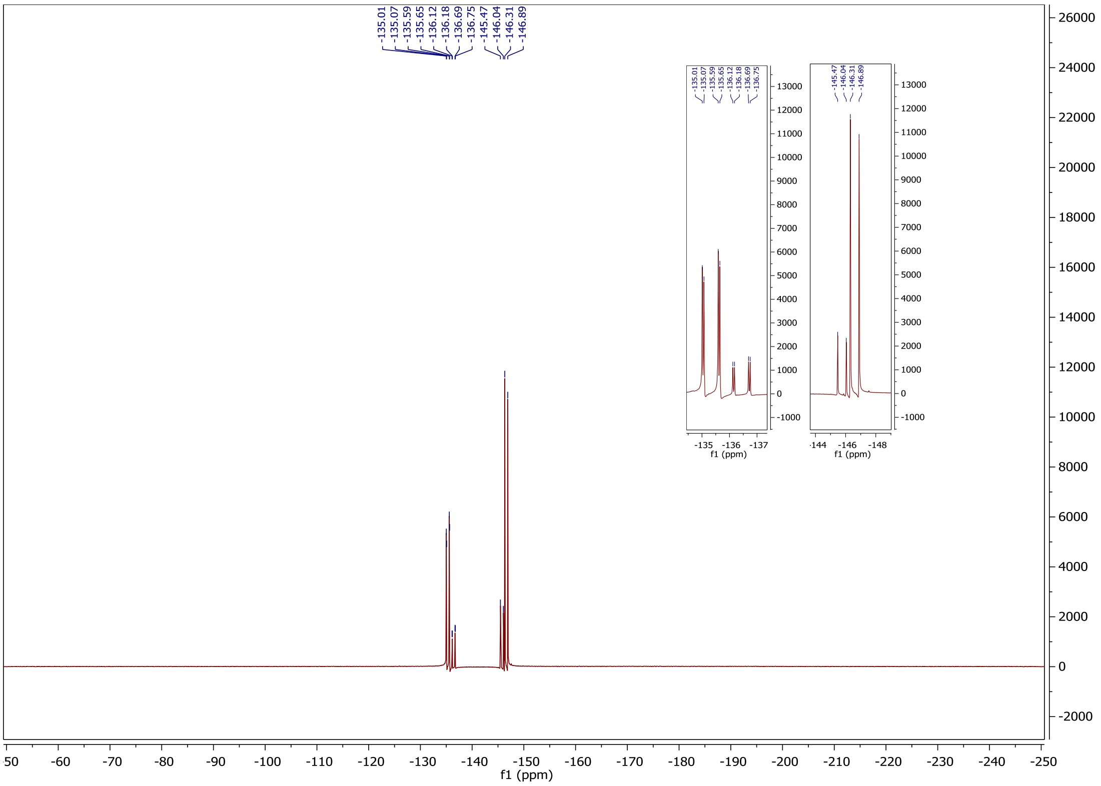

**Figure S24:** $^{19}$F NMR (282 MHz, $CDCl_3$) spectra of **6α/β**.

**1-[(3'*S*)-2'-Deoxy-6'-fluoro-3',5'-prop-6'-en-5'-on-β-D-ribofuranosyl]thymine (7β)**

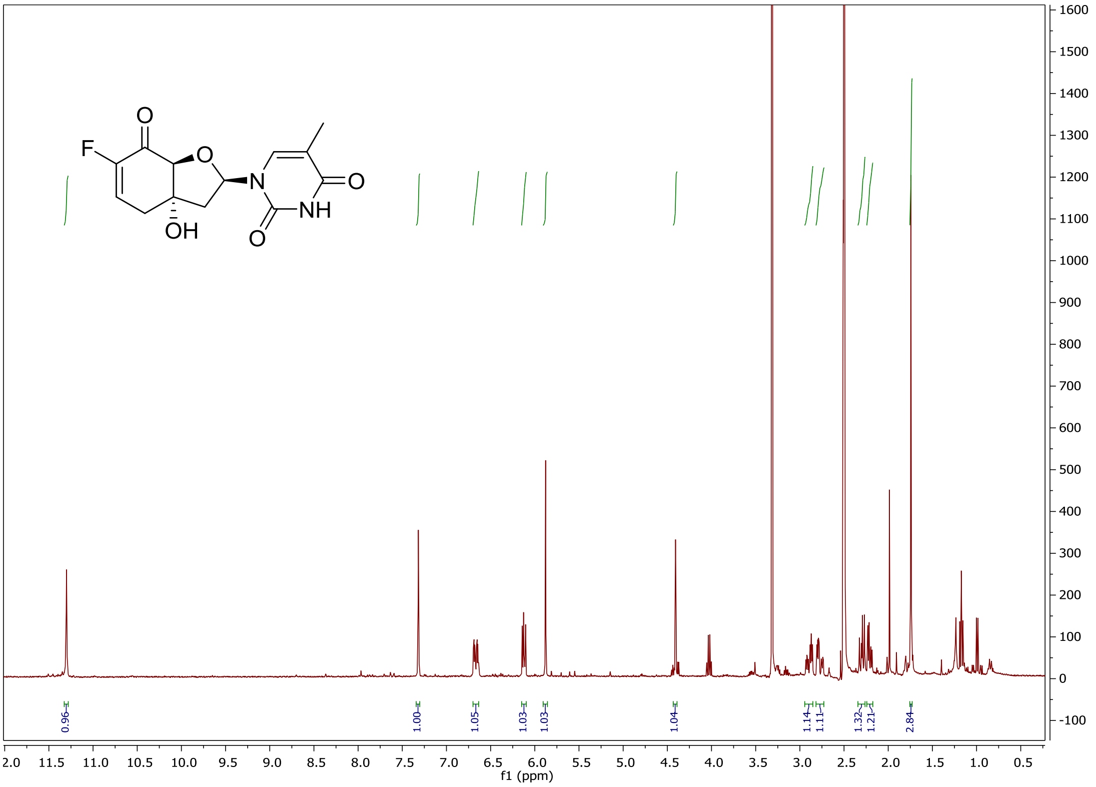


**Figure S25:** $^{1}$H NMR (400 MHz, DMSO-$d_6$) spectra of **7β**.

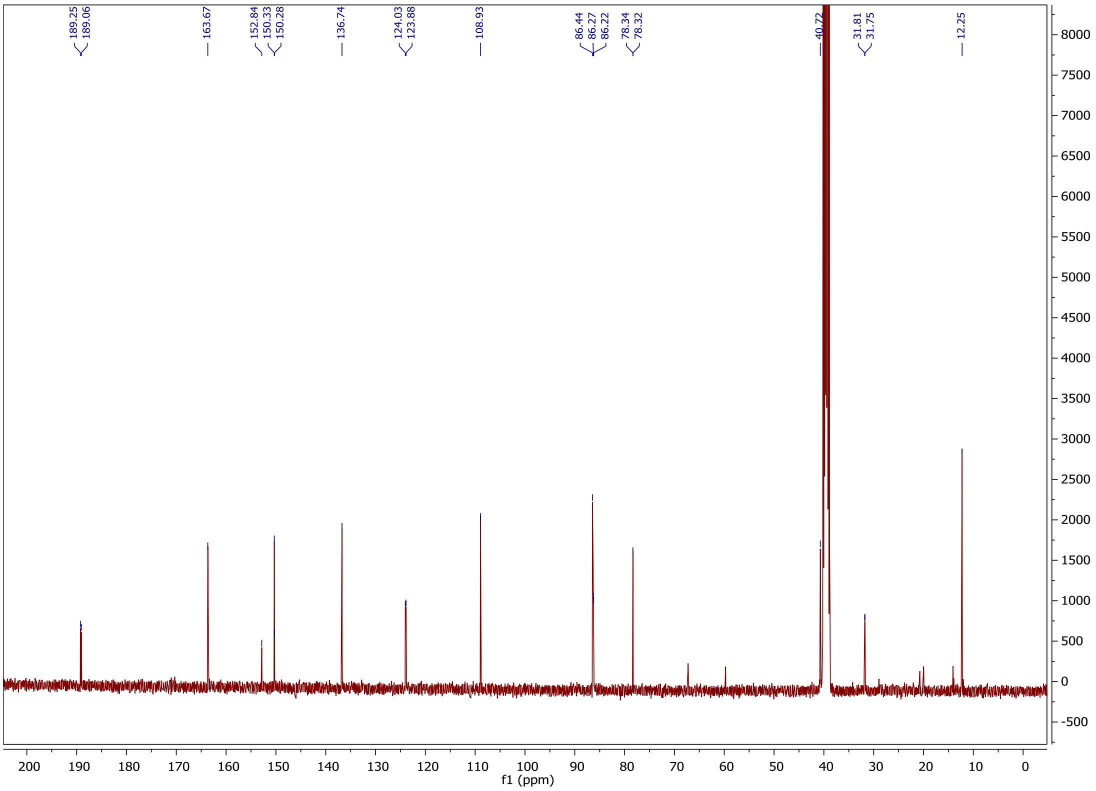


**Figure S26:** $^{13}$C NMR (101 MHz, DMSO-$d_6$) spectra of **7β**.

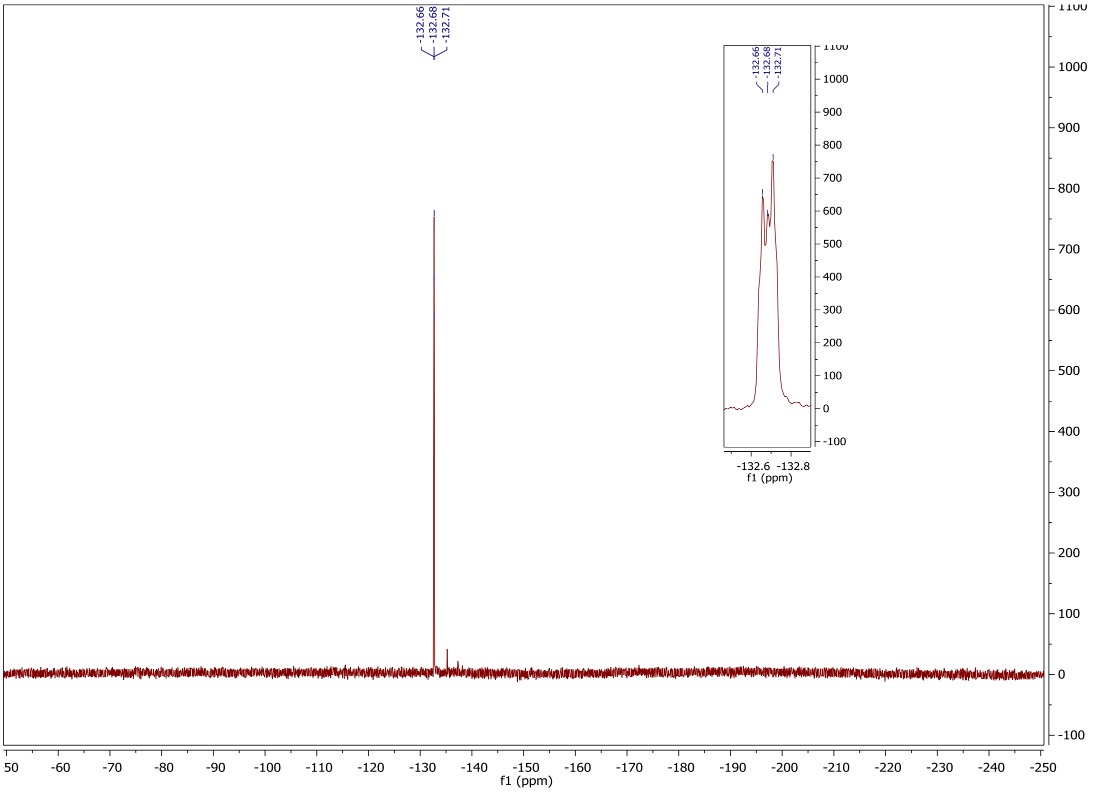


**Figure S27:** $^{19}$F NMR (282 MHz, DMSO-$d_6$) spectra of **7β**.

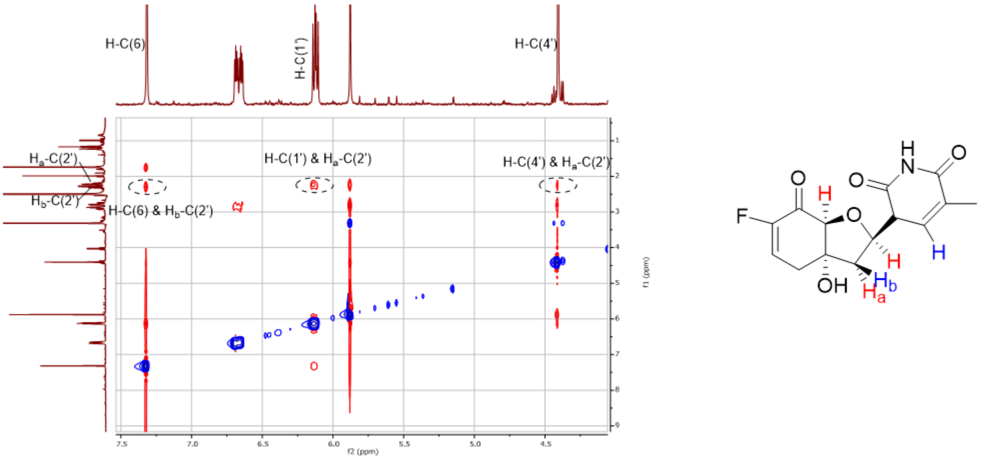


**Figure S28:** $^{1}$H,$^{1}$H-ROESY spectrum (400 MHz, DMSO-$d_6$) of **7β**.

## 1-[(3’*S*,5’*S*)-2’-Deoxy-6’-fluoro-3’,5’-prop-6’-eno-β-D-ribofuranosyl]thymine (8)

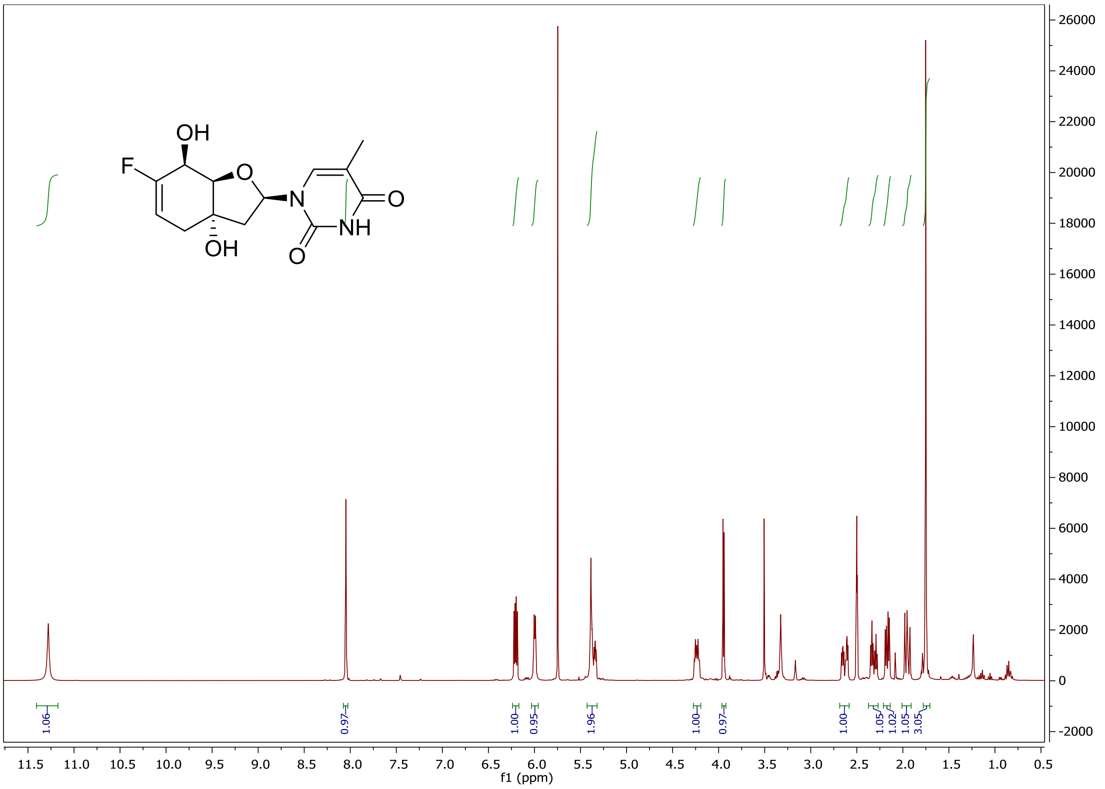

**Figure S29:** $^{1}$H NMR (400 MHz, DMSO-$d_6$) spectra of **8**.

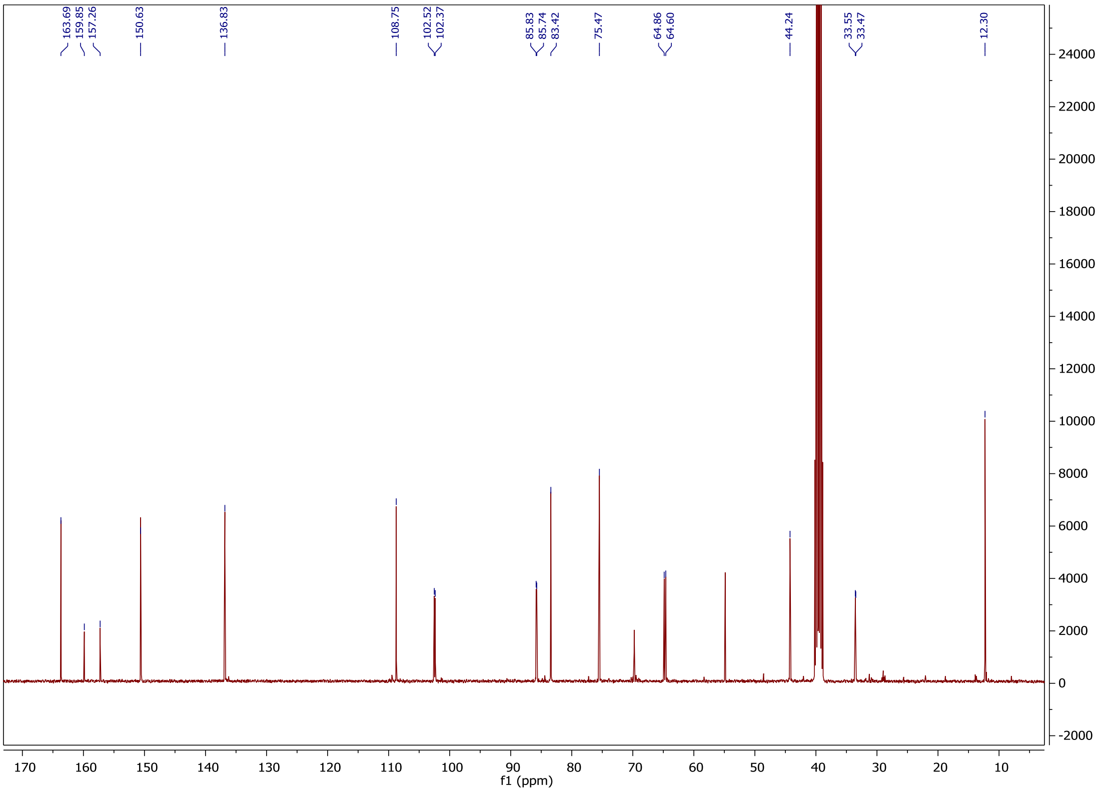

**Figure S30:** $^{13}$C NMR (101 MHz, DMSO-$d_6$) spectra of **8**.

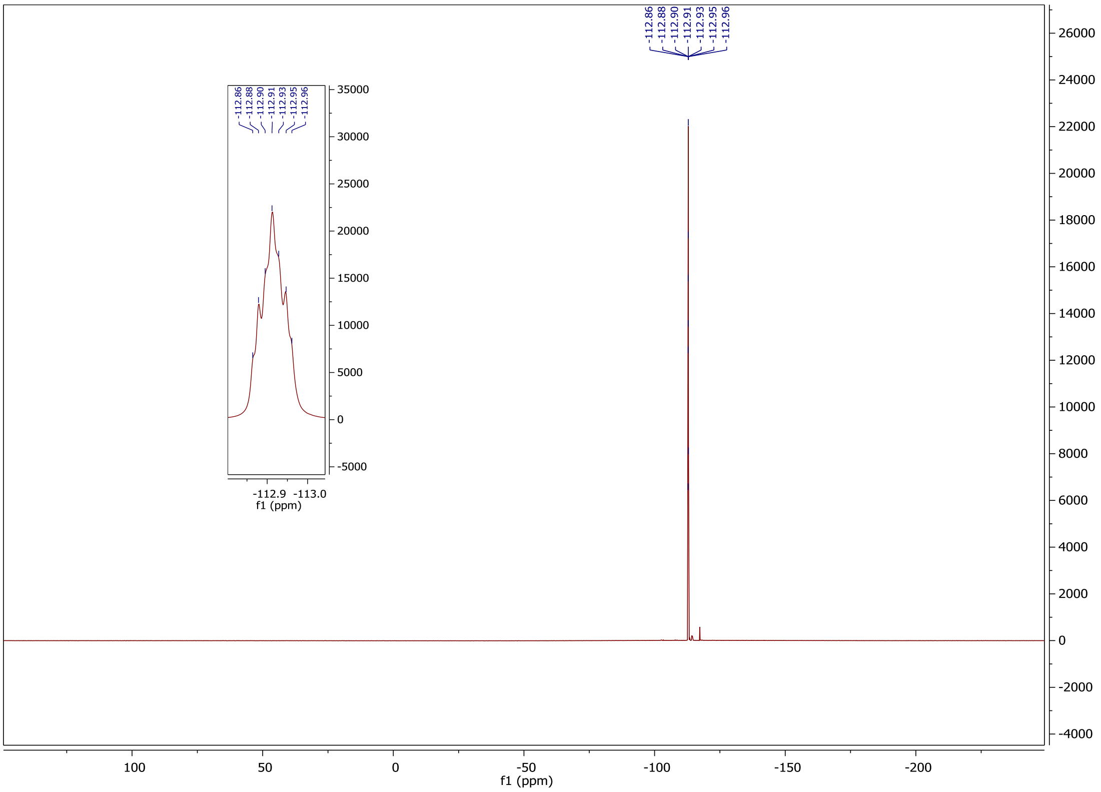


**Figure S31:** $^{19}$F NMR (376 MHz, DMSO-$d_6$) spectra of **8**.

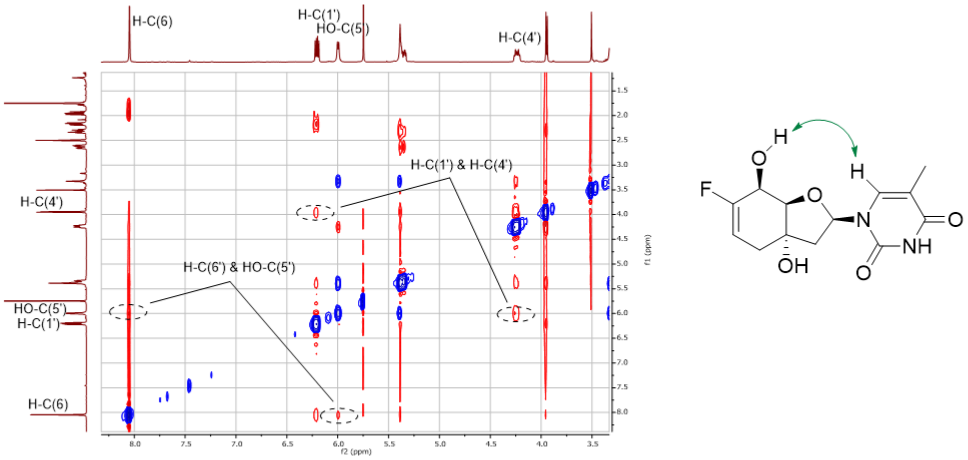


**Figure S32:** $^{1}$H,$^{1}$H-ROESY spectrum (400 MHz, DMSO-$d_6$) of **8**.

**1-{(3'*S*,5'*S*)-2'-Deoxy-6'-fluoro-5'-*O*-[(4,4'-dimethyoxytriphenyl)methyl]-3',5'-prop-6'-eno-β-D-ribofuranosyl}thymine (9)**

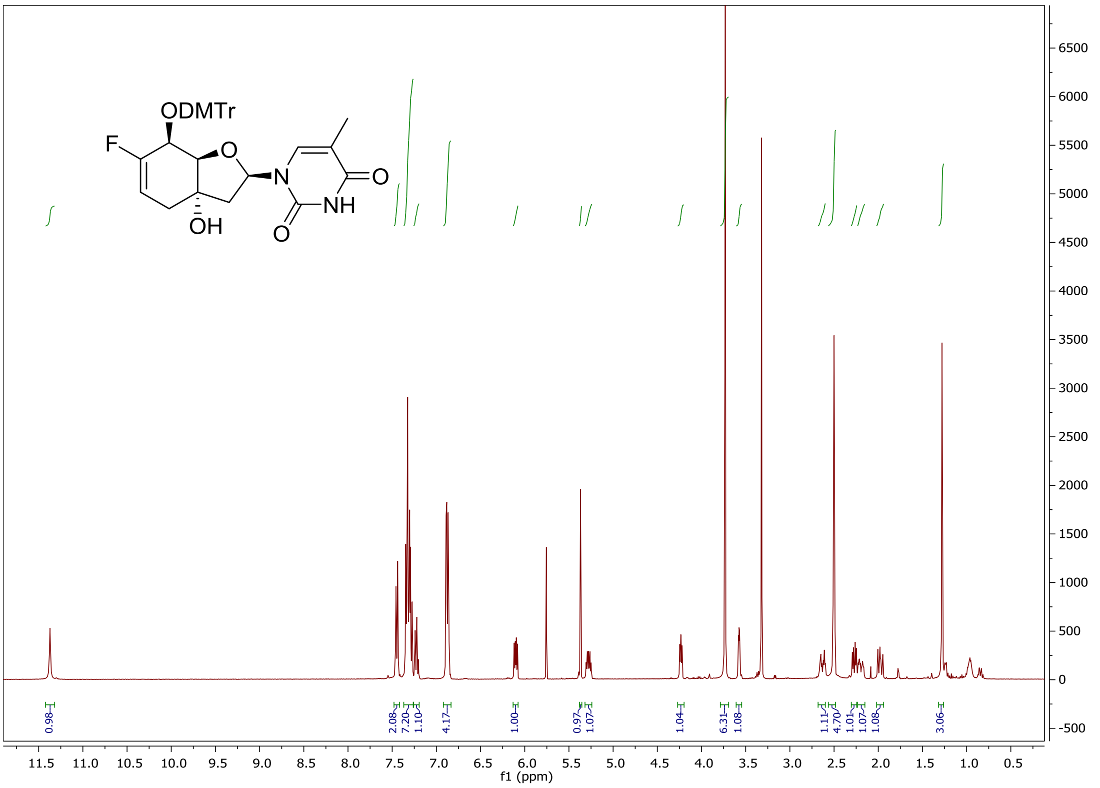

**Figure S33:** $^{1}$H NMR (400 MHz, DMSO-$d_6$) spectra of **9**.

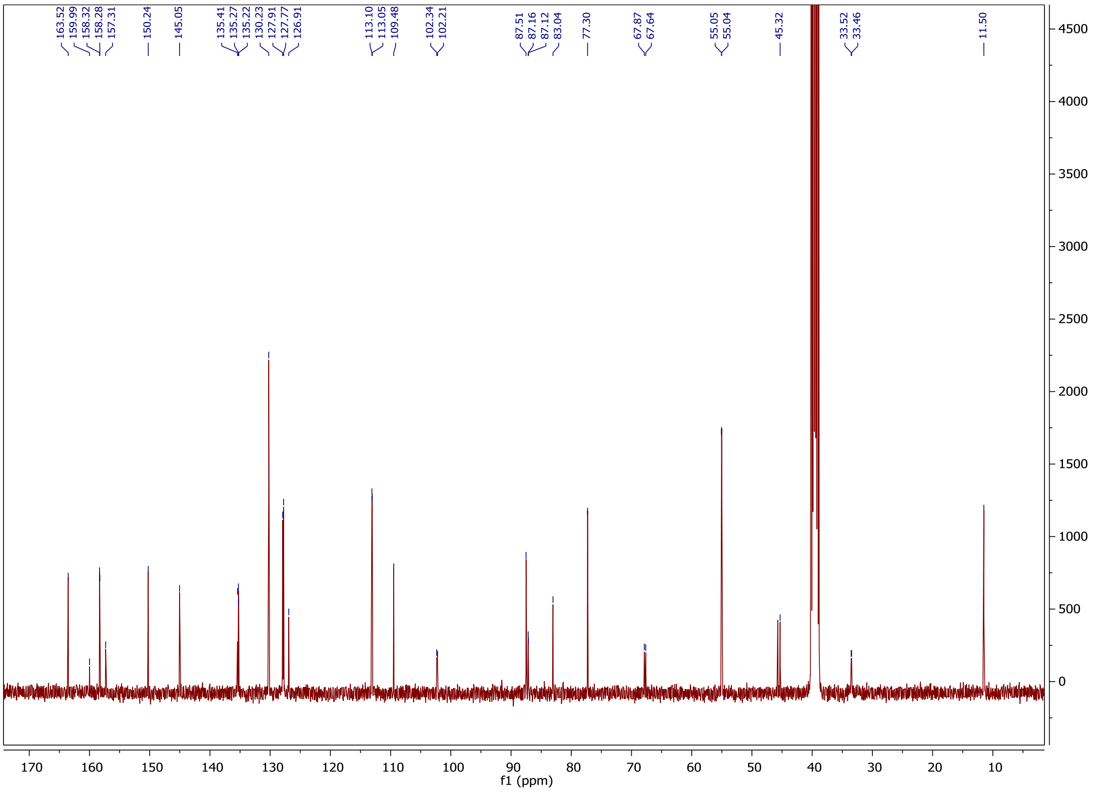

**Figure S34:** $^{13}$C NMR (101 MHz, DMSO-$d_6$) spectra of **9**.

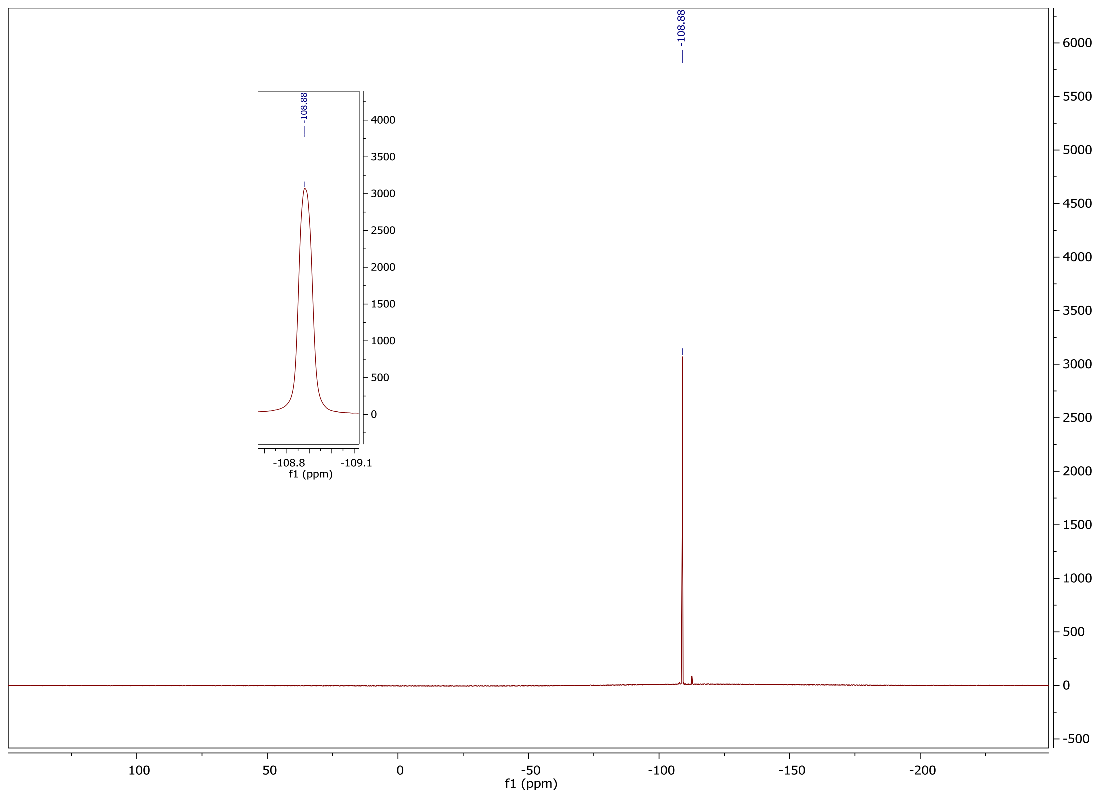

**Figure S35:** $^{19}$F NMR (376 MHz, DMSO-$d_6$) spectra of **9**.

**1-{(3’*S*,5’*S*)-3’-*O*-[(2-Cyanoethoxy)-diisopropylaminophosphanyl]-2’-deoxy-6’-fluoro-5’-*O*-[(4,4’-dimethyoxytriphenyl)methyl]-3’,5’-prop-6’-eno-β-D-ribofuranosyl}thymine (10)**

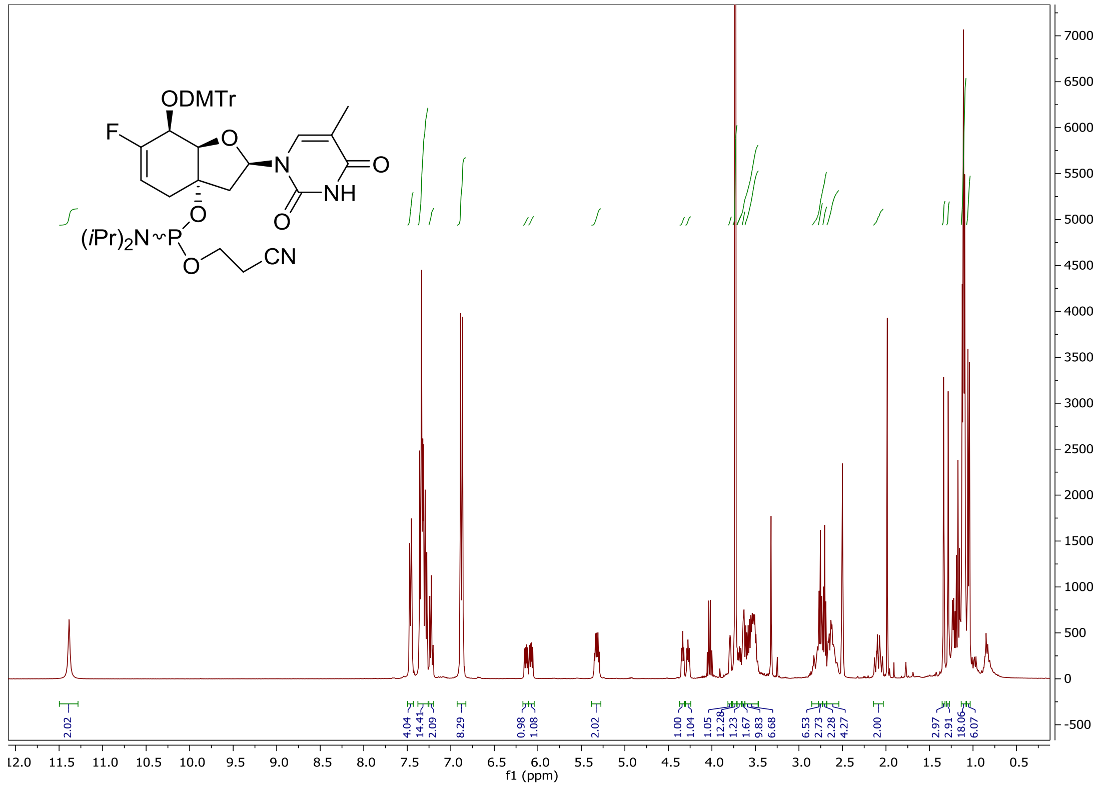

**Figure S36:** $^{1}$H NMR (400 MHz, DMSO-$d_6$) spectra of **10**.

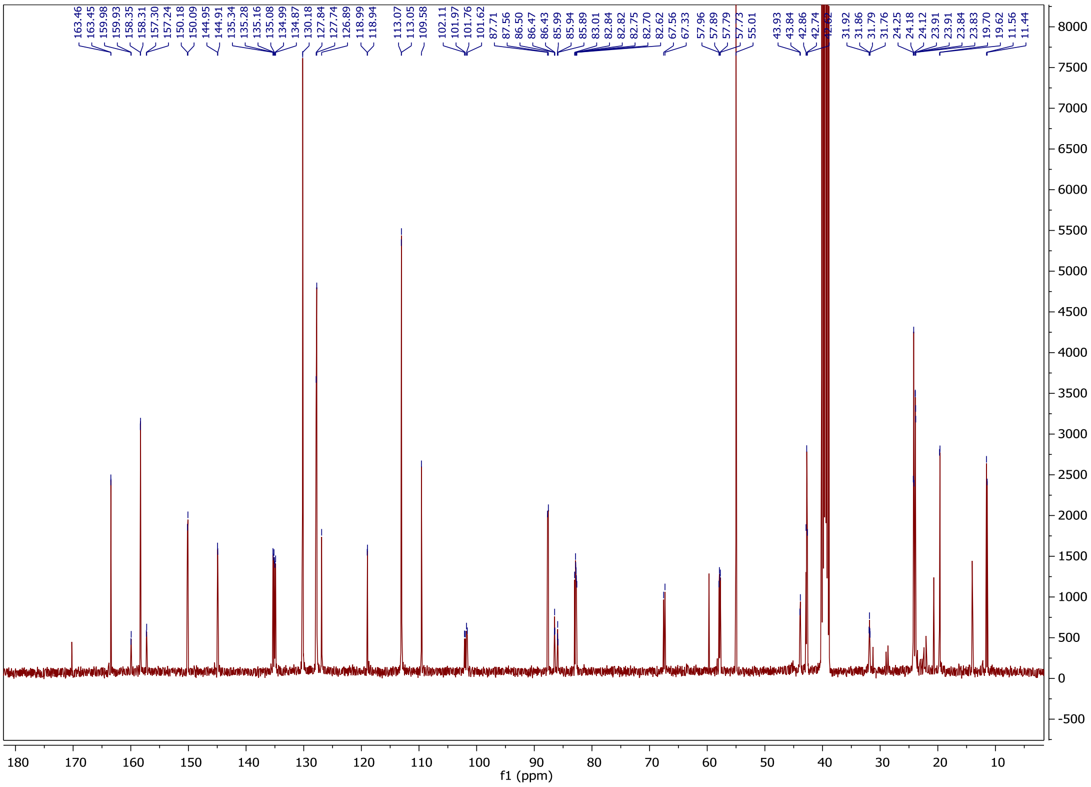

**Figure S37:** $^{13}$C NMR (101 MHz, DMSO-$d_6$) spectra of **10**.

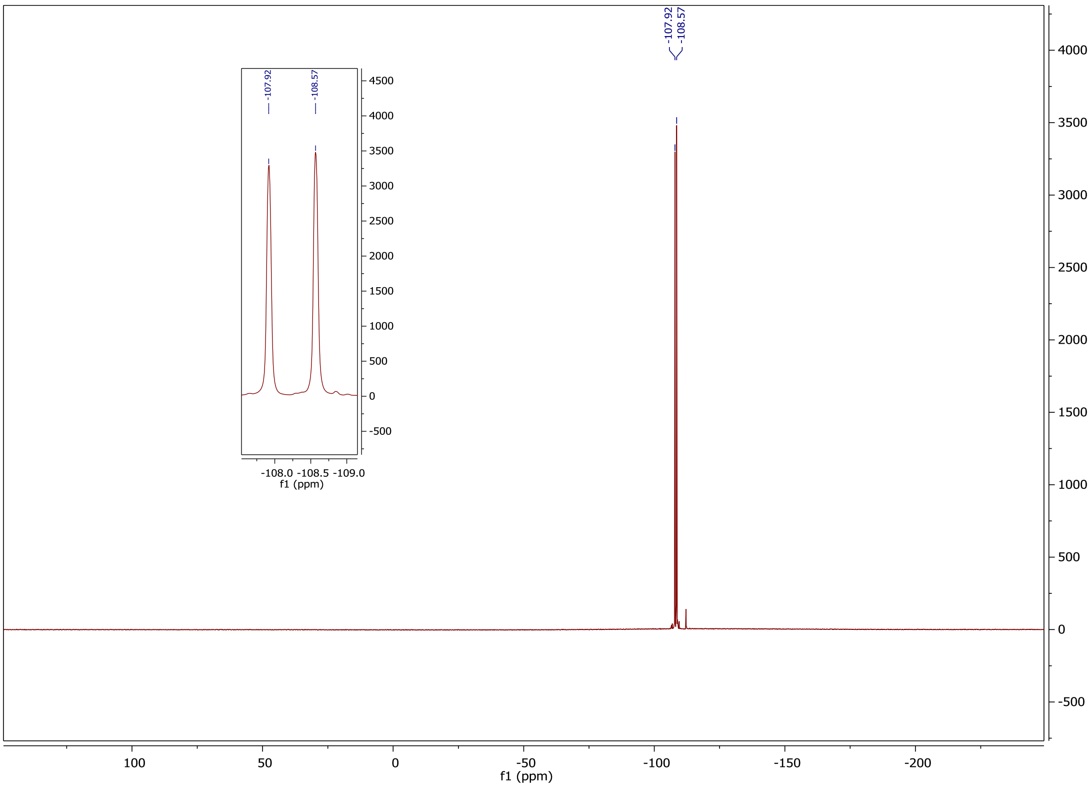

**Figure S38:** $^{19}$F NMR (376 MHz, DMSO-$d_6$) spectra of **10**.

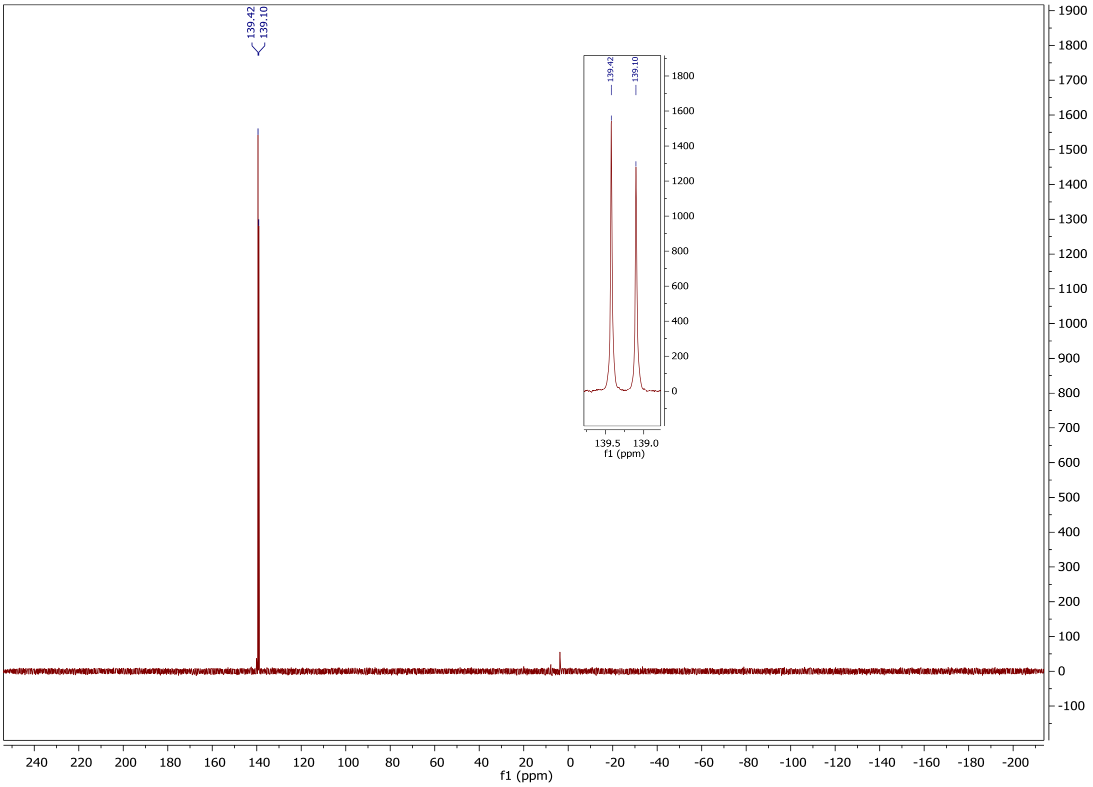


**Figure S39:** $^{31}$P NMR (162 MHz, DMSO-$d_6$) spectra of **10**.

### (1*S*,2*R*,4*S*,6*S*,8*R* and *S*)-8-Acteyloxy-2-[(triethylsilyl)oxy]-3,3-difluroro-6-[(trimethylsilyl)oxy]-9-oxatricyclo[4.3.0$^{1,6}$.0$^{2,4}$]nonane (11α/β)

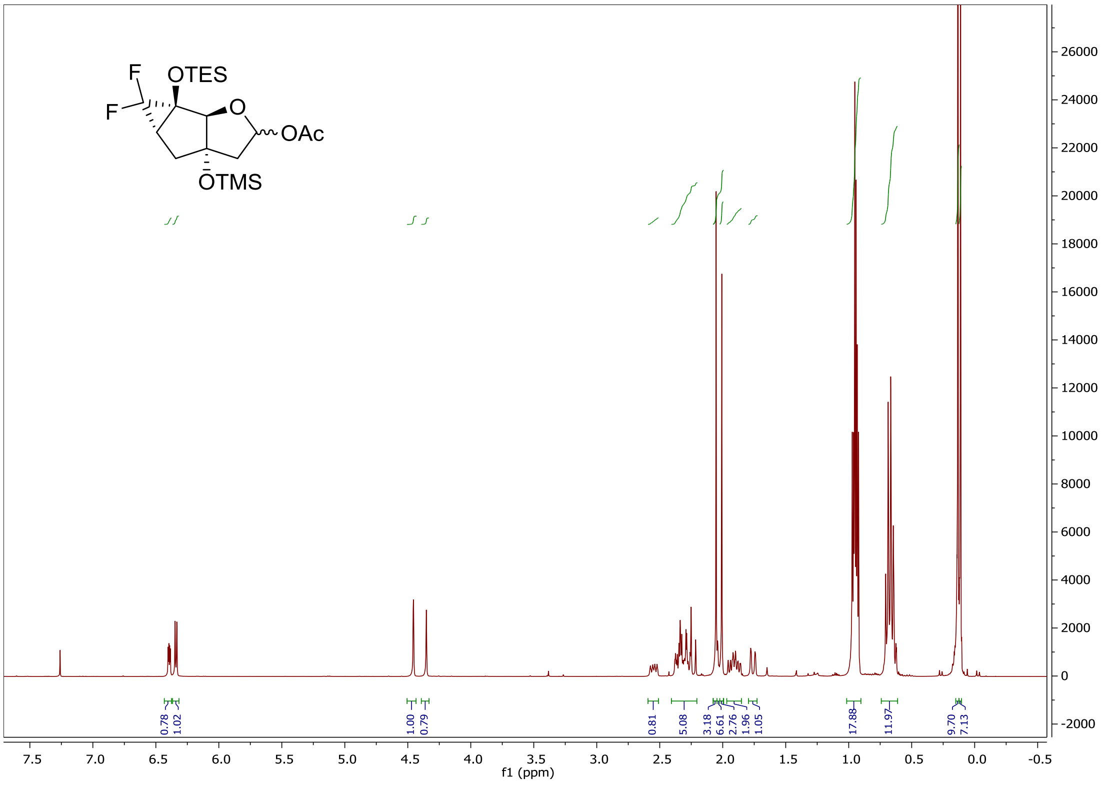


**Figure S40:** $^{1}$H NMR (400 MHz, $CDCl_3$) spectra of **11α/β**.

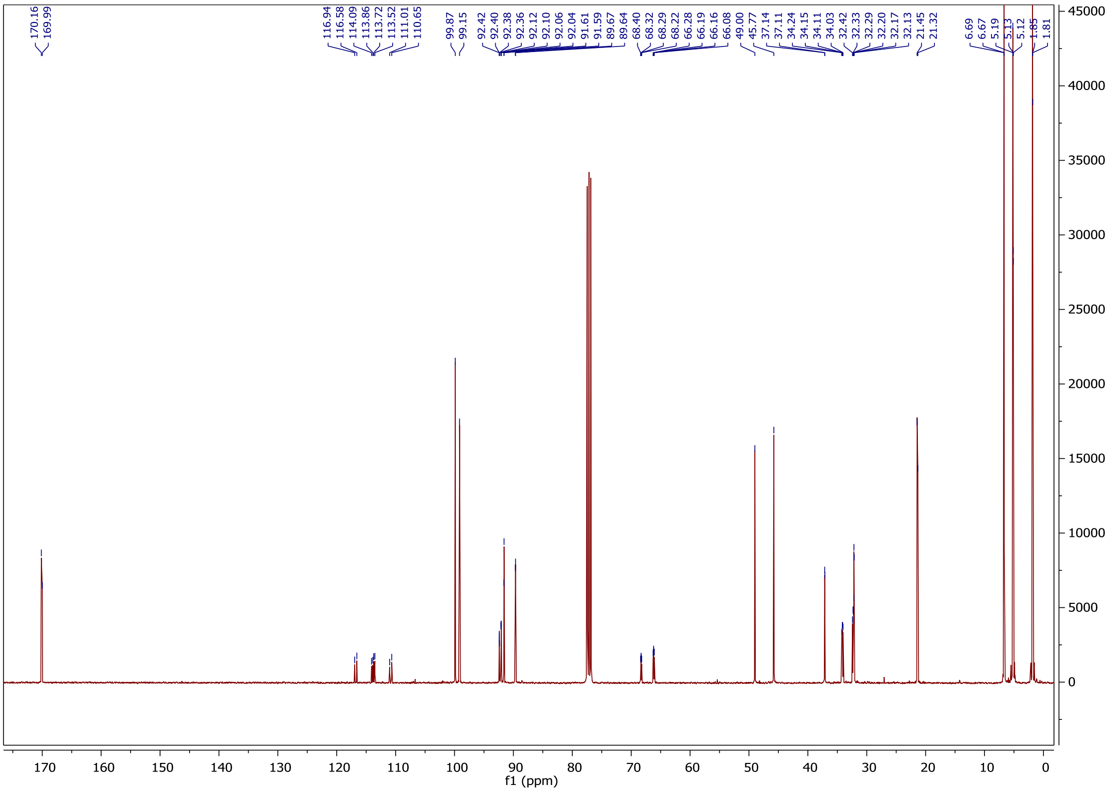


**Figure S41:** $^{13}$C NMR (101 MHz, $CDCl_3$) spectra of **11α/β**.

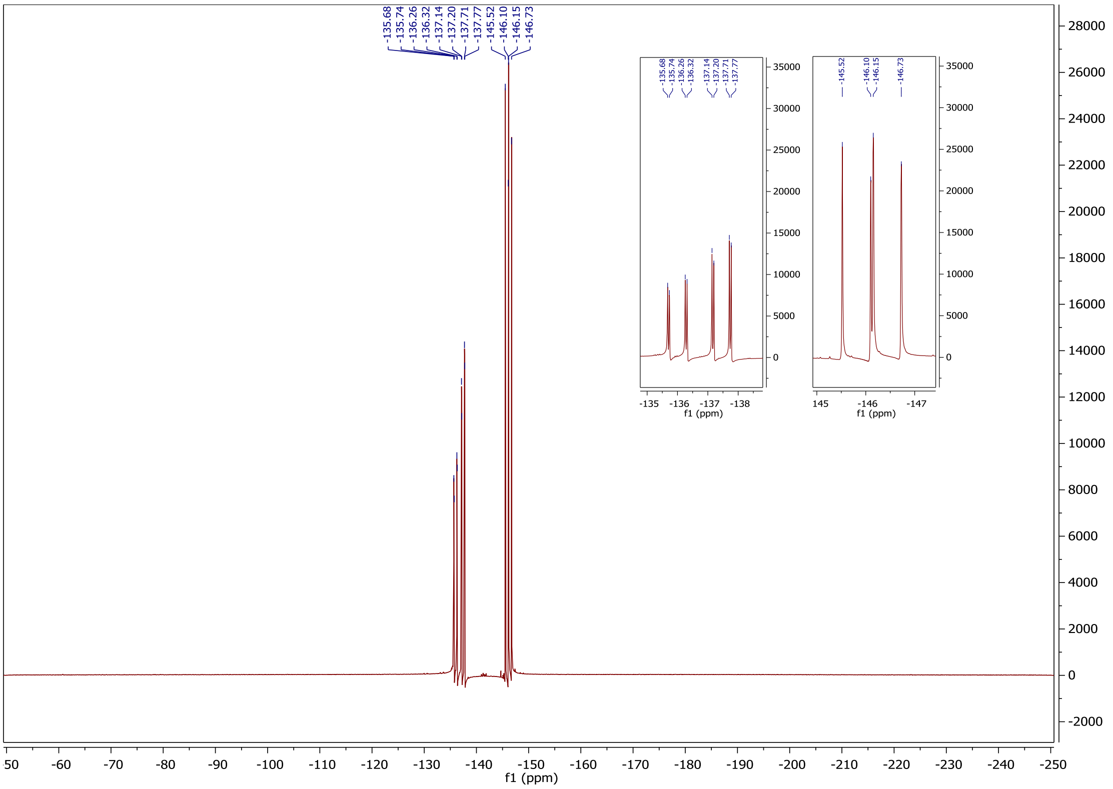


**Figure S42:** $^{19}$F NMR (282 MHz, $CDCl_3$) spectra of **11α/β**.

**1-[(3’*S*,5’*R*,6’*S*)-2’-Deoxy-3’,5’-ethano-5’-*O*-triethylsilyl-8’,8’-dilfluoro-5’,6’-methano-3’-*O*-trimethylsilyl-β-D-ribofuranosyl]-4-*N*-benzoyl-cytosine (12β)**

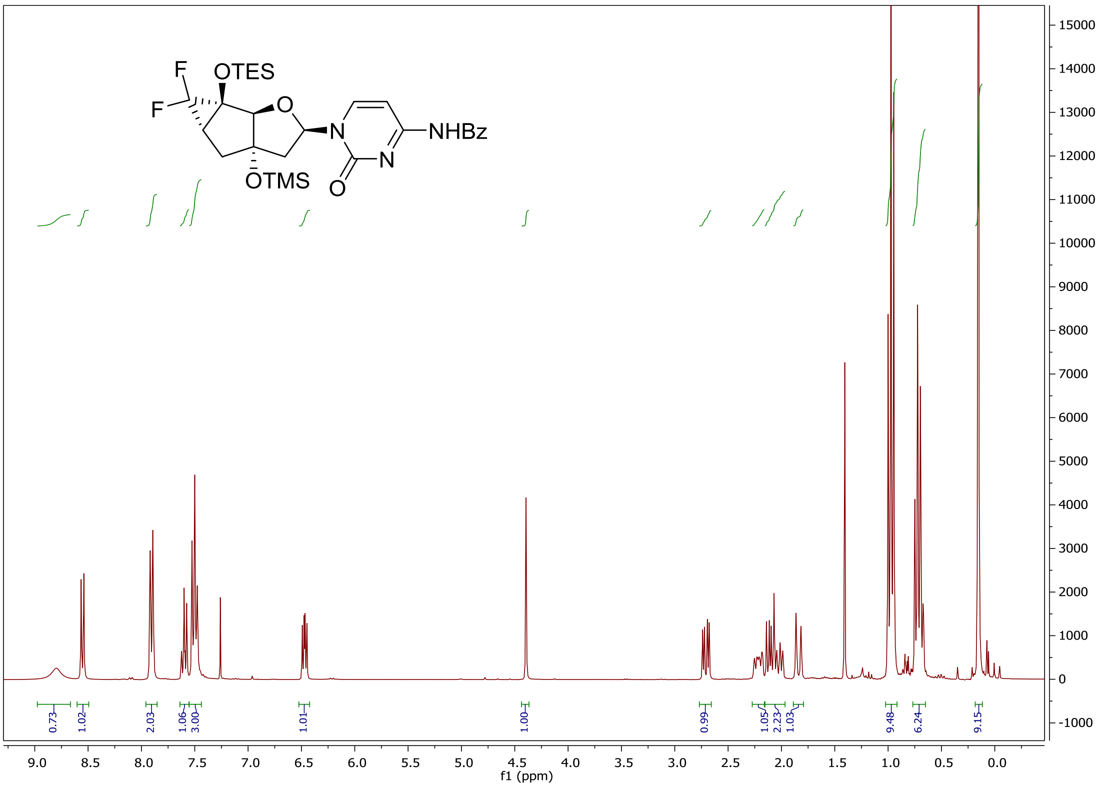


**Figure S43:** $^{1}$H NMR (300 MHz, $CDCl_3$) spectra of **12β**.

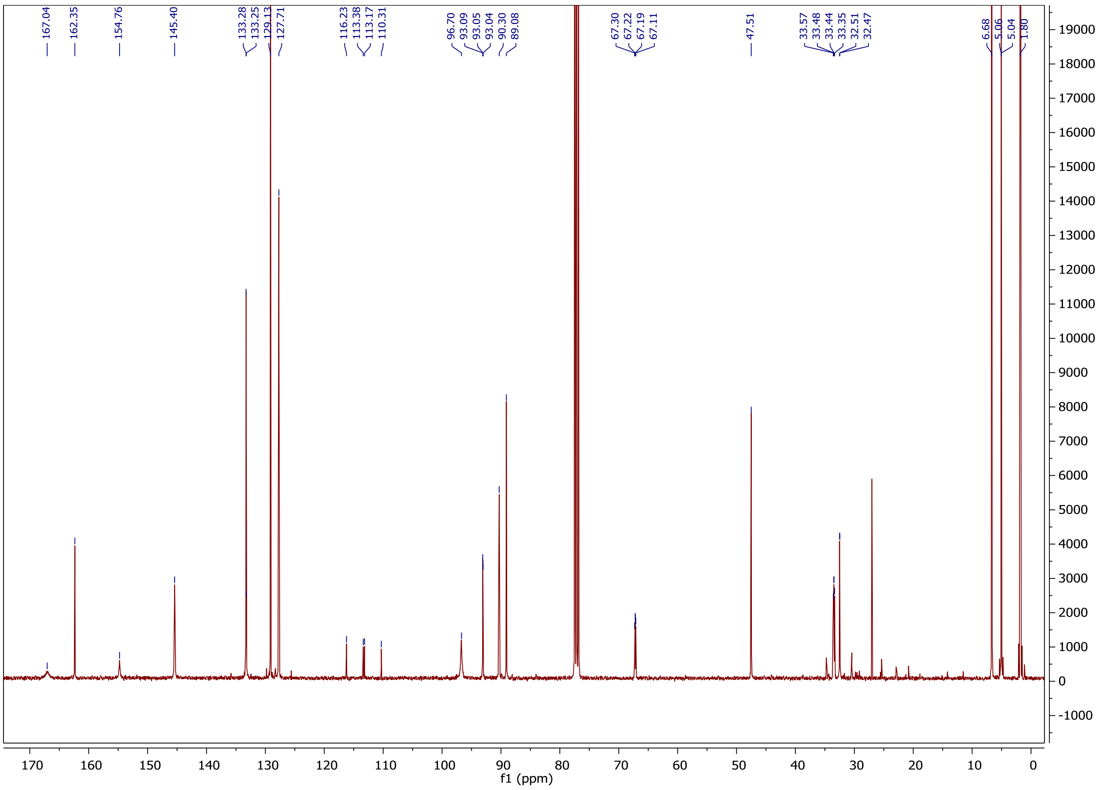


**Figure S44:** $^{13}$C NMR (101 MHz, $CDCl_3$) spectra of **12β**.

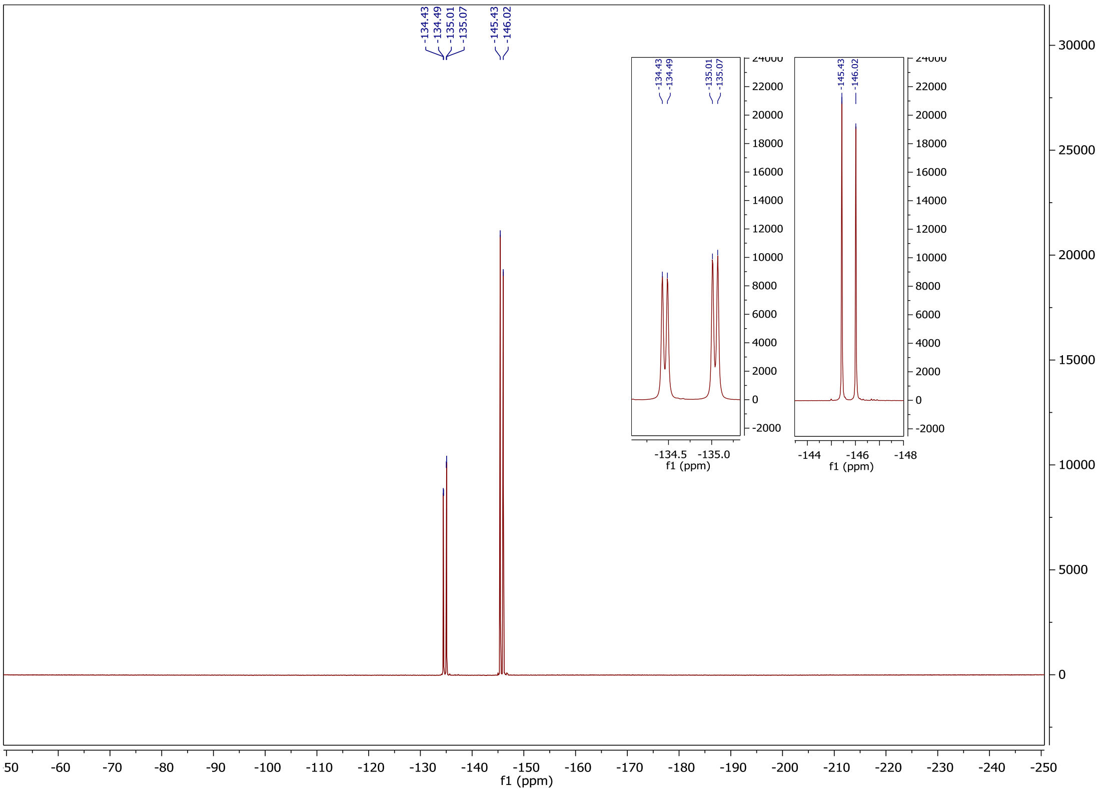

**Figure S45:** $^{19}$F NMR (282 MHz, $CDCl_3$) spectra of **12β**.

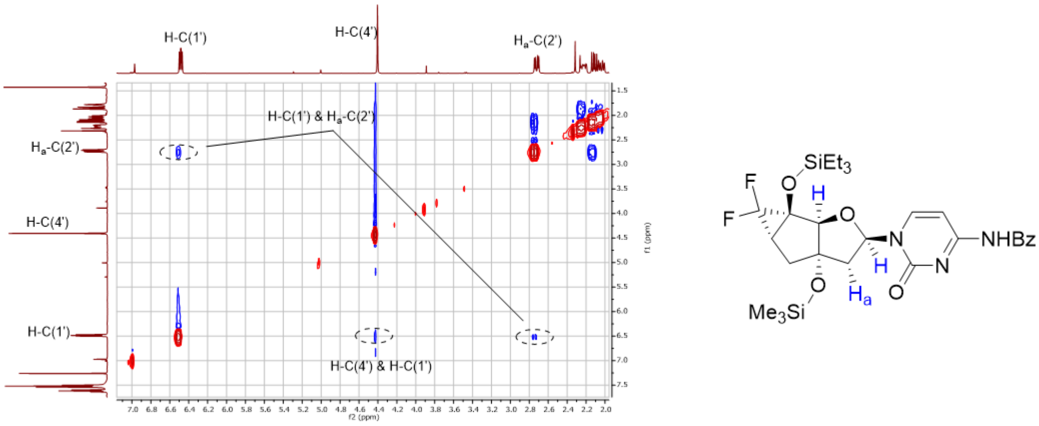


**Figure S46:** $^{1}$H,$^{1}$H-ROESY spectrum (400 MHz, $CDCl_3$) of **12β**.

**1-[(3'*S*)-2'-Deoxy-6'-fluoro-5'-on-3',5'-prop-6'-eno-β-D-ribofuranosyl]-4-*N*-benzoyl-cytosine (13)**

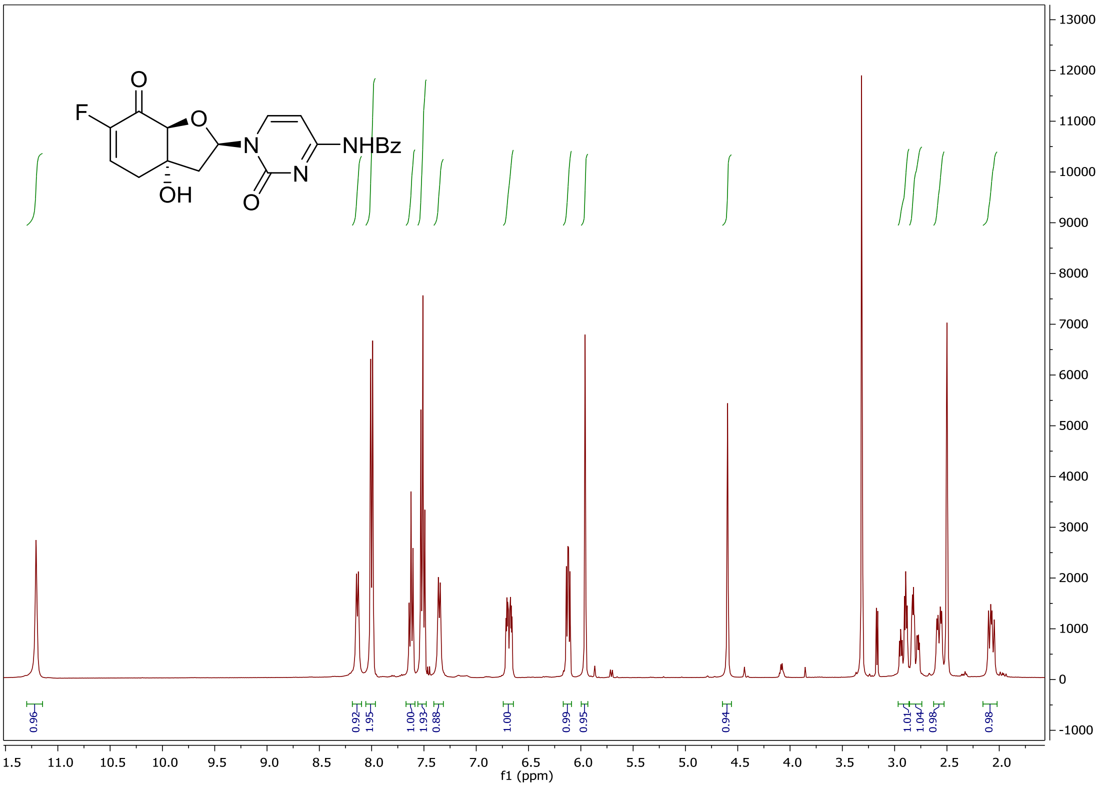

**Figure S47:** $^{1}$H NMR (400 MHz, DMSO-$d_6$) spectra of **13**.

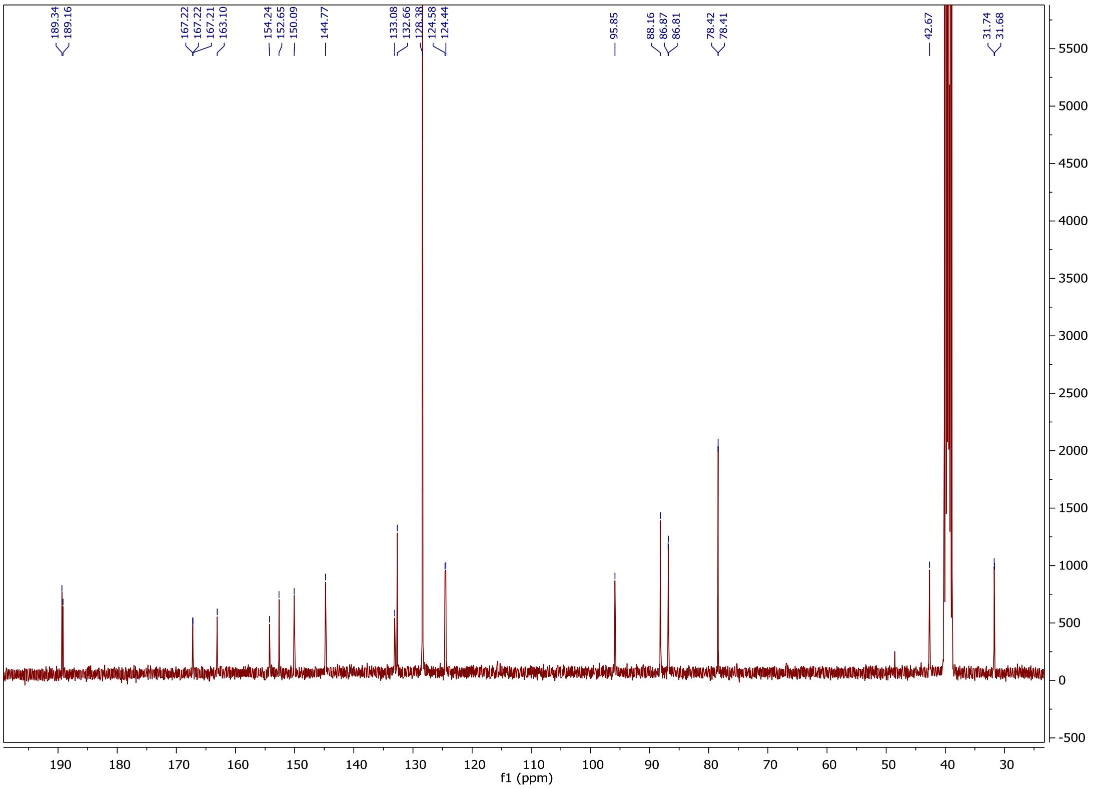

**Figure S48:** $^{13}$C NMR (101 MHz, DMSO-$d_6$) spectra of **13**.

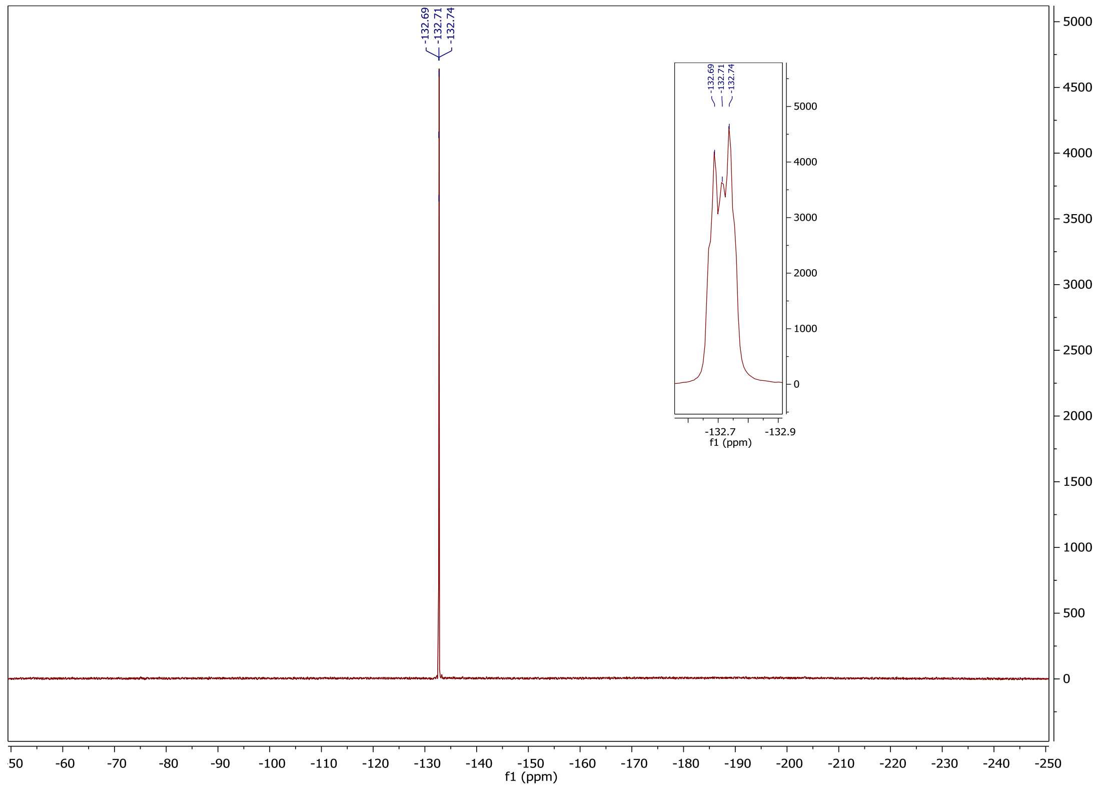


**Figure S49:** $^{19}$F NMR (282 MHz, DMSO-$d_6$) spectra of **13**.

### 1-[(3’*S*,5’*S*)-2’-Deoxy-6’-fluoro-3’,5’-prop-6’-eno-β-D-ribofuranosyl]-4-*N*-benzoyl-cytosine (14)

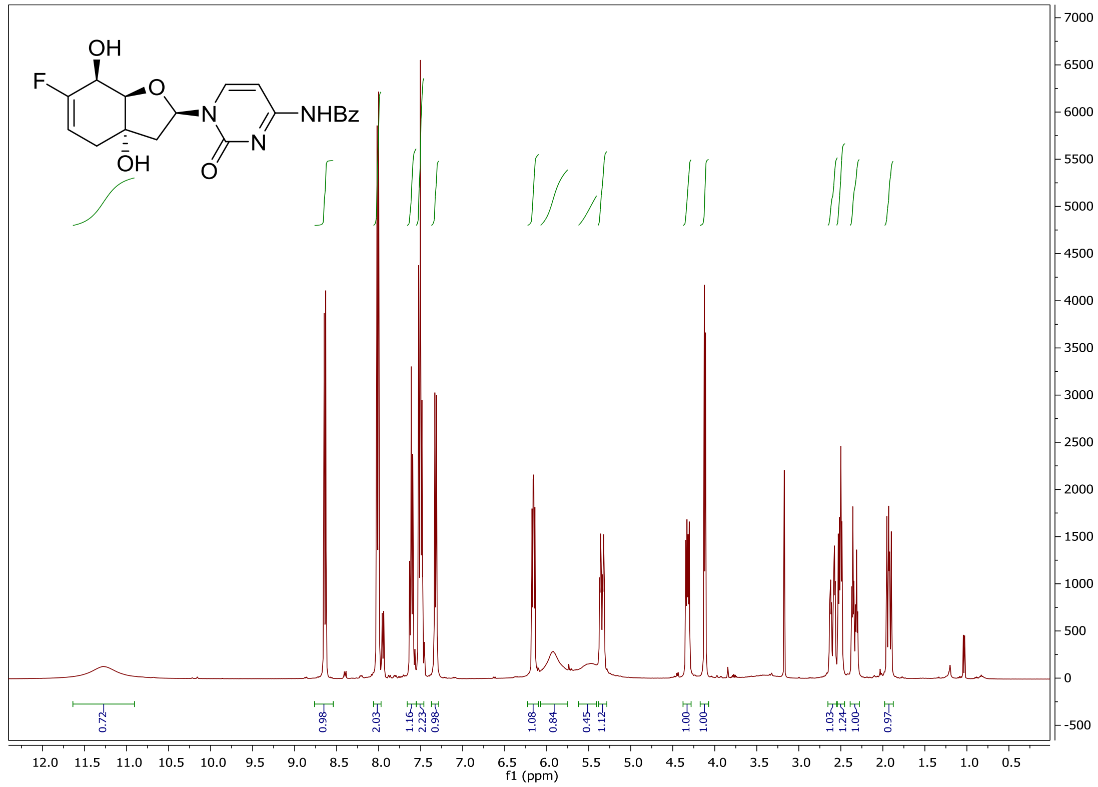


**Figure S50:** $^{1}$H NMR (400 MHz, DMSO-$d_6$) spectra of **14**.

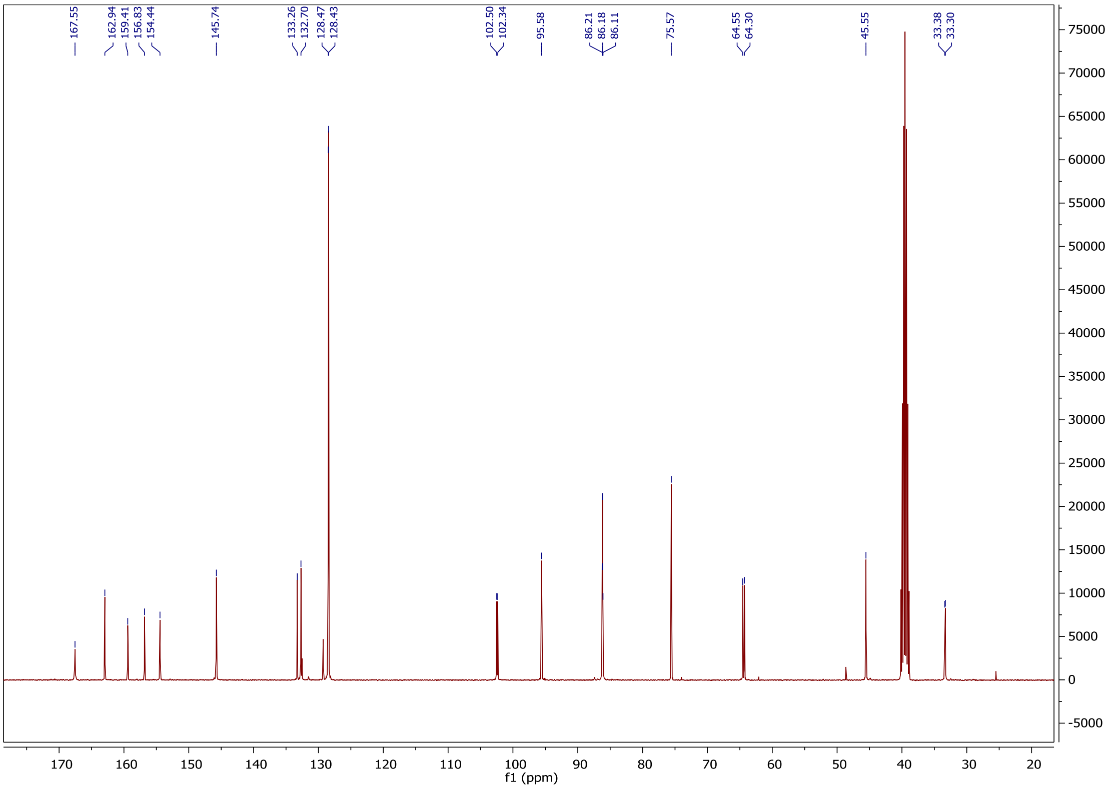


**Figure S51:** $^{13}$C NMR (101 MHz, DMSO-$d_6$) spectra of **14**.

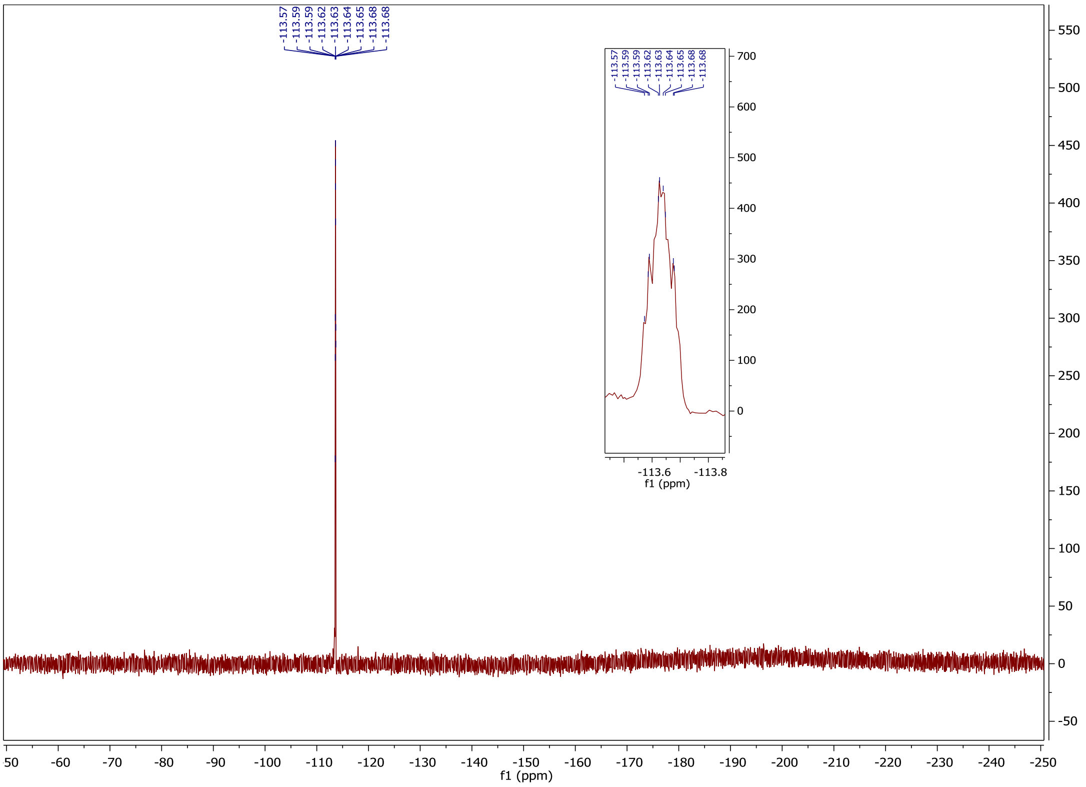


**Figure S52:** $^{19}$F NMR (282 MHz, DMSO-$d_6$) spectra of **14**.

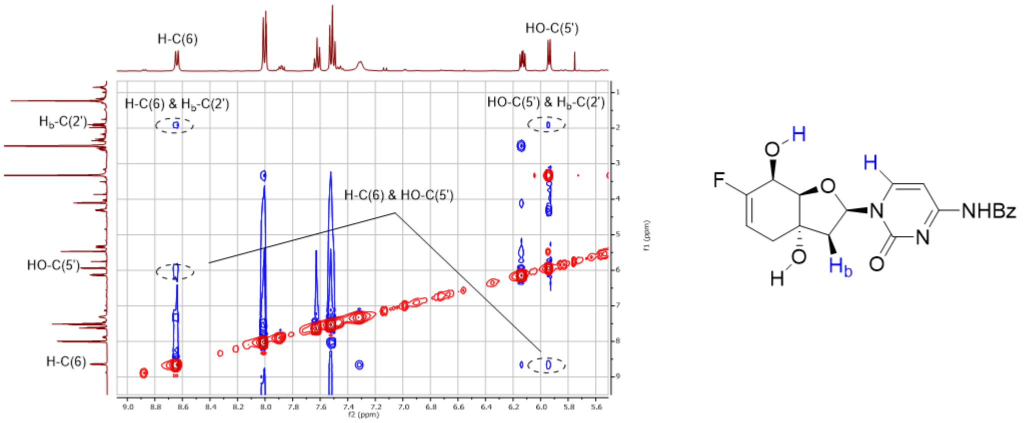


**Figure S53:** $^{1}$H,$^{1}$H-ROESY spectrum (400 MHz, DMSO-$d_6$) of **14**.

## 1-{(3’*S*,5’*S*)-3’-*O*-[(2-Cyanoethoxy)-diisopropylaminophosphanyl]-2’-deoxy-6’-fluoro-5’-*O*-[(4,4’-dimethyoxytriphenyl)methyl]-3’,5’-prop-6’-eno-β-D-ribofuranosyl}-4-*N*-benzoyl-cytosine (15)

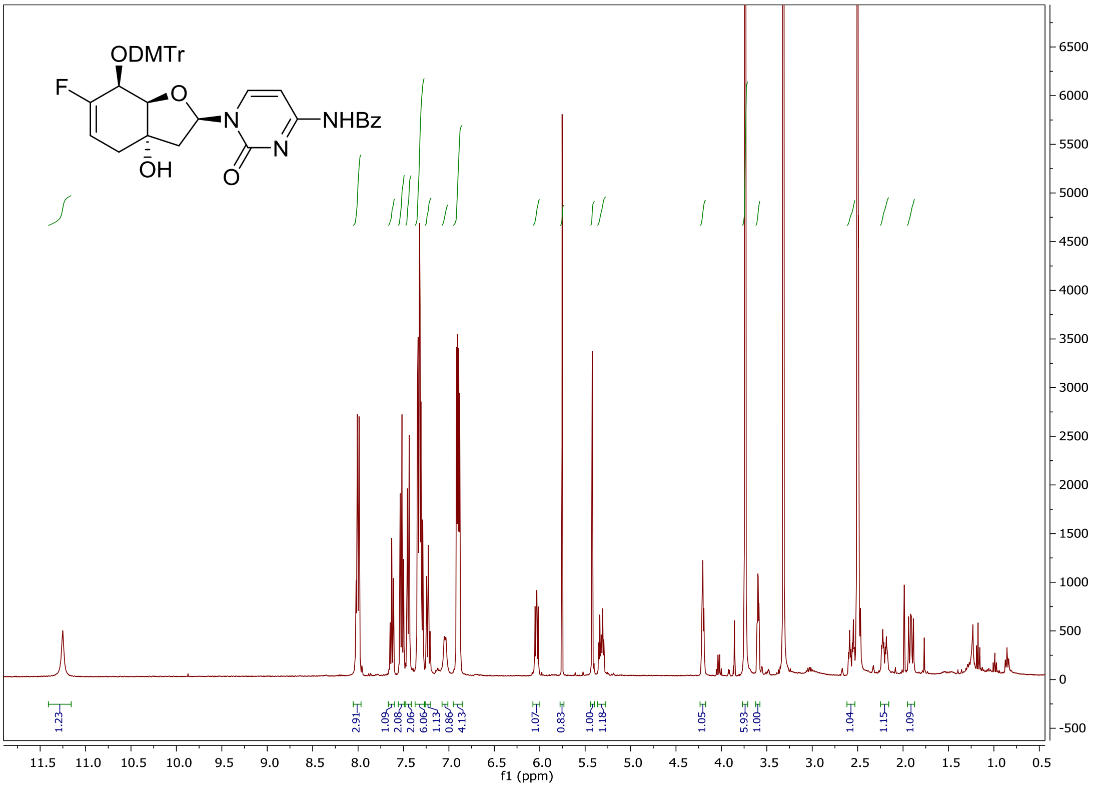


**Figure S54:** $^{1}$H NMR (400 MHz, DMSO-$d_6$) spectra of **15**.

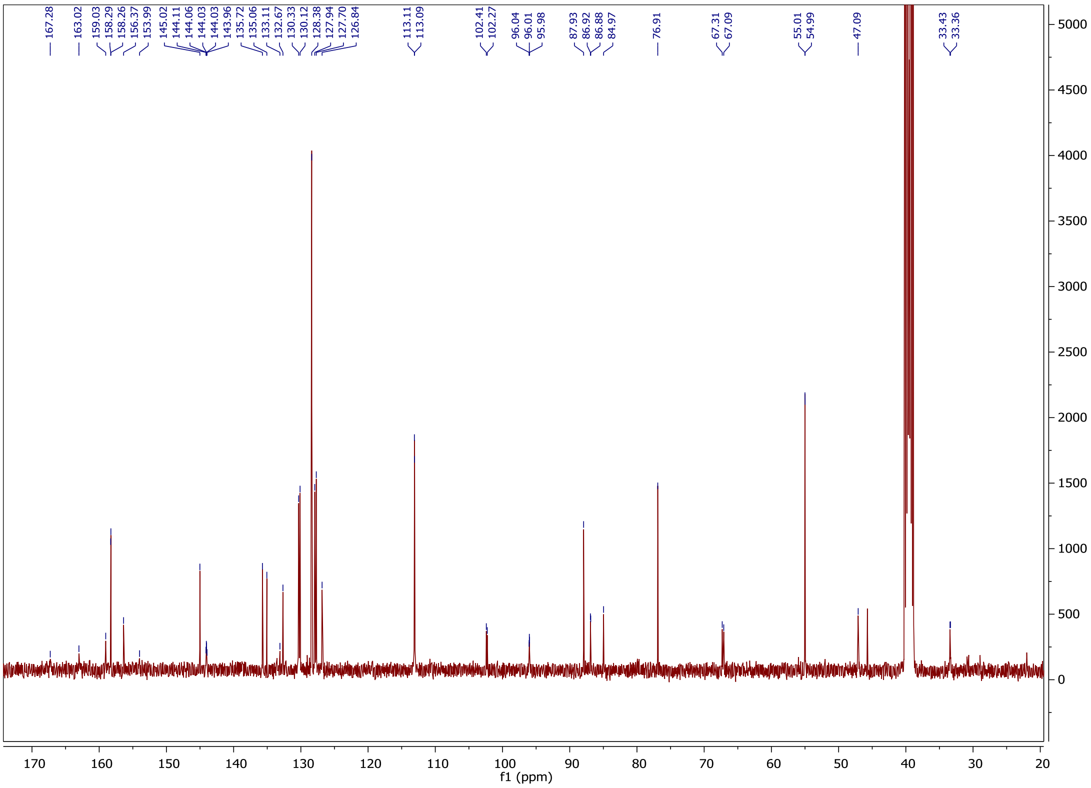

**Figure S55:** $^{13}$C NMR (101 MHz, DMSO-$d_6$) spectra of **15**.

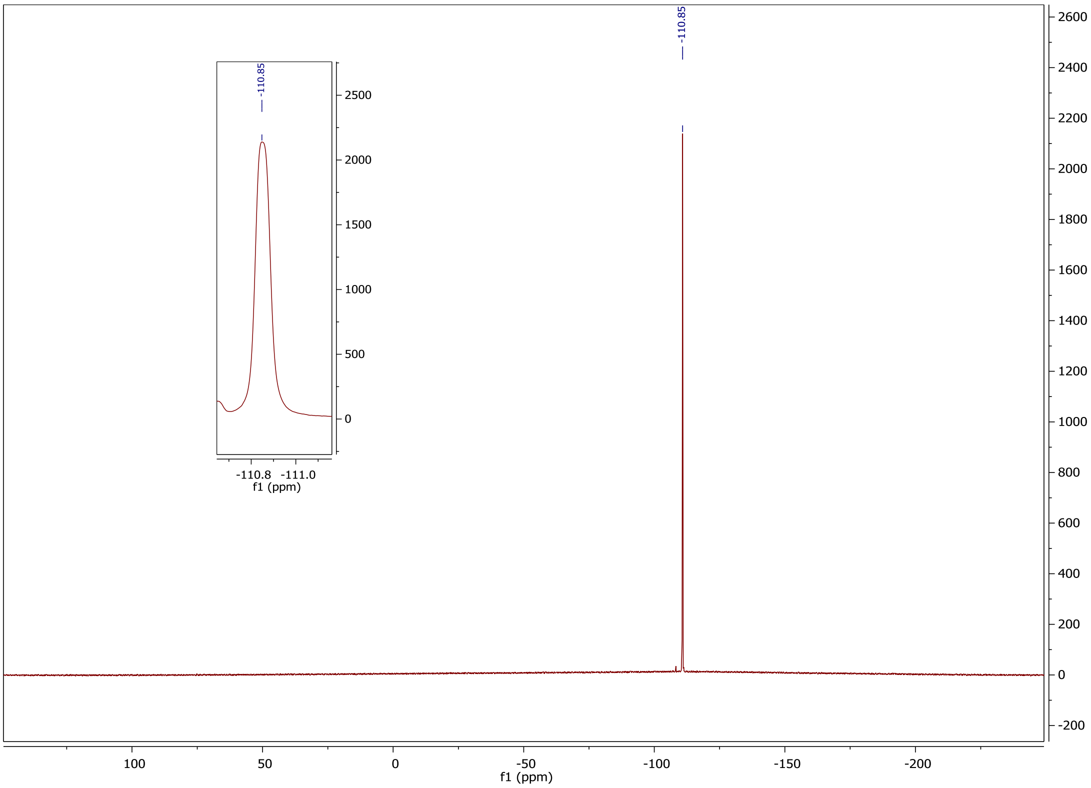

**Figure S56:** $^{19}$F NMR (376 MHz, DMSO-$d_6$) spectra of **15**.

**1-{(3'*S*,5'*S*)-3'-*O*-[(2-Cyanoethoxy)-diisopropylaminophosphanyl]-2'-deoxy-6'-fluoro-5'-*O*-[(4,4'-dimethyoxytriphenyl)methyl]-3',5'-prop-6'-eno-β-D-ribofuranosyl}-4-*N*-benzoyl-cytosine (16)**

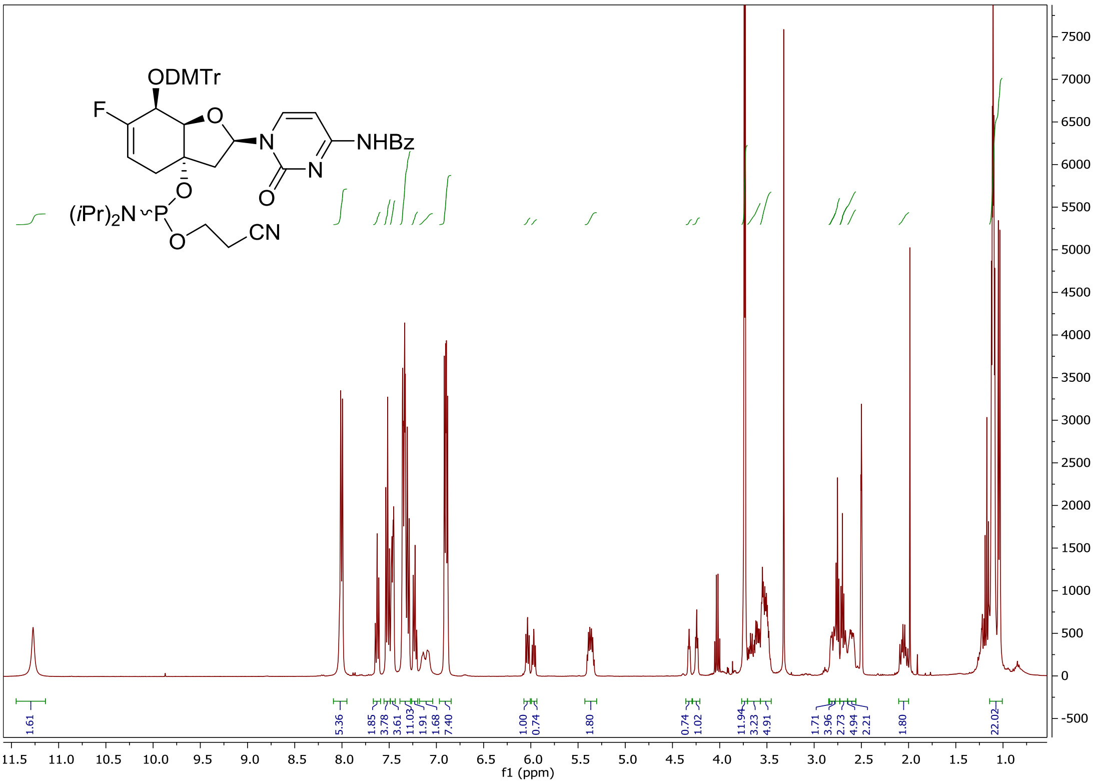

**Figure S57:** $^1$H NMR (400 MHz, DMSO-$d_6$) spectra of **16**.

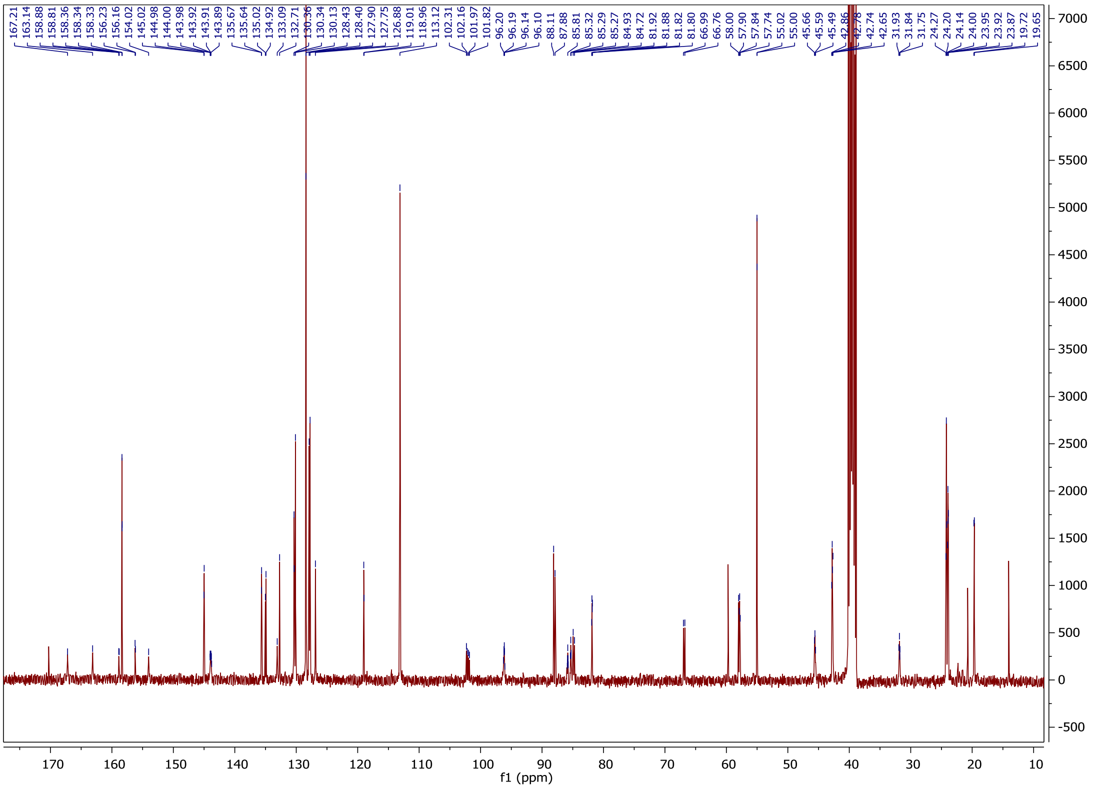

**Figure S58:** $^{13}$C NMR (101 MHz, DMSO-$d_6$) spectra of **16**.

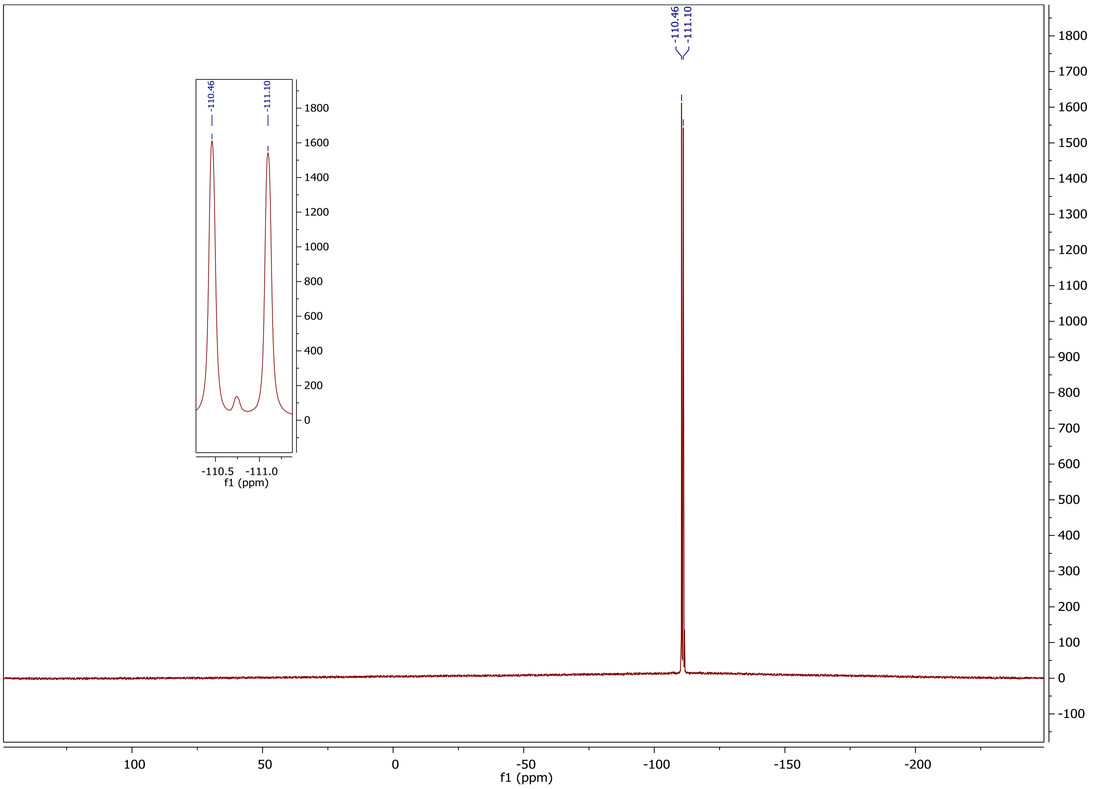


**Figure S59:** $^{19}$F NMR (376 MHz, DMSO-$d_6$) spectra of **16**.

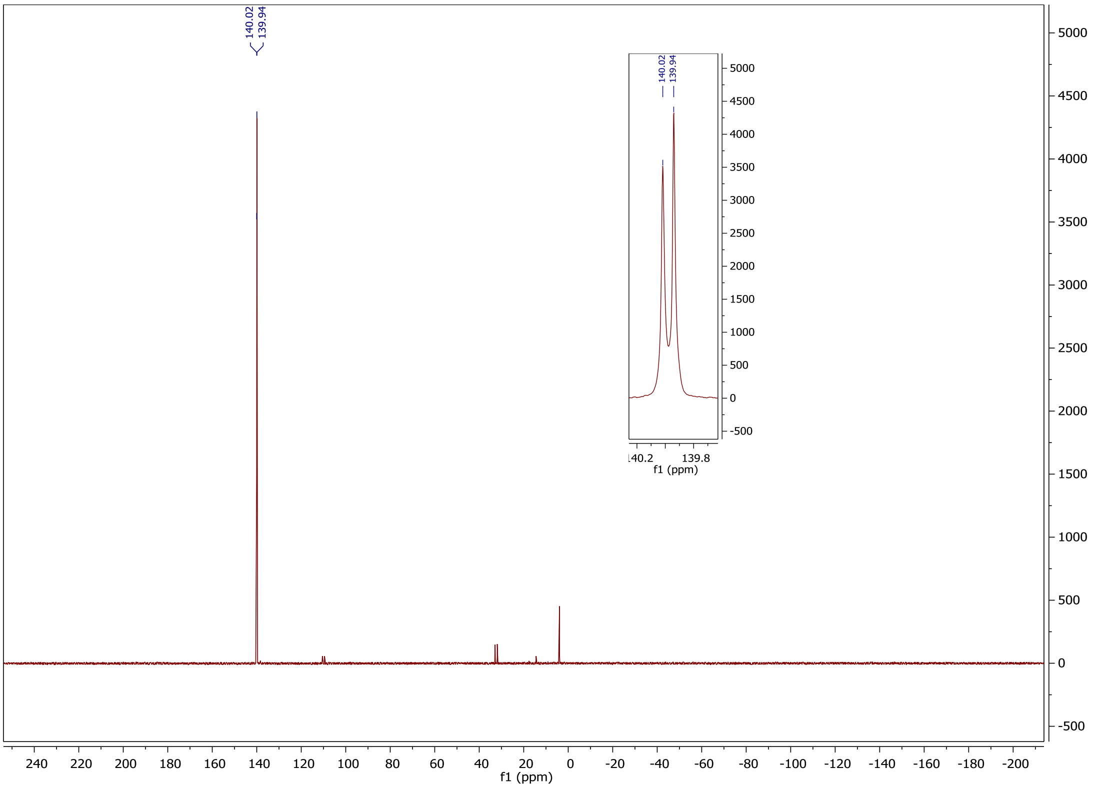


**Figure S60:** $^{31}$P NMR (162 MHz, DMSO-$d_6$) spectra of **16**

## 10. References


1. *Gaussian 09*, Revision A.02; Gaussian, Inc.: Wallingford, CT, 2009.
2. Van der Spoel, D.; Lindahl, E.; Hess, B.; Groenhof, G.; Mark, A. E.; Berendsen, H. J. C. *J. Comput. Chem.* **2005**, *26*, 1701–1718.
3. Cornell, W. D.; Cieplak, P.; Bayly, C. I.; Gould, I. R.; Merz, Kenneth M., J.; Ferguson, D. M.; Spellmeyer, D. C.; Fox, T.; Caldwell, J. W.; Kollman, P. A. *J. Am. Chem. Soc.* **1995**, *117*, 5179–5197.
4. Dupradeau, F.-Y.; Pigache, A.; Zaffran, T.; Savineau, C.; Lelong, R.; Grivel, N.; Lelong, D.; Rosanski, W.; Cieplak, P. *Phys. Chem. Chem. Phys.* **2010**, *12*, 7821–7839.
5. Jorgensen, W. L.; Chandrasekhar, J.; Madura, J. D.; Impey, R. W.; Klein, M. L. *J. Chem. Phys.* **1983**, *79*, 926–935.
6. Darden, T.; York, D.; Pedersen, L. *J. Chem. Phys.* **1993**, *98*, 10089–10092.
7. Hess, B.; Bekker, H.; Berendsen, H. J. C.; Fraaije, J. G. E. M. *J. Comput. Chem.* **1997**, *18*, 1463–1472.
8. Kumar, R.; Grubmüller, H. *Bioinformatics* **2015**, *31*, 2583–2585.
9. Luisier, S.; Leumann, C. J. *Heterocycles* **2010**, *82*, 775–790.
10. Luisier, S. Screening the structural and functional properties of bicyclo-DNA. Ph.D. Thesis, University of Bern, Switzerland, 2008.
